# The Gut Epithelial Receptor LRRC19 Promotes the Recruitment of Immune Cells and Gut Inflammation

**DOI:** 10.1016/j.celrep.2015.12.070

**Published:** 2016-01-14

**Authors:** Shuisong Cao, Xiaomin Su, Benhua Zeng, Hui Yan, Yugang Huang, Enlin Wang, Huan Yun, Yuan Zhang, Feifei Liu, Wenxia Li, Hong Wei, Yongzhe Che, Rongcun Yang

**Affiliations:** 1 Department of Immunology, Nankai University School of Medicine, Nankai University, Tianjin 300071, China; 2 State Key Laboratory of Medicinal Chemical Biology, Nankai University, Tianjin 300071, China; 3 Key Laboratory of Bioactive Materials, Ministry of Education, Nankai University, Tianjin 300071, China; 4 Department of Laboratory Animal Science, College of Basic Medicine Science, Third Military Medical University, Chongqing 404100, China

## Abstract

Commensal microbes are necessary for a healthy gut immune system. However, the mechanism involving these microbes that establish and maintain gut immune responses is largely unknown. Here, we have found that the gut immune receptor leucine-rich repeat (LRR) C19 is involved in host-microbiota interactions. LRRC19 deficiency not only impairs the gut immune system but also reduces inflammatory responses in gut tissues. We demonstrate that the LRRC19-associated chemokines CCL6, CCL9, CXCL9, and CXCL10 play a critical role in immune cell recruitment and intestinal inflammation. The expression of these chemokines is associated with regenerating islet-derived (REG) protein-mediated microbiotas. We also found that the expression of REGs may be regulated by gut *Lactobacillus* through LRRC19-mediated activation of NF-κB. Therefore, our study establishes a regulatory axis of LRRC19, REGs, altered microbiotas, and chemokines for the recruitment of immune cells and the regulation of intestinal inflammation.

## Highlights

• The gut immune receptor LRRC19 is involved in host-microbiota interactions
• LRRC19-associated chemokines control immune cell recruitment and gut inflammation
• Chemokines are regulated by REG protein-mediated gut microbiotas
• *Lactobacillus* may modulate the expression of REG proteins through LRRC19

## Graphical Abstract

**Figure:**
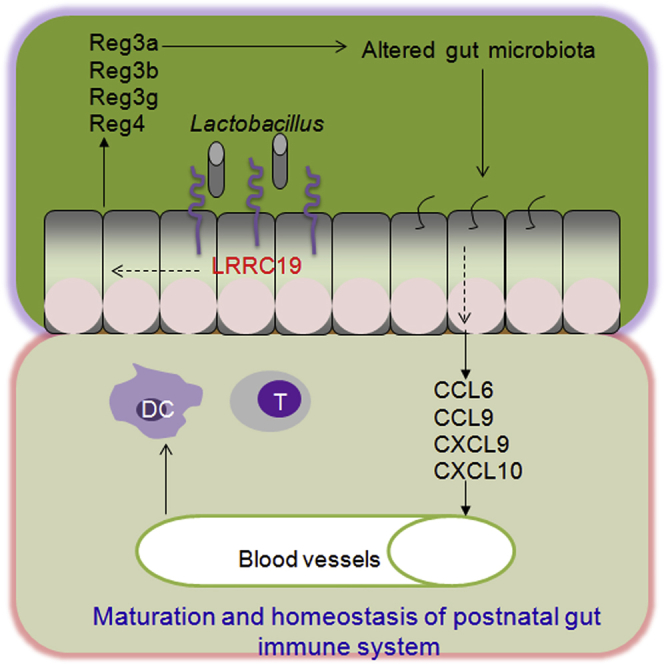

## Teaser

Cao et al. found that the gut epithelial receptor LRRC19 is involved in gut host-microbiota interactions and that it plays a critical role in promoting the recruitment of immune cells and intestinal inflammation.

## Introduction

The gut lymphoid system constitutes the largest part of the whole immune system and includes organized tissues such as Peyer patches (PPs) and the mesenteric lymph nodes (MLNs) as well as lymphoid tissues scattered throughout the lamina propria (LP) and the epithelium of the mucosa (Mowat and Viney, 1997). This gut immune system contains both innate immune cells, such as dendritic cells (DCs), macrophages, and immature myeloid cells, and adaptive immune cells, such as CD4 and CD8 lymphocytes and their subsets, including Th1, Th17, regulatory T (Treg), Th17^+^Foxp3^+^, and innate T cells. These cells perform crucial roles in protecting the body from foreign pathogens and establishing the immunological tolerance in the gut tissues. However, the exact mechanisms underlying recruitment, retention, and differentiation of these immune cells in gut tissues are unclear. Studies have suggested that microbe-recognizing molecules expressed on intestinal epithelial cells can mediate gut microbe-host immune cross-talk, integrating and transmitting signals from bacteria to the mucosal innate and adaptive immune cells (Goto and Ivanov, 2013). When the host lacks pathogen-recognizing molecules, such as myeloid differentiation primary response gene 88 (MyD88, an adaptor molecule in the Toll-like receptor [TLR] signaling pathway), nucleotide-binding oligomerization domain-containing protein 2 (NOD2), or NOD1, it is unable to clear invading pathogens from the gut tissue (Jiang et al., 2013, Luddy et al., 2014). However, under normal physiological conditions, intestinal epithelial cells show only low-level expression of these pathogen-recognizing receptors, which are generally unresponsive to TLR stimuli (Abreu et al., 2001, Melmed et al., 2003), suggesting that these receptors are not essential for the establishment and maintenance of the gut immune system (de Kivit et al., 2014).

Commensal bacteria are necessary for gut immune responses and inflammation under normal physiological conditions. These commensal microbes develop together with the gut immune system from birth and play a crucial role in the maturation of the intestinal mucosal immune system of their host (Clemente et al., 2012). Indeed, germ-free (GF) mice differ from normal mice in the number of DCs and innate lymphoid cells, although all major innate immune subsets are present in these mice (Niess and Adler, 2010, Sawa et al., 2010). Additionally, the absence of intestinal bacteria in GF mice may dramatically reduce the frequency of oncogenic mutations and tumor formation (Dove et al., 1997, Li et al., 2012). Fecal and bacteria transplantations have demonstrated that the gut microbiota can restore the number of immune cell populations in the gut immune system (Round and Mazmanian, 2009) and the gut’s sensitivity to tumor-inducing factors (Zhan et al., 2013). Recent studies have also shown that commensal bacterial composition can influence the type and robustness of the host’s immune responses (Ivanov and Honda, 2012). The immunomodulatory roles of several commensal bacterial species have been demonstrated (Ivanov and Honda, 2012). However, how these gut commensal microbes exert their effects on the gut immune response and inflammation is largely unknown.

LRRC19 belongs to the leucine-rich repeat (LRR) family and differs from other pathogen-recognizing receptors because it contains no cytoplasmic Toll/interleukin 1 (IL-1) receptor (TIR) domain. LRRC19 is highly expressed in gut epithelial cells under normal physiological conditions and is activated by multiple TLR ligands and *E. coli* (Chai et al., 2009, Su et al., 2014). Here we found that LRRC19 is involved in gut host-microbiota interactions and that it plays a critical role in promoting the recruitment of immune cells and intestinal inflammation.

## Results

### LRRC19 Deficiency Reduces Inflammatory Responses in Gut Tissues

**Figure 1:**
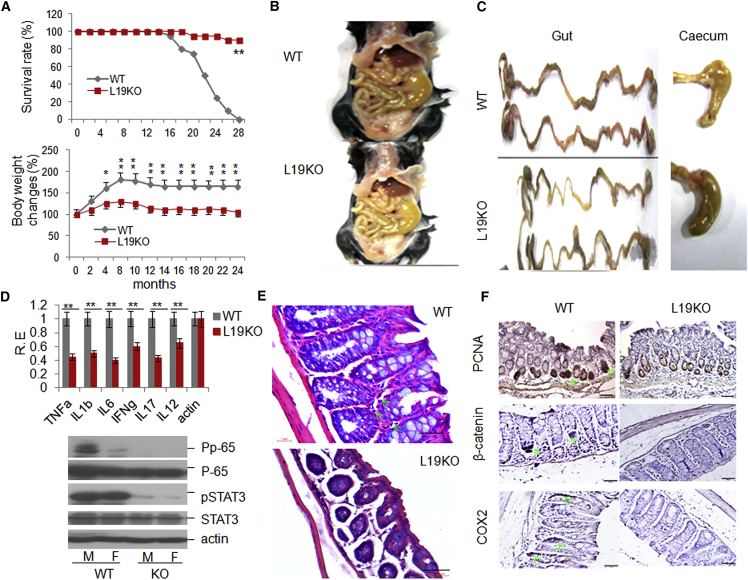
LRRC19 Deficiency Inhibits the Occurrence and Development of Gut Inflammation (A) Survival rates (top) and changes in body weight (bottom) of WT (WT, n = 24 [female, 12; male, 12]) and *Lrrc19* KO (L19KO, n = 24 [female, 12; male, 12]) mice on standard chow. (B and C) Morphology of gut and cecum from representative WT (B) and *Lrrc19* KO (C) mice after eating standard chow for 2 years. (D) qRT-PCR analyses (top) of TNF-α, IL-1β (IL1b), IL-6, IFNγ (IFNg), IL-17, and IL-12 and immunoblot (bottom) of pp-65 and pSTAT3 in colon tissues of WT (n = 6) and *Lrrc19* KO (n = 6) mice after standard chow for 2 years. RE, relative expression; M, male; F, female. (E) H&E staining of representative WT and *Lrrc19* KO mouse colons after standard chow for 2 years. The green arrow indicates inflammatory response cells. Scale bars, 40 μm. (F) Immunostaining of PCNA, β-catenin, and COX2 in colon tissues of representative WT and *Lrrc19* KO mice. The green arrows indicate PCNA, β-catenin, and COX2. Scale bars, 40 μm. ^∗^p < 0.05, ^∗∗^p < 0.01 (Wilcoxon’s test in A [top], ANOVA in A [bottom], t test in D; mean ± SD). See also Figure S1.

Our previous studies have suggested that gut epithelial cells express LRRC19 (Chai et al., 2009, Su et al., 2014). Here we used RT-PCR, in situ hybridization, and immunostaining to confirm these findings (Figures S1A–S1C). LRR family members such as TLRs, which are expressed in gut epithelial cells, play a significant role in enteritis, colitis, colon cancer, and metabolism-associated diseases (Rakoff-Nahoum et al., 2006, Vijay-Kumar et al., 2010). Because LRRC19 belongs to the LRR family, we first undertook a long-term observational study of *Lrrc19* knockout (KO) mice to determine the effects of LRRC19 on mouse health. *Lrrc19* KO mice displayed increased longevity compared with the cohoused wild-type (WT) littermates. Almost all *Lrrc19* KO (male and female) mice remained alive, whereas most of the WT mice (>80%) had died after 2 years on standard chow (Figure 1A). *Lrrc19* KO mouse body weights were lower than those of WT mice (Figure 1A). Interestingly, when the gut tissues of these mice were examined, almost all WT mice showed slight inflammation (low-grade enteritis and colitis) whereas *Lrrc19* KO mouse gut tissues did not. The gut tissues of *Lrrc19* KO mice were more yellow (Figure 1B) and the colon tissues were thinner in *Lrrc19* KO mice than in WT mice, and the ceca of *Lrrc19* KO mice were enlarged significantly (Figure 1C), as in GF mice (Aluwihare, 1971). These features of *Lrrc19* KO mice suggest a lack of immunological inflammatory responses in their gut tissues. Expression of cytokines such as tumor necrosis factor α (TNF-α), IL-1β, IL-6, interferon γ (IFNγ), IL-17, and IL-12 was lower in the gut tissues of *Lrrc19* KO mice than in those of WT mice over the long term (Figure 1D). Colitis-associated phospho-nuclear factor κB (NF-κB) p65, and -STAT3, which were detected readily in WT mice, were barely detectable in *Lrrc19* KO mice (Figure 1D). A histological examination of the LP showed a sparse stroma in *Lrrc19* KO mice, and the colonic sections from these mice showed no overt inflammatory infiltrate in the LP, which was observed in most WT mice (Figure 1E). Proliferating cell nuclear antigen (PCNA), a proliferative cell marker, was immunoreactive in numerous WT colon epithelial cells but not in *Lrrc19* KO mouse epithelial cells. β-Catenin and cyclooxygenase 2 (COX2), colon cancer-associated markers, were also detected more readily in WT mice compared with *Lrrc19* KO mice (Figure 1F). All of these data imply that LRRC19 is associated with enteritis, colitis, and colitis-associated tumorigenesis.

**Figure 2:**
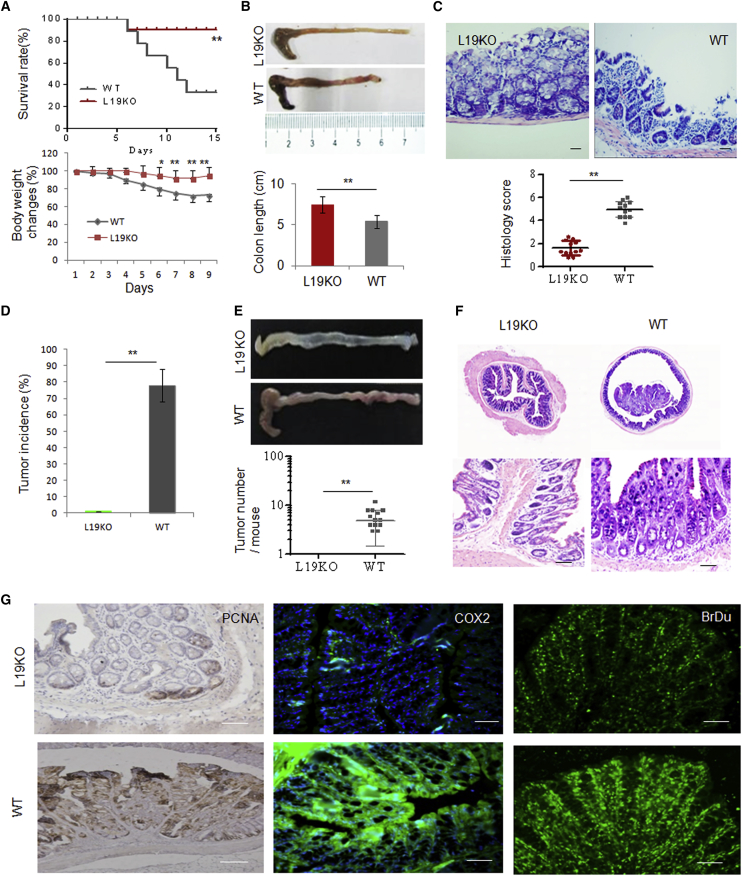
*Lrrc19* KO Mice Are Highly Resistant to DSS-Induced Colitis (A) Survival (top) and body weight (bottom) were monitored after the start of DSS. WT (n = 18, male) and *Lrrc19* KO (n = 18, male) mice were fed a 2% DSS solution in drinking water for 7 days and then switched to regular drinking water. (B) Length of WT and *Lrrc19* KO colon tissues. Mice were sacrificed on day 7 after the start of DSS, and colon length was measured. (C) H&E staining and histological scores of representative distal colon samples from WT and *Lrrc19* KO mice on day 7 after the start of DSS. Histological scores were assessed according to the methods described in the Supplemental Experimental Procedures. (D) Incidence of colon carcinoma in WT (n = 18, male) and *Lrrc19* KO (n = 18, male) mice after AOM-2% DSS treatment for 3 months. (E) Morphology and tumor numbers of colon carcinoma in WT and *Lrrc19* KO mice after AOM-2% DSS treatment for 3 months. (F) Histopathological changes in representative distal colon samples from *Lrrc19* KO and WT mice after staining with H&E. (G) Immunostaining of PCNA, COX2, and BrdU in colon tissues of representative WT and *Lrrc19* KO mice. Colon samples from *Lrrc19* KO and WT mice were stained by anti-PCNA or anti-COX2 antibodies. For the BrdU assay, mice were injected intraperitoneally with BrdU, and the colon sections were stained by anti-BrdU antibodies after 4 hr. Brown, PCNA; green, COX2 or BrdU. Scale bars, 40 μm. ^∗^p < 0.05, ^∗∗^p < 0.01 (Wilcoxon’s test in A [top], ANOVA in A [bottom], t test in B and D, mean ± SD; Mann-Whitney *U* test in C and E).

We next used dextran sodium sulfate (DSS)-induced colitis and an azoxymethane (AOM)-DSS-induced colon cancer model to examine the effects of LRRC19 on the occurrence and development of colitis and colon cancer. The results showed that LRRC19 deficiency conferred a marked resistance to DSS-mediated colitis. Although WT mice showed clear symptoms of colitis after exposure to 2.0% DSS in their drinking water for 7 days, the colitis symptoms, including survival rate, weight loss, colon shortening, and histology scores, were suppressed significantly in the cohoused *Lrrc19* KO mice (Figures 2A–2C). In the AOM-DSS-induced colon cancer model, WT mice developed a high incidence of colon tumors in the distal to middle colon, whereas no tumors were found in cohoused *Lrrc19* KO mice treated with the same protocol (Figures 2D–2F). The colon cancer-associated markers proliferating cell nuclear antigen (PCNA) and COX2 were also difficult to detect in *Lrrc19* KO mice (Figure 2G). 5-Bromo-2-deoxyuridine (BrdU) experiments showed strong absorbance by colon epithelial cells of WT mice but not by those of *Lrrc19* KO mice (Figure 2G). These results indicated that LRRC19 functions in gut-related tumorigenesis. LRRC19 is difficult to detect in hematopoietic cells, but its expression is induced in inflammatory environments (unpublished data). Therefore, we assessed intrinsic hematopoietic cell functions in colitis generation and development. WT/WT chimeras (WT bone marrow [BM] donor cells transplanted into lethally irradiated WT recipients), but not cohoused KO/ko chimeras (*Lrrc19* KO BM donor cells transplanted into lethally irradiated *Lrrc19* KO recipients), responded effectively to DSS-induced colitis, further confirming the role of LRRC19 in colitis (data not shown). Notably, in *Lrrc19* KO mice that received WT bone marrow, the incidence and severity of colitis were significantly lower than those of WT mice transplanted with *Lrrc19* KO BM (Figures S1D–S1F), suggesting that epithelial but not hematopoietic expression of LRCC19 is necessary for the proinflammatory role of LRRC19 in DSS colitis. Together, these data indicate that LRRC19 deficiency impairs the inflammatory responses in gut tissues.

### LRRC19 Deficiency Reduces the Recruitment of Immune Cells

**Figure 3:**
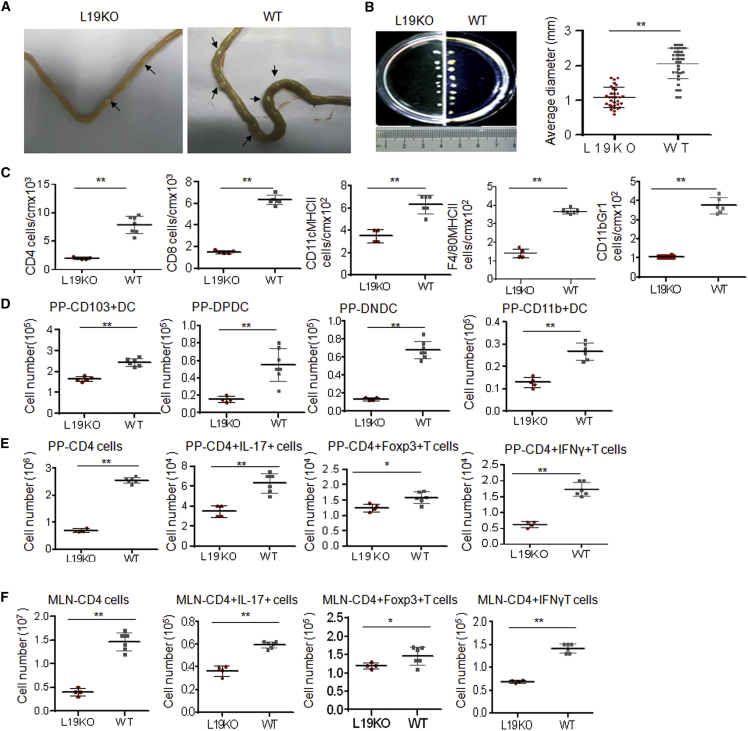
LRRC19 Deficiency Affects Maturation of Gut-Associated Lymphoid Tissues (A and B) Visible PP number (A) and sizes (B) in the guts of WT and *Lrrc19* KO mice. (C) Absolute numbers of CD4^+^T cells, CD8^+^ T cells, CD11c^+^MHCII^+^ cells, F4/80^+^MHCII^+^ cells, and CD11b^+^Gr1^+^ cells in colon tissues. The absolute numbers were standardized by calculating the numbers per l cm of colon. (D) Numbers of CD11c^+^CD103^+^CD11b^−^DCs (pp-CD103^+^DC), CD11C^+^CD103^+^CD11b^+^ DCs (PP-DP-DC), CD11C^+^CD11b^−^CD103^−^DCs (PP-DNDC), and CD11C^+^CD11b^+^ (PP-CD11b^+^DC) in PPs as assessed by flow cytometry analysis. (E and F) Number of CD4^+^, CD8^+^, CD4^+^Foxp3^+^, CD4^+^IL-17^+^, and CD4^+^IFNγ^+^ T cells in PPs (E) and MLNs (F). Cell numbers in age- and sex-matched WT (n = 6) and *Lrrc19* KO (n = 6) mice were compared. ^∗^p < 0.05, ^∗∗^p < 0.01 (Mann-Whitney *U* test). The data are representative of three independent experiments. See also Figures S2 and S3.

We next investigated the factor(s) that cause(s) the decreased inflammation in *Lrrc19* KO gut tissues. *Lrrc19* KO gut tissues and gut-associated lymphoid tissues from mice previously subjected to long-term observation were examined again. We found that *Lrrc19* KO mice had fewer and smaller gut tissue-associated lymph node PPs compared with cohoused WT mice (Figures 3A and 3B), indicating that fewer immune cells had accumulated in their gut immune systems. Because immune cells such as DCs, adaptive lymphocytes (including Th1 and Th17 cells), and immunoregulatory cells such as regulatory T (Treg) cells play critical roles in colitis and colitis-associated cancer (Nguyen et al., 2015), we examined the composition and absolute numbers of immune cells in the LPs, PPs, and MLNs of WT and *Lrrc19* KO mice. We found that the absolute numbers of CD4^+^ T cells, CD8^+^ T cells, DCs (CD11c^+^MHCII^+^ cells), macrophages (F4/80^+^MHCII^+^ cells), and immature myeloid cells (CD11b^+^Gr1^+^ cells) were much lower in the LPs of *Lrrc19* KO mice than in those of WT mice (Figure 3C). The smaller numbers of innate and adaptive immune cells in *Lrrc19* KO mice were confirmed with immunostaining (Figure S2). The absolute numbers of CD103^+^CD11b^–^ (CD103^+^ DCs), CD103^+^CD11b^+^ (double-positive [DP] DCs), and CD103^–^CD11b^+^ (CD11b^+^ DCs) subsets were also lower in the PPs of *Lrrc19* KO mice compared with those of cohoused WT mice (Figure 3D). However, the proportion of the CD11c^+^CD103^+^ DC subpopulation, which plays a critical role in maintaining gut tolerance (Benson et al., 2007, Coombes et al., 2007), did not decrease but, rather, increased in the PPs and MLNs of *Lrrc19* KO mice (Figure S3B). The proportion of CD11b^+^ DCs (CD11C^+^CD103^+^CD11b^+^ and CD11c^+^CD11b^+^ cells) was lower in *Lrrc19* KO mice than in WT mice (Figure S3B), indicating that there were fewer hematopoietic CD11b^+^ DCs in *Lrrc19* KO mice than in WT mice. Importantly, the absolute numbers of adaptive lymphocytic subsets (Th1 and Th17 cells) in the PPs and MLNs of *Lrrc19* KO mice, which may promote inflammation responses, were less compared with those of WT mice (Figures 3E and 3F). Th17 cell frequency in the PPs and MLNs was also reduced markedly in *Lrrc19* KO mice, whereas the Treg (Foxp3^+^) cell proportion, which may suppress inflammation responses, was increased (Figure S3D). Notably, the reduced number of T cells and the reduction in the Th17 subpopulation in *Lrrc19* KO mice were limited to the gut tissue. *Lrrc19* KO and WT mice did not differ in the numbers of DCs or T cells in their spleens. No similar phenomena were found in *Tlr4* KO, *Tlr2* KO, or *Myd88* KO mice (data not shown). These data suggest that the decreased number of immune cells and their subsets may be responsible for the reduced gut inflammation in *Lrrc19* KO mice.

### Reduced Chemokine Levels in *Lrrc19* KO Gut Tissues Are Responsible for Fewer Gut Immune Cells

**Figure 4:**
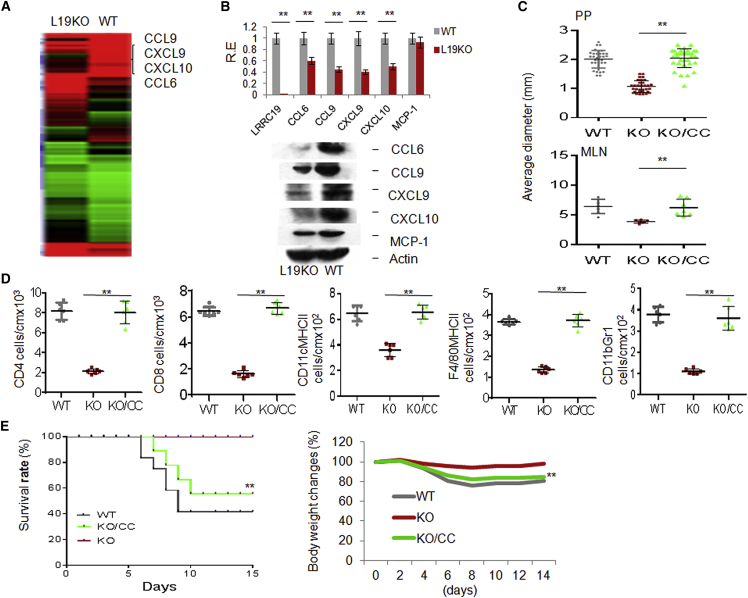
LRRC19 Deficiency Reduces the Expression of Chemokines CCL6, CCL9, CXCL9, and CXCL10 (A) Microarray analyses of gene expression in WT and *Lrrc19* KO mice given standard chow. (B) qRT-PCR (top) and immunoblot (bottom) of CCL6, CCL9, CXCL9, and CXCL10 in WT and *Lrrc19* KO colon epithelial cells. (C) Size of visible PP and MLN in *L19* KO mice with (KO/CC) or without (KO) CCL6-, CCL9-, CXCL9-, and CXCL10-expressing adenovirus injection. (D) Absolute number of CD4^+^ and CD8^+^ T cells, CD11C^+^MHCII^+^ DCs, F4/80MHCII, and CD11b^+^Gr1^+^ cells in colon tissue of *L19* KO mice with or without (control adenovirus only) chemokine adenovirus injection as assessed by flow cytometric analysis. CC, CCL6-, CCL9-, CXCL9-, and CXCL10-expressing adenovirus complexes; WT, cell numbers from the colon tissues of WT mice. (E) Survival (right) and body weight (left) were monitored until day 14 after the start of DSS. *L19 KO* mice with (n = 18) or without (n = 18) CCL6, CCL9, CXCL9, and CXCL10 adenovirus injection were fed a 2% DSS solution in drinking water for 7 days and then switched to regular drinking water. ^∗^p < 0.05, ^∗∗^p < 0.01 (t test in B, mean ± SD; Mann-Whitney *U* test in C and D; Wilcoxon’s test in E (right); ANOVA in E (left). See also Figure S4.

We next investigated the cause of the decreased number of immune cells in *Lrrc19* KO gut tissues. Previous studies have shown that chemokines play a critical role in the recruitment and retention of immune cells. However, for chemokines such as MCP-1/CCL2, which plays roles in recruiting immune cells to gut tissues (Popivanova et al., 2009), there was no remarkable difference in CCL2 expression between cohoused WT and *Lrrc19* KO mice, especially in the levels of transcription (Figure 4B). To identify chemokines involved in recruiting immune cells in gut tissues, we used a microarray to analyze the differential expression of genes in the gut epithelial cells of cohoused WT and *Lrrc19* KO mice. The expression of several chemokines, including CCL6, CCL9, CXCL9, and CXCL10, was reduced significantly in *Lrrc19* KO mice (Figure 4A; http://www.ncbi.nlm.nih.gov/geo/query/acc.cgi?acc=GSE62487). The reduced levels of chemokine expression in LRRC19-deficient epithelial cells were confirmed further with qRT-PCR, immunoblotting, and immunostaining (Figure 4B; data not shown). These chemokines play a critical role in recruiting innate and adaptive immune cells, including DCs, macrophages, and CD4^+^ and CD8^+^ T cells (Asensio et al., 1999, Coelho et al., 2007, Harris et al., 2012, Núñez et al., 2010, Zhao et al., 2003). Indeed, administration of CCL6-, CCL9-, CXCL9-, and CXCL10-expressing adenoviral complexes could not only rescue the innate and adaptive immune system in *Lrrc19* KO mice (Figures 4C–4E) but also promoted the accumulation of innate and adaptive immune cells, including CD4^+^ and CD8^+^ T cells, DCs, macrophages, and immature myeloid cells in the LP (Figure S4B) in WT mice. The MLN sizes were also significantly larger in mice injected with chemokine-expressing adenoviral complexes (Figure S4C). These extra chemokines also increased the sensitivity to DSS-mediated colitis in *Lrrc19* KO mice (Figure 4E) and in WT mice (Figures S4D and S4E). These data, together, suggest that LRRC19-associated CCL6, CCL9, CXCL9, and CXCL10 in gut epithelial cells play a critical role in the recruitment of gut immune cells.

### Altered Gut Microbiota in *Lrrc19* KO Mice Affects Chemokine Expression

**Figure 5:**
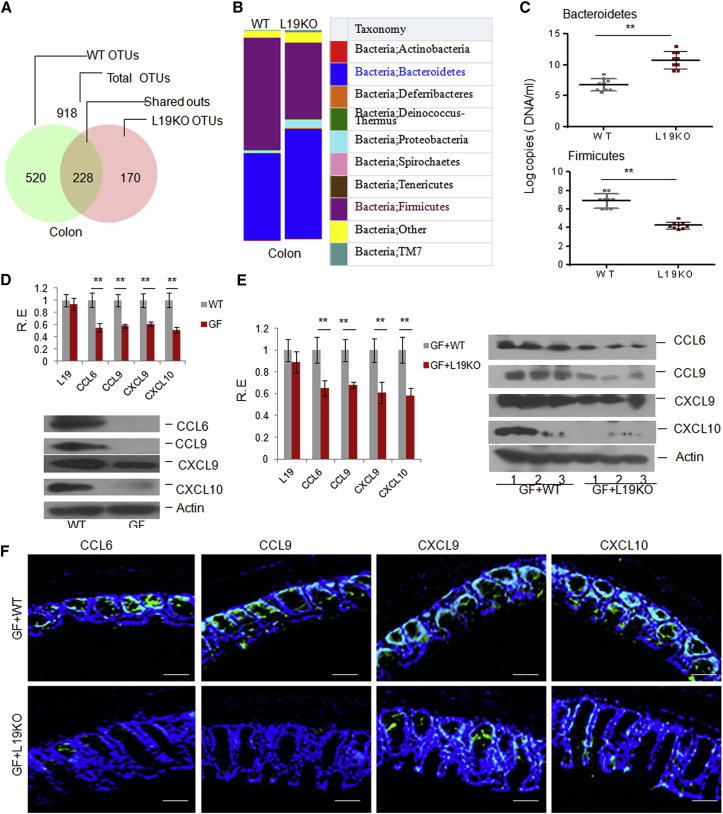
The LRRC19-Associated Gut Microbiota Modulates the Expression of Chemokines (A and B) 16S rRNA analyses of gut microbiota of WT and *Lrrc19* KO mice. The samples were clustered at the operational taxonomic unit (OTU) (A) and phylum (B) levels using the sample OTU and sample phylum count matrices, respectively. (C) qRT-PCR of gut microbiota. The abundance of bacteria in WT and *Lrrc19* KO mice was measured as bacterium-specific 16S rRNA copy numbers by qPCR analysis of fecal pellets. Standard curves were prepared from serial dilution of *E. coli* genomic 16S rRNA extracted in the same manner as above. (D) qRT-PCR and immunoblot of CCL6, CCL9, CXCL9, and CXCL10 in WT and GF mice. (E) qRT-PCR and immunoblot of CCL6, CCL9, CXCL9, and CXCL10 in GF+WT and GF+L19KO mice. GF+WT, GF mice transplanted with the microbiota from the feces of WT mice; GF+L19KO, GF mice transplanted with microbiota from the feces of *Lrrc19* KO mice. (F) Immunostaining of CCL6, CCL9, CXCL9, and CXCL10 in GF+WT and GF+L19KO mice. Scale bars, 40 μm. ^∗^p < 0.05, ^∗∗^p < 0.01 (Mann-Whitney *U* test in C; t test in D and E, mean ± SD). See also Table S1.

We next addressed factor(s) that potentially affect chemokine expression in gut tissues of *Lrrc19* KO mice. Previous studies have suggested that gut commensal microbiota composition is important for establishment of the intestinal immune system. The fewer immune cells in the *Lrrc19* KO mice may, therefore, be attributable to their altered gut microbiota. We first analyzed fecal microbiotas by pyrosequencing 16S rRNAs in WT and *Lrrc19* KO mice. The animal husbandry, parental genotypes, and environmental influences were controlled carefully. The *Lrrc19* KO and cohoused WT mice shared the same bacterial phyla, but, among their microbiotas, the proportions of Firmicutes and Bacteroidetes were significantly different between *Lrrc19* KO (29% Firmicutes and 55% Bacteroidetes) and WT mice (59% Firmicutes and 34% Bacteroidetes). However, there was a marked increase in Bacteroidetes and a marked reduction in Firmicutes in *Lrrc19* KO mice compared with WT mice (Figures 5A and 5B; Table S1). These data were confirmed with 16S rRNA qPCR (Figure 5C). The relative abundances of bacterial phyla in WT mice (Figures 5A and 5B; Table S1) were consistent with the data of other studies (Ley et al., 2006, Turnbaugh et al., 2009). Further analyses showed far fewer *Clostridium* bacteria in the colons or *Lactobacillus* bacteria in the intestines of *Lrrc19* KO compared with WT mice (Table S1). Therefore, we next used a GF mouse model to demonstrate that the altered gut microbiota in *Lrrc19* KO mice is a factor in their reduced levels of chemokines. We first compared the chemokine levels in the intestinal tissues of *WT* and GF mice. The expression of CCL6, CCL9, CXCL9, and CXCL10 was much lower, or even barely detectable, in the intestinal tissues of GF mice compared with WT mice (Figure 5D). However, the expression of these chemokines was remarkably higher in GF mice transplanted with WT mouse feces compared with that in GF mice transplanted with *Lrrc19* KO mouse feces (Figures 5E and 5F), suggesting that the altered gut microbiota in *Lrrc19* KO mice affects chemokine expression in their gut epithelial cells.

### REGs Are Involved in the Alteration of the Gut Microbiota in *Lrrc19* KO Mice

**Figure 6:**
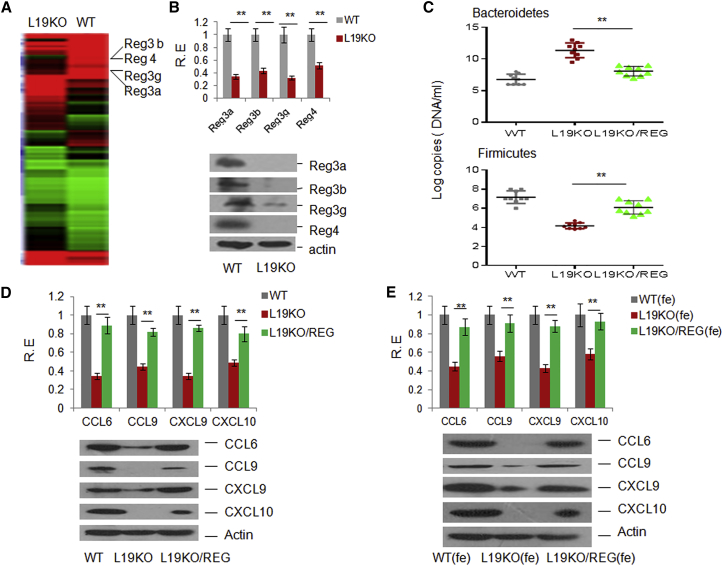
Expression of Chemokines Is Associated with the REG-Mediated Microbiota (A) Microarray analysis of gene expression in gut tissues of WT and *Lrrc19* KO mice. (B) qRT-PCR (top) and immunoblot (bottom) of REG3α, REG3β, REG3γ, and REG4 in WT and *Lrrc19* KO colon tissues. (C) qRT-PCR of gut microbiotas. The abundance of bacteria in *L19* KO mice with (KO/REGs) or without (KO, control adenoviruses only) REG3α-, REG3β-, REG3γ-, and REG4-expressing adenovirus injection was measured as 16S rRNA copy numbers by qPCR analysis of fecal pellets. Standard curves were prepared from serial dilution of *E. coli* genomic 16S rRNA extracted in the same manner as above. (D) qRT-PCR and immunoblot of CCL6, CCL9, CXCL9, and CXCL10 in *L19* KO mice with or without (control adenoviruses only) REG adenovirus injection. Adenovirus-Reg3α, -Reg3β, -Reg3γ, and -Reg4 complexes (REG) or control adenovirus were injected intraperitoneally once per week, three times. (E) qRT-PCR and immunoblot of CCL6, CCL9, CXCL9, and CXCL10 in feces-transplanted mice. Mice were first treated using pan-antibiotics (1 g/l ampicillin, Sigma), 0.5 g/l vancomycin, 1 g/l neomycin sulfate, and 1 g/l metronidazole) in drinking water for 4 weeks and were then transplanted with microbiotas from feces. WT(fe), *Lrrc19* KO mice transplanted with the microbiota from the feces of WT mice; L19KO(fe), *Lrrc19* KO mice transplanted with the microbiota from the feces of *Lrrc19* KO mice with control adenovirus injection; L19KO/REG(fe), *Lrrc19* KO mice transplanted with the microbiota from the feces of REG adenovirus-administered *Lrrc19* KO mice. Expression of CCL6, CCL9, CXCL9, and CXCL10 was analyzed 3 days after transplantation. ^∗^p < 0.05, ^∗∗^p < 0.01 (Mann-Whitney *U* test in C; t test in D and E, mean ± SD). See also Figures S5, S6, and S7.

We next addressed the causes of the altered gut microbiota in *Lrrc19* KO mice. To investigate this, we again analyzed the microarray data from gut epithelial cells of WT and *Lrrc19* KO mice. The expression of REGs, including REG3α, REG3β, REG3γ, and REG4, was significantly lower in the gut epithelial cells of *Lrrc19* KO mice compared with the gut epithelial cells of WT mice (Figure 6A; http://www.ncbi.nlm.nih.gov/geo/query/acc.cgi?acc=GSE62487). These results were confirmed with qRT-PCR, immunoblotting, and immunostaining (Figure 6B; data not shown). Because the members of the REG family have the capacity to specifically kill Gram-positive bacteria (Brandl et al., 2007, Mukherjee et al., 2014, Vaishnava et al., 2011) and alter the composition of the gut microbiota (Figure 6C), the reduced expression of REGs in LRRC19 KO mice may explain their altered gut microbiota.

A critical question is whether the reduced expression of CCL6, CCL9, CXCL9, and CXCL10 is associated with REG-mediated gut microbiotas in *Lrrc19* KO mice. To demonstrate this, we first administrated REG adenoviruses into *Lrrc19* KO mice. We found that the expression of chemokines was much higher in the intestinal tissues of *Lrrc19* KO mice with REG adenovirus administration compared with uninjected mice (Figure 6D), indicating that the expression of chemokines is related to REG levels in gut tissues. We next addressed whether REG-mediated chemokines were associated with the gut microbiota of mice administrated REG adenoviruses. When gut microbiotas of *Lrrc19* KO mice with REG adenoviruses were transplanted into *Lrrc19* KO mice, the expression of chemokines was much higher than in *Lrrc19* KO mice transplanted with control feces (Figure 6E), indicating that the altered gut microbiota after administration of REG adenoviruses affects chemokine expression in their gut epithelial cells. Taken together, these results suggest that the REG-mediated gut commensal microbiota modulates the expression of chemokines.

### Gut *Lactobacillus* Directly Regulates the Expression of REGs through LRRC19-Mediated Activation of NF-κB

Because reduced REGs are responsible for the altered microbiota, which caused the decreased chemokine levels in *Lrrc19* KO mice, we next explored how LRRC19 affects the expression of REGs. LRRC19, as a potential bacterium recognition receptor, may be activated by gut bacteria to regulate the expression of REGs. The genus *Lactobacillus* was decreased remarkably in Lrrc19 KO mice (Table S1). Therefore, the reduced expression of the REGs in *Lrrc19* KO mice may be caused by the decrease in *Lactobacillus*. After screening the isolated gut commensal bacteria, we identified the strain of *Lactobacillus* with the highest similarity to *Lactobacillus taiwanensis* strain BCRC 17755, *Lactobacillus* NK6 (colony 6) (Figure S5). This strain could induce the expression of REGs in gut epithelial cells (Figures S6A and S6B). Notably, lactobacilli could activate the intracellular NF-κB signaling pathway in LRRC19- but not ΔLRRC19-transfected (absence of an extracellular region) HEK293T cells (Figure S6C). All of these data suggest that LRRC19 may directly regulate the expression of REGs.

We next dissected the molecular pathways by which LRRC19 signaling regulates the expression of REG family proteins. LRRC19 deficiency affected the phosphorylation of p65, p38, and c-Jun N-terminal kinase (JNK) in response to *Lactobacillus* (Figure S7A), indicating that LRRC19 activates multiple signaling pathways. Consistent with previous findings (Su et al., 2014), there was more K63-linked ubiquitin on the TRAF6 from WT gut epithelial cells than on TRAF6 from *Lrrc19* KO cells (Figure S7B). The ubiquitination of TRAF2 was also inhibited markedly in WT gut epithelial cells, whereas the ubiquitination of TRAF2 in *Lrrc19* KO cells was increased greatly in response to *Lactobacillus* NK6 (Figure S7C). Because TRAF2 and TRAF6 are critical adaptor molecules of NF-κB signaling pathways, we also examined the effect of NF-κB deficiency on the expression of REG family members. The expression of REG family members such as REG3α, REG3β, REG3γ, and REG4 was lower in *NF-κB* KO mice than in WT mice (Figure S7D). *NF-κB* KO mouse gut epithelial cells had a highly reduced response to *Lactobacillus* NK6 (Figure S7E), implying that LRRC19 mediates the expression of REG3 family members through NF-κB signaling pathways. This is consistent with previous findings (Vaishnava et al., 2011). These results suggest that LRRC19 mediates the expression of REG proteins through the TRAF2- and TRAF6-mediated NF-κB signaling pathways.

### Exogenous LRRC19 Promotes the Occurrence and Development of Colitis

**Figure 7:**
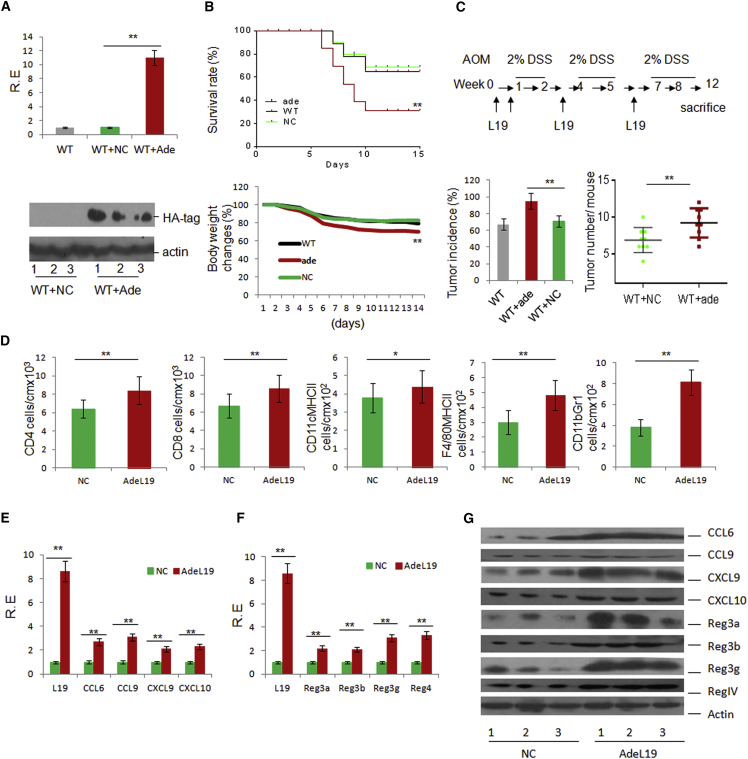
Exogenous LRRC19 Promotes the Occurrence and Development of Gut Inflammation (A) qRT-PCR (top) and immunoblot (bottom) of LRRC19 in LRRC19 adenovirus- (WT+ade) or control adenovirus (WT+NC)-injected mice. Colon tissues were lysed and analyzed for LRRC19 expression by anti-hemagglutinin (HA), with which LRRC19 was tagged in adenoviruses. (B) Survival (top) and body weight (bottom) were monitored until day 14 after the start of DSS. Mice with (Ade) and without LRRC19 (NC) adenovirus injection (n = 16, male) were fed a 2% DSS solution in drinking water for 7 days and then switched to regular drinking water. (C) Experimental design (top) and incidence and tumor numbers (bottom) of colon carcinoma in LRRC19 adenovirus- (WT+ade) (n = 16) or control adenovirus-administered (WT+NC) mice (n = 16) after AOM-2% DSS treatment for 3 months. (D) Absolute number of CD4^+^T cells, CD8^+^ T cells, CD11C^+^MHCII^+^ DCs, F4/80^+^MHCII^+^, and CD11b^+^Gr1^+^ cells in colon tissues of LRRC19 adenovirus- (AdeL19) or control adenovirus (NC)-injected mice (n = 6) as assessed by flow cytometry. The absolute numbers were standardized by calculating the numbers per l cm of colon. (E, F, and G) qRT-PCR (E and F) and immunoblot (G) of CCL6, CCL9, CXCL9, and CXCL10 and REG3α, REG3β, REG3γ, and REG4 in LRRC19 adenovirus (AdeL19)- or control adenovirus (NC)-injected mice (n = 6). Lanes 1–3 in (G) are representatives of six mice. ^∗^p < 0.05, ^∗∗^p < 0.01 (Wilcoxon’s test in B [top]; ANOVA in B [bottom]; t test in A, C, D, E, and F, mean ± SD; Mann-Whitney *U* test in C for tumor number).

Finally, we examined the importance of LRRC19 in promoting the recruitment of immune cells and intestinal inflammation. LRRC19-expressing adenovirus was injected intraperitoneally three times per week, with demonstrable infection (Figure 7A). This treatment increased the sensitivity of mice to DSS-mediated colitis (Figure 7B) and AOM-DSS-induced colon cancer (Figure 7C). The administration of LRRC19-expressing adenovirus also increased the infiltration of different immune cells into the gut tissues (Figure 7D) and altered the immune cell proportions in that the CD103^+^CD11b^+^ cell population in the LP increased dramatically in mice administered LRRC19-expressing adenovirus and the Th1^+^ (IFNγ^+^) and Th17^+^ cell subsets in the PPs and MLNs also increased (data not shown). qRT-PCR and immunoblot analyses showed that CCL6, CCL9, CXCL9, and CXCL10 expression in mice administered LRRC19-expressing adenovirus was also higher than in control mice (Figures 7E and 7G). Expression of the REG family proteins REG3α, REG3β, REG3γ, and REG4 was higher in adenovirus-treated mice than in mice injected with the control vector (Figures 7F and 7G). All of these data confirm the critical role of LRRC19 in the recruitment of immune cells and intestinal inflammation.

## Discussion

Gut epithelial cells express multiple pattern recognition receptors, such as TLRs and Nod-like receptors, that play important roles in eliminating pathogenic microorganisms. However, the expression levels of these TLRs are too low under normal physiological conditions to produce a responsive to TLR stimuli (Melmed et al., 2003). Therefore, it is not really clear what type of receptors plays a critical role in the establishment and maturation of the gut immune system. We found that LRRC19, which does not contain a cytoplasmic Toll/interleukin 1 receptor domain, determines the recruitment of immune cells and intestinal inflammation under normal physiological conditions. Unlike the pathogen-recognizing receptors, LRRC19 is highly expressed on gut epithelial cells and directly mediates the TRAF2- and TRAF6 NF-κB signaling pathways in gut epithelial cells.

Mining the microbiota for bacterial strains that are responsible for shaping the gut immune system is a formidable combinatorial problem. Although some progress has been made in identifying gut microbe species that preferentially stimulate a specific program of immune maturation, the gut-specific commensal microbiota for maintaining the maturation of the whole gut immune system is largely unknown. We found that gut commensal microbes of the genus *Lactobacillus* are involved in this process. Although the particular bacterial products are unknown, we demonstrated that *Lactobacillus* may promote the expression of REG3α, REG3β, and REG3γ through LRRC19-mediated TRAF2- and TRAF6 NF-κB signaling pathways in gut epithelial cells. This may affect the recruitment of immune cells and intestinal inflammation by gut microbiota-mediated chemokines. *Lactobacillus* are Gram-positive bacteria with the cell wall component peptidoglycan. Recognition of peptidoglycan is important in initiating and shaping the immune response under both homeostatic and infection conditions (Sorbara and Philpott, 2011). Indeed, four other secreted peptidoglycan recognition proteins, PGLYRP 1-4, as well as two intracellular sensors of peptidoglycan, Nod1 and Nod2, also have important roles in shaping mammalian immune responses (Sorbara and Philpott, 2011).

Our study establishes an LRRC19-based regulatory axis that may promote the recruitment of immune cells and intestinal inflammation. Chemokines CCL6, CCL9, CXCL9, and CXCL10 may directly recruit immune cells into the gut tissues, whereas REG family proteins, including REG3α, REG3β, REG3γ, and REG4, play critical roles by affecting the composition of the gut microbiota, which may modulate chemokine expression. Commensal microbes of the genus *Lactobacillus* are essential for the establishment of the gut immune system by activating the LRRC19-mediated signaling pathway, which may induce the expression of REG family proteins such as REG3α, REG3β, and REG3γ. These results represent an important advance in understanding how gut commensal microbes exert their effects to promote the recruitment of immune cells and intestinal inflammation through the microbe receptors expressed on gut epithelial cells. This will be invaluable when designing therapeutic strategies for colitis and colitis-associated diseases such as colon carcinoma.

## Experimental Procedures

### Mice

Four-to six-week-old male or female C57BL/6 and GFP transgenic C57BL/6 mice were obtained from the Beijing Animal Center. *Lrrc19* KO mice were generated by us (Su et al., 2014). *Tlr2* KO mice were obtained from the Nanjing Animal Center. *Nf-κb* KO (p50^−/−^) mice were provided by Prof. Zhexiong Lian (University of Science and Technology of China). Germ-free mice were generated by the Third Military Medical University and were bred and maintained in sterile Trexler-type isolators. All procedures were conducted according to the Institutional Animal Care and Use Committee of the Model Animal Research Center. Animal experiments were approved by the Animal Ethics Committee of Nankai University.

### Preparation of Specific Experimental Mice

Chimeric mice were generated according to our previously published method (Su et al., 2014). Briefly, 6-week-old *Lrrc19* KO and WT GFP transgenic C57BL/6 mice were lethally irradiated with 9 Gy of total body irradiation. BM cells were obtained from the femora of donor mice and collected in RPMI 1640 medium containing 100 U/ml penicillin/streptomycin. Irradiated recipient mice were injected with 200 μl of the appropriate cell suspension via the tail vein. The recipient mice were maintained in a sterile facility for 8 weeks to allow for complete engraftment with donor bone marrow. To assay bone marrow reconstitution, spleens were harvested from chimeric mice, and single-cell suspensions of splenocytes were prepared in PBS and then analyzed by flow cytometry to detect GFP+ spleen cells and to determine the donor/recipient chimerism of the hematopoietic compartment.

For microbiota transplantation mice, cecal contents were pooled from five mice. Cecal contents (150 mg) were collected in an anaerobic chamber, suspended in 1 ml PBS, and immediately administered intragastrically (0.1 ml/mouse) to sterilely packed, 6- to 7-week-old germ-free mice or pan-antibiotic-treated mice. The *Lactobacillus* NK colony was selected and cultured in *Lactobacillus*-selective medium, suspended in PBS, and immediately administered intragastrically (1 × 10^9^ colony-forming units [CFUs]/mouse) to sterilely packed, 6- to7-week-old mice three times per week for 4 weeks.

For mice expressing exogenous LRRC19; chemokine or REG; adenovirus-LRRC19, -CCL6, -CCL9, -CXCL9, and -CXCL10 complexes (CC); or adenovirus-Reg3α, -Reg3β, -Reg3γ, and –Reg4 complexes (REG) were injected intraperitoneally at the indicated time. The expression of LRRC19, CCL6, CCL9, CXCL9, and CXCL10 or Reg3α, Reg3β, Reg3γ, and Reg4 in gut epithelial cells was determined using qRT-PCR and immunoblotting.

### Isolation of the Gut *Lactobacillus* Strain *Lactobacillus* NK6

For isolation of the gut microbiota, the cecal contents from *WT* mice were serially diluted with PBS and seeded onto *Lactobacillus*-selective culture plates. After culture under aerobic conditions or strictly anaerobic conditions at 37°C for 24–48 hr, individual colonies were picked up and cultured for an additional 1–2 days at 37°C in *Lactobacillus*-selective medium (*Lactobacillus* MRS medium) (per liter: protease peptone, 10.0 g; beef extract, 10.0 g; yeast extract, 5.0 g; Tween 80, 1.0 ml; ammonium citrate, 2.0 g; sodium acetate, 5.0 g; magnesium sulfate, 0.1 g; manganese sulfate, 0.05 g; di-potassium phosphate, 2.0 g; and glucose at 2% [w/v]). The isolated colonies were collected into stock medium (10% glycerol) and stored at −80°C. The sequences of the 16S rRNAs of the isolated colonies were obtained by cycle sequencing and were then aligned with the 16S rRNA database of GenBank using BLAST. Each inquiry gave the 100 most similar sequence results, including different bacterial genera. For each genus, one bacterial strain with the highest Max Score was selected, and its sequence was downloaded. Next, all obtained sequences were aligned by MUSLE, and then the neighbor-joining method was used to construct a phylogenetic tree.

### Flow Cytometry

Single-cell suspensions of MLNs, PPs, and spleen were prepared by mashing them in a cell strainer (70 mm), stained, and analyzed by flow cytometry. For the staining of lamina propria lymphocytes, gut epithelial cells were first removed using 1 mM EDTA and then digested in RPMI medium with 5% fetal bovine serum (FBS) and 0.15% collagenase II (275 U/mg)/0.05% dispase (1.1 U/mg) (Invitrogen) for 1 hr at 37°C. LP cells were filtered to minimize mucus contamination, stained, and analyzed by flow cytometry. Dead cells were eliminated through PI staining.

For intracellular staining, cells were cultured and stimulated for 16 hr with 50 ng/ml phorbol 12-myristate 13-acetate (PMA, Sigma) and 1 μg/ml ionomycin (Sigma) in the presence of GolgiStop (10 ng/ml, BD Biosciences). After incubation for 16 hr, cells were washed in PBS, and surface CD4 was stained with a fluorescein isothiocyanate (FITC)- or phycoerythrin (PE)-labeled anti-CD4 antibody. Cells were then washed, fixed in Cytofix/Cytoperm, permeabilized with perm/wash buffer (BD Biosciences), and stained with PE- or FITC-labeled anti-IFNγ, anti-Th17, or anti-Foxp3. Meanwhile, dead cells were eliminated through PI staining.

### In Vitro Stimulation

For in vitro stimulation, gut epithelial crypts were stimulated for 12 hr using bacteria (crypt cells: bacteria = 1:100) and then lysed for immunoblot analyses. To isolate crypts, samples were transferred to 5 mM EDTA in PBS (pH 8), shaken by hand for 1 min, incubated at 4°C for 15 min, and passaged through 70-μm filters (BD Falcon) to collect the flowthrough. The fraction containing intact and isolated crypts was collected by centrifugation at 75 × *g* for 5 min at 4°C and washed once with PBS. In some cases, bacteria (1 × 10^7^) were injected directly into colon segments to stimulate colon epithelial cells, and then colon epithelial cells were isolated using 5 mM EDTA.

### RT-PCR and qRT-PCR

RT-PCR and qRT-PCR were performed according to methods published previously (Su et al., 2014). Total RNA was extracted from the cells, tissues, and organs using TRIzol reagent (Invitrogen). First-strand cDNA was generated from total RNA using oligo-dT primers and reverse transcriptase (Invitrogen). The PCR products were visualized on 1.0% (WT/v) agarose gels. qRT-PCR was conducted using QuantiTect SYBR Green PCR Master Mix (QIAGEN) and specific primers in an ABI Prism 7000 analyzer (Applied Biosystems). GAPDH mRNA expression was detected in each experimental sample as an endogenous control. All reactions were run in triplicate.

### Immunoprecipitation and Immunoblot Analysis

Immunoprecipitation and immunoblot analysis were performed according to methods published previously (Su et al., 2014). The cells were lysed with cell lysis buffer (Cell Signaling Technology) supplemented with a protease inhibitor “cocktail” (Calbiochem). Immunoprecipitation (IP) was performed essentially as described by Thermo Scientific. For the immunoblot, hybridizations with primary antibodies were conducted for 1 hr at room temperature in blocking buffer. The protein-antibody complexes were detected using peroxidase-conjugated secondary antibodies (Boehringer Mannheim) and enhanced chemiluminescence (Amersham).

### Statistical Analysis

Student’s t test, one-way ANOVA, Bonferroni’s multiple comparison test, Wilcoxon’s test, and Mann-Whitney *U* test were used to determine significance. A 95% confidence interval was considered significant and was defined as p < 0.05 (^∗^p < 0.05, ^∗∗^p < 0.01, ^∗∗∗^p < 0.001).

## Author Contributions

R.Y. designed the research and wrote the paper. S.C., X.S., B.Z., H. Yan, Y.H., A.W., H. Yun, and Y.Z. conducted the in vivo experiments and immunoassays. X.S, S.C., Y.H., H. Yan, E.W., and H. Yun carried out the in vitro assays. Y.C., W.L., and H.W. provided assistance for the animal experiments. Y.L. and F.L. reviewed the manuscript.

## Accession Numbers

The accession number for the gene array data reported in this paper is GEO: GSE62487.

## Supplemental Information

Document S1. Supplemental Experimental Procedures, Figures S1–S7, and Table S1

Document S2. Article plus Supplemental Information
