# Supplementary material for: The Gut Epithelial Receptor LRRC19 Promotes the Recruitment of Immune Cells and Gut Inflammation
Source: Cell Rep. 2016 Jan 14;14(4):695–707. doi: 10.1016/j.celrep.2015.12.070 (PMC4742566; doi:10.1016/j.celrep.2015.12.070)
Supplement: Document S1. Supplemental Experimental Procedures, Figures S1–S7, and Table S1 [file mmc1.pdf]

Cell Reports

Supplemental Information

The Gut Epithelial Receptor LRRC19 Promotes the Recruitment of Immune Cells and Gut Inflammation

Shuisong Cao, Xiaomin Su, Benhua Zeng, Hui Yan, Yugang Huang, Enlin Wang, Huan Yun, Yuan Zhang, Feifei Liu, Wenxia Li, Hong Wei, Yongzhe Che, and Rongcun Yang

1) **Supplemental data items**

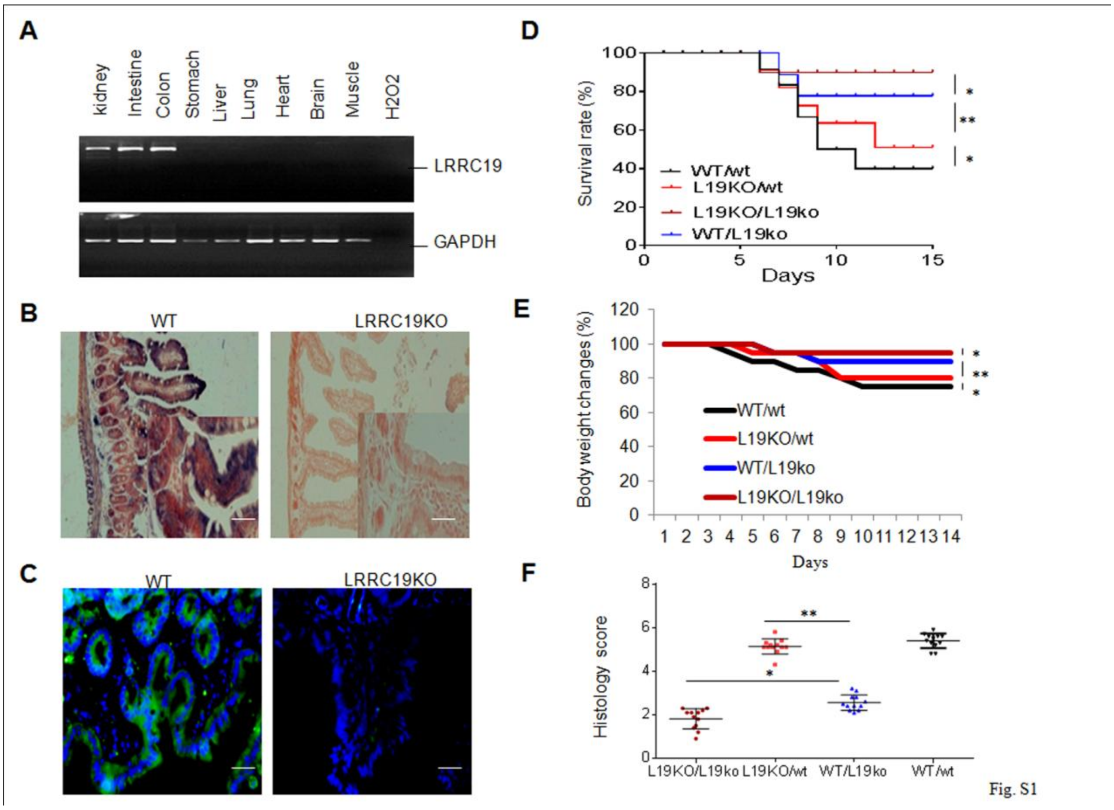

Figure S1. Related to Figure 1 and 2; DSS-mediated colitis in chimeric mice.
(A, B and C) RT-PCR, *in situ* hybridization and immune-staining of LRRC19 in gut tissues. Mouse kidney, intestine, colon, stomach, liver, lung, heart, brain and muscle tissues were analyzed using RT-PCR for LRRC19 expression (A). Mouse colon tissues from *wt* (WT) and *Lrrc19* KO (Lrrc19KO) mice were detected by incubation with the anti-sense probe of LRRC19 (B) or anti-LRRC19 antibody (C); blue in B and green in C indicated LRRC19. (D) Survival rates of different chimeric mice (n=16) after feeding 2% DSS-solution. Different chimeric mice were fed by a 2% DSS-solution in drinking water for 7 days, then switched to regular drinking water. (E) Changes of body weight among the different chimeric mice after feeding 2% DSS-solution in drinking water for 7 days. (F) Histology score of different chimeric mice after feeding 2% DSS-solution. Histological scores were assessed according to the methods described in supplementary EXPERIMENTAL PROCEDURES. WT/wt indicates that bone marrow (BM) cells of WT mice were transplanted into lethally irradiated WT mice; WT/L19ko indicates BM cells of WT mice into lethally irradiated *Lrrc19* KO mice; L19KO/wt indicates BM cells of *Lrrc19* KO mice into lethally irradiated WT mice; L19KO/L19ko indicates BM cells of *Lrrc19* KO mice into lethally irradiated *Lrrc19* KO mice. The data are representative of three independent experiments. *$P < 0.05$ and **$P < 0.01$ (Wilcoxon's test in A; ANOVA test in B; Mann-Whitney U test in C). Scale bars, 40 μm.

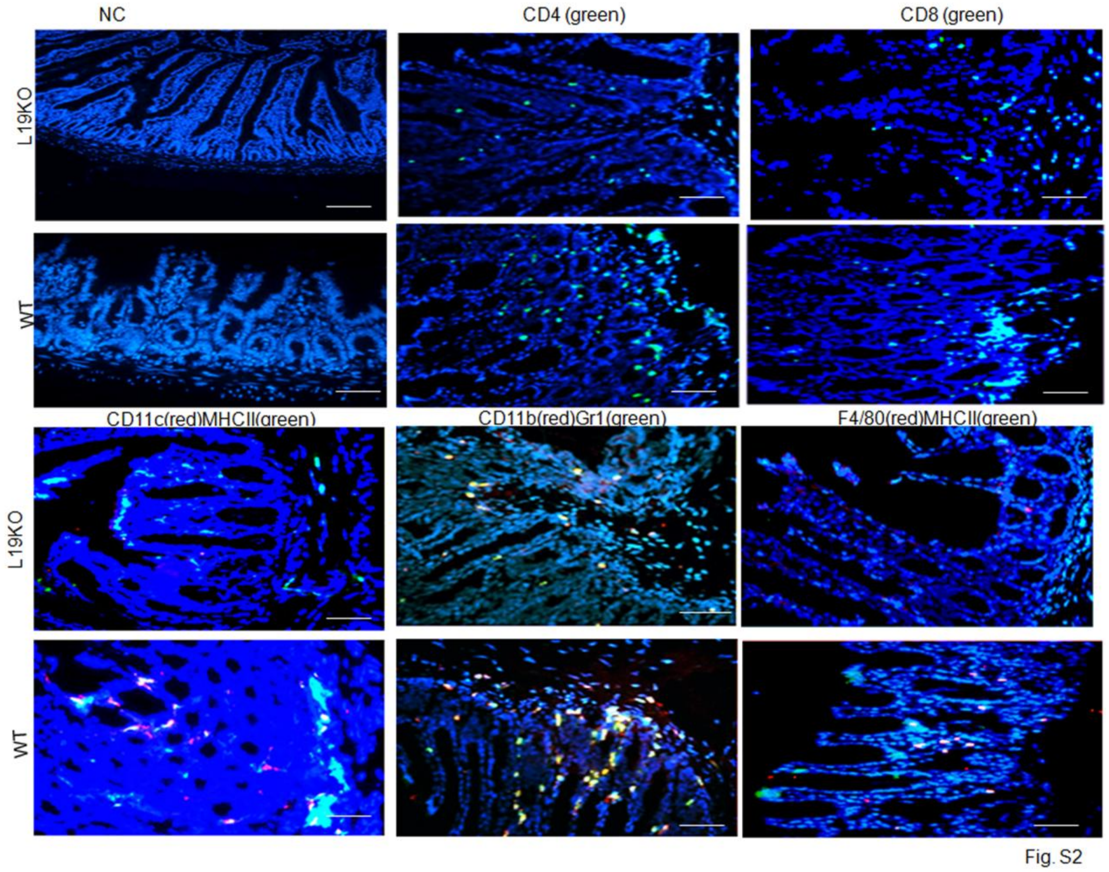

Figure S2. Related to Figure 3; Immuno-staining of CD4$^+$T cells, CD8$^+$ T cells, CD11c$^+$MHCII$^+$, CD11b$^+$Gr1$^+$ and F4/80$^+$MHCII$^+$ cells in *wt* (WT) and *Lrrc19* KO (L19KO) mice. The colon tissues were frozen, sliced, and stained by FITC-labeled anti-CD4 or anti-CD8, FITC-labeled anti- MHCII or anti-Gr-1, PE-labeled anti-CD11c, anti-CD11b or anti-F4/80 respectively. NC, isotypic control; Blue, DAPI staining. Scale bars, 40μm.

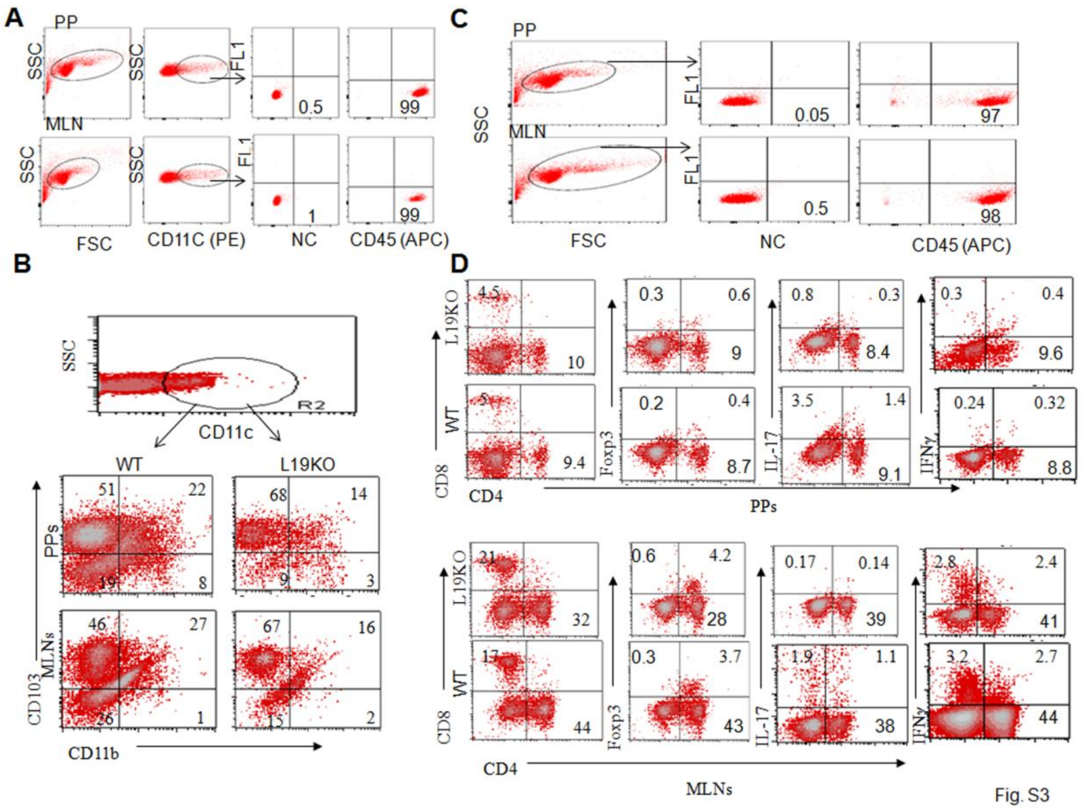

Figure S3. Related to Figure 3; (A) The percentages of CD45+ cells in the gated CD11c$^+$cells of PPs and MLNs by flow cytometry analyses; Numbers, percentages in the gated CD11c$^+$cells. NC, isotypic control. (B) The percentages of CD11c$^+$CD103$^+$CD11b$^-$ DCs (CD103$^+$DC), CD11C$^+$CD103$^+$CD11b$^+$DCs (DP-DC), CD11C$^+$CD11b$^-$ CD103$^-$ DCs (DNDC) and CD11C$^+$CD11b$^+$ (CD11b$^+$DC) in PPs and MLNs by flow cytometry analyses; Numbers, percentages in the gated CD11c$^+$cells. (C) The percentages of CD45+ cells in PPs and MLNs by flow cytometry analyses. NC, isotypic control. (D) The percentages of CD4$^+$, CD8$^+$, CD4$^+$Foxp3$^+$, CD4$^+$IL-17$^+$ and CD4$^+$IFNγ$^+$ cells in PPs and MLNs of *wt* (WT) and *Lrrc19* KO (L19KO) mice. PPs and MLNs were mashed, stained using the indicated antibodies, and subjected to flow cytometry. Cell percentages in age and gender matched *wt* (WT, n=6) and *Lrrc19* KO (L19KO, n=6) mice were compared.

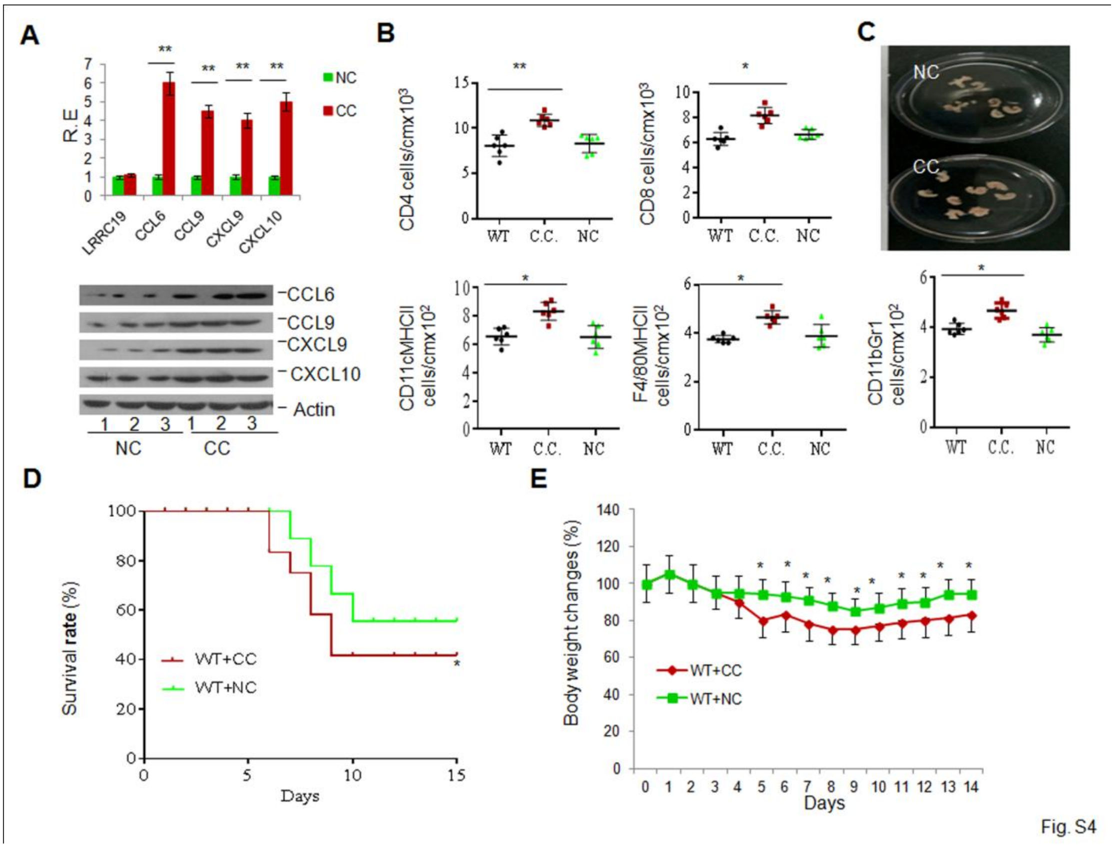

Figure S4. Related to Figure 4; Chemokines CCL6, CCL9, CXCL9 and CXCL10 promoted sensitivity to DSS-mediated colitis. (A) qRT-PCR and immunoblot of chemokines CCL6, CCL9, CXCL9 and CXCL10 in mice with (CC, chemokine complexes) or without (NC, negative control) CCL6, CCL9, CXCL9 and CXCL10 adenovirus injection. Actin, a loading control. (B) Absolute number of $CD4^+$ and $CD8^+$ T cells, $CD11C^+MHCII^+$ DCs, F4/80MHCII and $CD11b^+Gr1^+$ cells in the colon tissue of mice with (CC) or without (NC) chemokine adenovirus injection by flow cytometric analyses. CC: CCL6, CCL9, CXCL9 and CXCL10 complexes. WT: cell numbers from *wt* mice. (C) Size of MLNs from mice with (CC) or without (NC) chemokines expressing adenovirus injection. (D and E) Survival (D) and body weight (E) were monitored until day 14 after the start of DSS. Mice with (CC, n=18) or without (NC, n=18） CCL6, CCL9, CXCL9 and CXCL10 adenovirus injection were fed by a 2% DSS-solution in drinking water for 7 days, then switched to regular drinking water. *$P<0.05$, **$P<0.01$ (*t*-test in A, mean ± SD; Mann-Whitney U test in B; Wilcoxon's test in D; ANOVA test in E).

```
>A10253_18#_1407035775Y.seq.Contig1TGGATGGACCTGCGGTGCATTAG
CTAGTTGGTAAGGTAACGGCTTACCAAGGCAATGATGCATAGCCGAGTTG
AGAGACTGATCGGCCACATTGGGACTGAGACACGGCCCAAACTCCTACG
GGAGGCAGCAGTAGGGAATCTTCCACAATGGACGCAAGTCTGATGGAGC
AACGCCGCGTGAGTGAAGAAGGGTTTCGGCTCGTAAAGCTCTGTTGGTAG
TGAAGAAAGATAGAGGTAGTAACTGGCCTTTATTTGACGGTAATTACCTAG
AAAGTCACGGCTAACTACGTGCCAGCAGCCGCGGTAATACGTAGGTGGC
AAGCGTTGTCCGGATTTATTGGGCGTAAAGCGAGTGCAGGCGGTTCAATA
AGTCTGATGTGAAAGCCTTCGGCTCAACCGGAGAATTGCATCAGAAACTG
TTGAACTTGAGTGCAGAAGAGGAGAGTGGAACTCCATGTGTAGCGGTGGA
ATGCGTAGATATATGGAAGAACACCAGTGGCGAAGGCGGCTCTCTGGTCT
GCAACTGACGCTGAGGCTCGAAAGCATGGGTAGCGAACAGGATTAGATA
CCCTGGTAGTCCATGCCGTAAACGATGAGTGCTAAGTGTTGGGAGGTTTC
CGCCTCTCAGTGCTGCAGCTAACGCATTAAGCACTCCGCCTGGGGAGTAC
GACCGCAAGGTTGAAACTCAAAGGAATTGACGGGGGCCCGCACAAGCGG
TGGAGCATGTGGTTTAATTCGAAGCAACGCGAAGAACCTTACCAGGTCTT
GACATCCAGTGCAAACCTAAGAGATTAGGTGTTCCCTTCGGGGACGCTGA
GACAGGTGGTGCATGGCTGTCGTCAGCTCGTGTCGTGAGATGTTGGGTTA
AGTCCCGCAACGAGCGCAACCCTTGTCATTAGTTGCCATCATTAAGTTGG
GCACTCTAATGAGACTGCCGGTGACAAACCGGAGGAAGGTGGGGATGAC
GTCAAGTCATCATGCCCCTTATGACCTGGGCTACACACGTGCTACAATGG
ACGGTACAACGAGAAGCGAACCTGCGAAGGCAAGCGGATCTCTTAAAGC
CGTTCTCAGTTCGGACTGTAGGCTGCAACTCGCCTACACGAAGCTGGAAT
CGCTAGTAATCGCGGATCAGCACGCCGCGGTGAATACGTTCCCGGGCCT
TGTACACACCGCCCGTCACACCATGAGAGTCTGTAACACCCAAAGCCGGT
GGGATAACCTTTATAGGAGTCAG
```

Fig. S5

Figure S5. Related to Figure S6; Sequence of *Lacbacillus* NK6 16S rRNA by primers (Forward, 5'-AGAGTTTGATCMTGGCTCAG; Reverse, 5'-ACGGCTACCTTGTTACGACTT). This sequence has also been shown in another paper.

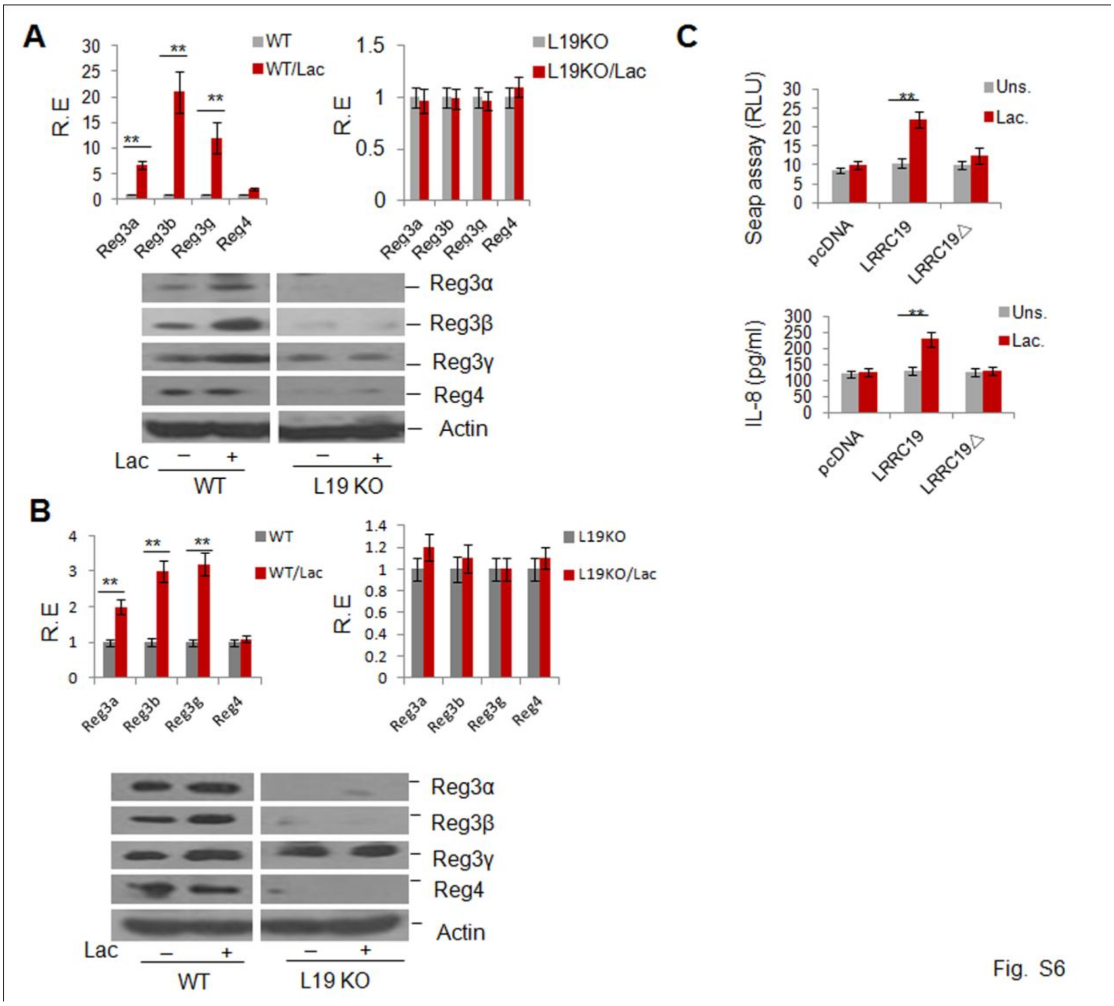

Figure S6. Related to Figure 6; *Lactobacillus* modulates the expression of REGs through LRRC19. (A) qRT-PCR and immunoblot of REG3α, REG3β, REG3γ and REG4 in the gut tissues of *wt* and *L19* KO mice in response to *Lactobacillus* NK6. A 4-cm gut segment was stimulated by infusion with *lactobacillus* NK6 and analyzed after 16 hrs. (B) qRT-PCR and immunoblot of REG3α, REG3β, REG3γ and REG4 in the gut epithelial cells of pan-antibiotics-treated *wt* and *L19KO* mice after transplanting *Lactobacillus* NK6 for 3 days. Antibiotics-treated mice were intragastrically given *Lactobacillus* (1×10^8) (n=3) and the expression of REG3α, REG3β, REG3γ and REG4 in gut epithelial cells was analyzed after 3 days. For antibiotics-treated mice, 6- to 8-week-old mice were treated with ampicillin (A, 1 g/L, Sigma), vancomycine (V, 0.5g/L), neomycin sulfate (N, 1 g/L), and metronidazole (M, 1g/L) in drinking water for 4 weeks via the drinking water. Water containing antibiotic was exchanged every three days. To confirm the elimination of bacteria, stool was collected from antibiotic-treated and -untreated mice and cultured in anaerobic and aerobic condition. The bacteria were counted under microscope. (C) NF-κB (upper) activity and endogenous cytokine production (lower) in LRRC19-transfected 293T cells after exposed to *Lactobacillus*. 293T cells were co-transfected with pNF-κB-SEAP (secreted alkaline phosphatase) and the expression plasmids of LRRC19 or LRRC19Δ (absence of extracellular region) by Lipofectamine 2000 (Invitrogen). Alkaline phosphatase activity in the supernatants was measured following treatment with *Lactobacillus*. Uns, untreated cells. IL-8 secretion was detected by ELISA kit according to the manufacturer's protocol (Jingmei Corp.). The empty expression vector (pcDNA3.1) was used as control. RLU, relative light

units. *P<0.05, **P<0.01 (*t*-test; mean ± SD). The data are a representative of three independent experiments.

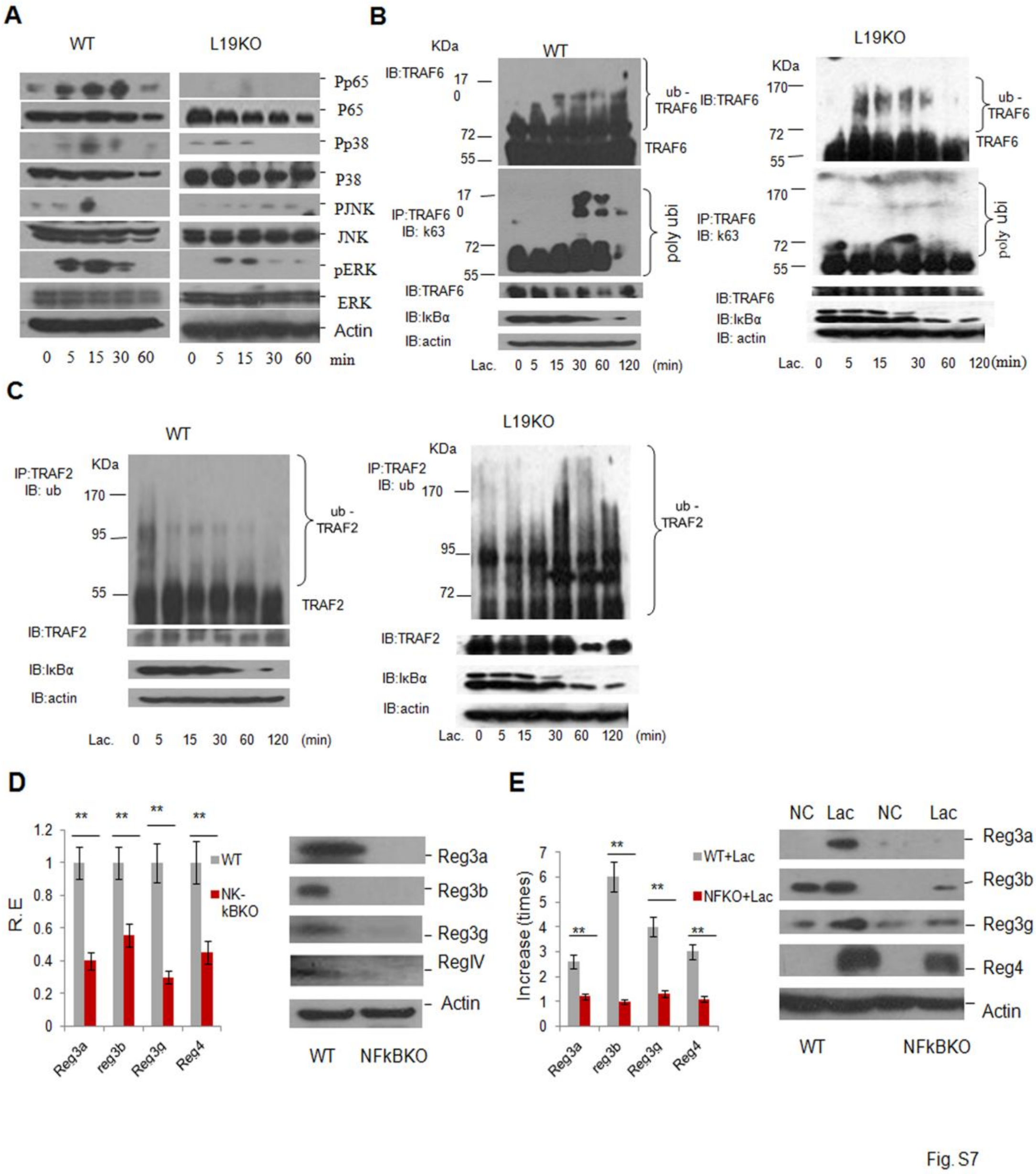

Figure S7. Related to Figure 6; LRRC19 induces expression of REGs through TRAF2 and TRAF6 ubiquitination mediated signal pathway. (A) Phosphorylation analyses of p38, JNK, and ERK. The cells were lysed at the indicated time. A 4-cm gut segment was stimulated by infusion with *lactobacillus* NK6 and analyzed at the indicted time points. The gut mucosa was scraped and lysed, and phosphor-p38, -ERK and -JNK were detected using anti-phosphor-p38, -ERK or-JNK antibody. Actin, a loading control. (B) Immunoblot of K63-linked ubiquitination (K63-Ub) of endogenouse TRAF6

immunoprecipitated from *wt* and *Lrrc19* KO (L19KO)gut epithelial cells stimulated with *lactobacillus* NK6 (middle blot), and immunoblot analysis of TRAF6 (top blot), IkBα and actin (below blots) in the same cells without immunoprecipitation. IP, immunoprecipitation; IB, immunoblot assay. (C) Immunoblot analysis of ubiquitination of endogenouse TRAF2 immunoprecipitated from *wt* and *Lrrc19* KO gut epithelial cells stimulated with *lactobacillus* NK6 (upper blots), and immunoblot analysis of TRAF2, IκBα and actin (below blots) in the same cells without immunoprecipitation. (D) qRT-PCR (right) and immunoblot (left) of REG3α, REG3β, REG3γ and REG4 in *wt* and *Nf-κb* KO (L19KO) gut tissues. (E) qRT-PCR (right) and immunoblot (left) of REG3α, REG3β, REG3γ and REG4 in gut epithelial cells of *wt* and *Nf-κb* KO mice after exposed to *Lactobacillus* NK6. *P<0.05, **P<0.01 (*t*-test in D and E; mean ± SD)

Table S1. Related to Figure 6; The profiling of bacteria genus in ileum (proximal), ileum (distal), caecum and colon.

Genus	Ileu （proximal）		Ileum （distal）		Caecum		Colon	
	WT	KO	WT	KO	WT	KO	WT	KO
Enterorhabdus	12	12	5	37	3	1	1	2
Coriobacteriaceae;Other	61	36	47	132	16	5	4	7
Actinobacteria;Other;Other;Other	6	12	0	11	5	0	0	0
Porphyromonadaceae; Other	19	21	20	25	13	4	8	23
Parabacteroides	0	1	4	15	17	28	15	26
Prevotellaceae;Other	30	49	28	723	799	897	960	1424
Paraprevotella	7	22	0	20	214	272	13	25
Alistipes	4	0	5	5	36	14	18	28
Rikenella	0	0	75	20	0	8	9	35
Bacteroides	2	11	6	95	154	273	74	160
Bacteroidales;Other;Other	19	0	75	341	17	12	110	245
Bacteroidetes;Other;Other;Other;Other	830	1153	2634	1982	607	731	534	781
Achromobacter	26	37	56	216	2	2	0	0
Parasutterella	207	258	118	87	36	48	18	44
Helicobacter	281	329	11	191	53	62	25	163
Serratia	13	7	3	22	0	0	0	1
Stenotrophomonas	2	0	0	6	0	0	0	0
Proteobacteria;Other;Other;Other;Other	6	0	0	0	4	2	8	11
Mycoplasma	18	107	1	4	1	9	1	14
Ureaplasma	21	27	99	2039	1	3	1	13
Jeotgalicoccus	4	0	2	0	0	0	0	0
Aerococcus	1	0	2	0	0	0	0	0
Enterococcus	1	0	2	0	0	0	0	0
Lactobacillus	546	120	833	281	57	20	13	31
Streptococcaceae;Other	1	0	-	-	0	0	-	-
Streptococcus	3	0	9	21	0	0	0	0

Clostridium_XlVb	2	2	1	14	30	13	32	28
Dorea	-	-	4	1	2	0	2	0
Marvinbryantia	-	-	2	1	4	7	3	5
Lachnospiraceae;Other	111	156	307	230	1717	1260	1089	1257
Clostridiales;Other;Other	44	53	129	95	1368	548	912	306
Clostridium_XI	32	57	2	0	10	0	3	1
Anaerotruncus	1	5	1	3	30	24	8	9
Butyricicoccus	0	2	3	4	55	12	10	18
Oscillibacter	2	3	0	8	79	44	42	38
Ruminococcaceae;Other	12	11	34	36	188	147	103	133
Clostridia;Other;Other;Other	2	29	2	3	229	40	15	8
Clostridium_XVIII	2	6	6	8	6	5	3	3
Firmicutes;Other;Other;Other;Other	39	18	152	22	372	107	28	26
Bacteria;Other;Other;Other;Other;Other	1767	2604	528	840	806	936	15	26

Notes: Wt, wild type mice; KO, *Lrrc19* KO mice.

2) Supplemental Experimental Procedures

Reagents

Anti-mouse p38 (D13E1, Cell Signaling), JNK (sc-137020, Santa), ERK (197G2, Cell Signaling), STAT3 (124H6, Cell Signaling), phosphorylated-STAT3 (EP2147Y, Abcam), phosphorylated-p38 (pp38) (D3F9, Cell Signaling), phosphorylated JNK (sc-81502, Santa), phosphorylated ERK (D13.14.4E, Cell Signaling), phosphorylated -IκBα (14D4, Cell Signaling), IκBα (L35A5, Cell Signaling), p-65 (sc8008, Santa), phosphorylated-p-65 (sc-52401, Santa), LRRC19 (PA5-20914, Thermo), and β-actin (sc-47778, Santa) were purchased; FITC-, PE- or APC-conjugated anti-mouse CD4 (RM4-5, Biolegend), CD8 (ZUT270.5, Biolegend), MHCII (I-A/I-E, M5/114.115.2, Biolegend), CD11c (MCA1441GA, Biolegend), CD103 (2E7, Biolegend), CD11b (M1/70, eBioscience), F4/80 (BM8, Biolegend), Gr-1 (RB6-8C5, eBioscience), IFNγ (XMG1.2, Biolegend), Foxp3 (MF23, eBioscience) and IL17A (eBio17B7, eBioscience) antibodies were purchased. Anti- ubiquitin (YT4793, Immunoway), K63-Ub (HWA4C4, Enzo), TRAF2 (EPR6048, Epitomics), TRAF6 (sc-8408, Santa) antibodies; anti-mouse CCL6 (EPR14614, Abcam), CCL9 (ab9913, Abcam), CXCL9 (orb13424, Biorbyt), CXCL10 (ab9938, Abcam), Reg3α (M-44, Santa), Reg3β (AF5110, Sigma), Reg3γ (PA517, Thermo) and Reg4 (H-40, Santa) were also purchased. HA- or flag- tagged LRRC19, CCL6, CCL9, CXCL9, CXCL10 Reg3α, Reg3β, Reg3γ and Reg4 adenoviruses were prepared by ABM, Canada and expanded by GeneChem, Shanghai.

DSS induced colitis

Dextran sodium sulfate (DSS) induced colitis was performed according to the reported method (Chen et al., 2013) with modification. Briefly, mice received 2% (wt/vol) DSS (40,000 kDa; ICN Biochemicals) or indicated doses in their drinking water for 7 days, then switched to regular drinking water. The amount of DSS water drank per animal was recorded and no differences in intake between strains were observed. For survival studies, mice were followed for 14 days post start of DSS-treatment. Mice were weighed every other day for the determination of percent weight change. This was calculated as: % weight change = (weight at day X-day 0 / weight at day 0) × 100. Animals were monitored clinically for rectal bleeding, diarrhea, and general signs of morbidity, including hunched posture and failure to groom. Mice were then sacrificed at the indicated days for histological study. Representative colon tissues were embedded in paraffin for hematoxylin/eosin (H&E) staining or embedded in OCT compound (Tissue-Tek, Sakura, Torrance, CA) and frozen over liquid nitrogen for immuno-staining. For histological evaluation, colonic epithelial damage was scored blindly as follows: 0 = normal; 1 = hyper-proliferation, irregular crypts, and goblet cell loss; 2 = mild to moderate crypt loss (10–50%); 3 = severe crypt loss (50–90%); 4 = complete crypt loss, surface epithelium intact; 5 = small to medium sized ulcer (<10 crypt widths); 6 = large ulcer (>10 crypt widths) (Lee et al., 2006).

Tumorigenesis Procedure

Tumorigenesis procedure was performed according to the reported method (Greten et al., 2004). Briefly, 7-8-week-old mice were injected with AOM (Sigma) dissolved in 0.9% NaCl intraperitoneally at a dose of 12.5 mg/kg body weight. 5 days after injection, mice were treated with 2 % DSS in drinking water, then followed by regular water for 16 days. This cycle was repeated twice (at the third cycle, mice were treated with 2.0% DSS for 4 days). 2 weeks after DSS treatment, mice were sacrificed and murine colon was removed and flushed carefully with PBS buffer. Colon was then cut longitudinally

and fixed flat in 10% neutral buffered formalin overnight. All of the colon tumors were counted and measured under a stereomicroscope. Representative colon cancer tissues were embedded in paraffin for hematoxylin/eosin (H&E) staining or embedded in OCT compound and frozen over liquid nitrogen for immuno-staining. Histology analysis was carried out on H&E or immuno-staining tumor sections.

Histological and immune staining

For hematoxylin/eosin (H&E) staining, previously reported methods were used in this experiment (Su et al., 2014)**.** Briefly, the entire colon was excised to measure the length of the colon and then were fixed in 4% (w/v) paraformaldehyde buffered saline and embedded in paraffin, 5 μm sections colon sections were cut and stained with H&E.

For immune staining, colon tissues were embedded in OCT compound (Tissue-Tek, Torrance, CA) and frozen over liquid nitrogen. 5-μm-thick sections were prepared from frozen tissue and fixed in acetone (−20°C) for 10 min. After rehydration in PBS for 5 min and further washing in PBS, tissue sections were blocked with 1% (w/v) BSA and 0.2% (w/v) milk powder in PBS (PBS-BB). The primary antibody was added in PBS-BB and incubated overnight at 4℃. After PBS washing (three times, 5 min each), tissue was detected with DAB kit or fluorescence labeled second antibody. Nuclei were stained by DAPI.

For BrdU assay, 1 mg/ml of BrdU in PBS was intraperitoneally injected to mice. Mice were sacrificed in 4 hrs after BrdU injection. The same segment of distal colon was fixed in 4% (w/v) paraformaldehyde and embedded by paraffin. Proliferating cells were detected with BrdU detection kit (BD Bioscience).

***In situ* hybridization**

For *in situ* hybridization, previously reported methods were used in this experiment (Su et al., 2014). Briefly, tissues of mice were fixed in 4% (w/v) paraformaldehyde-fixed and embedded in paraffin according to standard procedures. Briefly, 5 μm sections were deparaffinized, rehydrated through a graded series of ethanol baths, and washed with water treated with 0.1% DEPC three times for 5 min. In situ hybridization of LRRC19 was performed according to the manufacturer's protocol (TBD sci). Staining was conducted using a diaminobenzidine staining kit. Probes listed in Table S2d, were designed based on the published genomic sequences of mouse LRRC19 mRNA (GenBank ID: 224109). Probes were labeled with digoxin (DIG). The sense probes were used as negative controls.

Gut microbiota analysis

Gut microbiota was analyzed by Huada Biotechnology company (ShenZhen, China) using primers that target the V1-V3 regions of the 16S rRNA (Jeraldo et al., 2011). Once the PCR for each sample was completed, the amplicons were purified using the QIAquick PCR purification kit (Qiagen Valencia, CA), quantified, normalized, and then pooled in preparation for emulsion PCR followed by sequencing using Titanium chemistry (Roche, Basel Switzerland) according to the manufacturer's protocol. In the first step of data processing, the generated sequence data were de-convolved using the sample barcodes to identify sequences from each of the samples. Barcode, primer, and adaptor sequences were also trimmed as part of this step. PCR artifacts "chimeras" were identified using the ChimeraSlayer program (http://microbiomeutil.sourceforge.net; reference http://genome.cshlp.org/content/21/3/494.long) and removed prior to downstream analysis. The resulting de-convoluted and filtered sequence data were assigned taxonomy (to the genus level) using the Ribosomal Database Project (RDP)

classifier and the genera classifications were used to generate a sample-genus count matrix. Operational Taxonomic Unit (OTU) analysis of these sequences was performed as follows: sequences were processed (trimmed) using the Mothur software and subsequently clustered at 97% sequence identity using cd-hit to generate OTUs. The OTU memberships of the sequences were used to construct a sample-OTU count matrix. The samples were clustered at genus and OTU levels using the sample-genus and sample-OTU count matrices respectively. For each clustering, Morisita-Horn dissimilarity was used to compute a sample distance matrix from the initial count matrix, and the distance matrix was subsequently used to generate a hierarchical clustering using Ward's minimum variance method. The Wilcoxon Rank Sum test was used to identify OTUs that had differential abundance in the different sample groups.

Quantitave PCR (qPCR) was performed on genomic DNA extracted from the colon contents of WT or *LRRC19* KO mice as described above. qPCR was performed and analyzed using SYBR green I dye chemistry and 7300 Real-time PCR systems and software (Applied Biosystem).

Preparation of adenovirus vectors

All adenovirus vectors used in this study were produced by Applied Biological Materials (ABM) Inc..(Richmond, BC, Canada) and were amplified by Genechem Inc. Shanghai, China. ABM Inc.'s adenovirus CMV expression system provides an efficient method for constructing recombinant adenoviruses. This procedure uses *in vitro* ligation to subclone the gene of interest into a replication-incompetent (-E1/-E3) human adenoviral type 5 (Ad5) genome. First, the gene was cloned into pShuttle to producing recombinant adenoviral DNA. This process includes that PI-SceI/I-CeuI digestion of recombinant pShuttle, subclone the expression cassette of the pShuttle vector into the pAdeno genome, transform *E. coli* with ligation products and analyze the adenoviral DNA by PCR or restriction digestion. Then PI-SceI and I-CeuI restriction was analyzed and pAdeno DNA for the presence of pShuttle derived expression cassettes was screened by PCR with adeno forward and reverse PCR primers. Adenoviral DNA for transfection was prepared and adenoviruses were generated in 293 Packaging Cells. Finally, the recombinant adenoviral vectors were amplified and adenoviral titre was determined.

Oligoes used in this study

(a) Oligoes used in cloning LRRC19.

Oligo name	Sequence (5'-3')	
MLRRC19F<br>MLRRC19R	ATGAAAGTCACACGCTTCATGTTTTGGC<br>CTTTTCTTCATGTACCTCATTGATATCT	For cloning murine LRRC19

(b) Oligoes used in RT-PCR (semi-quantitative RT-PCR).

Oligo name	Sequence (5'>3')	Description
Murine LRRC19-s<br>Murine LRRC19-as	ATGAAAGTCACACGCTTCATG<br>AATTACTTTTCTTCATGTACCTCA	RT-PCR for murine full-length LRRC19 detection
GAPDH-s<br>GAPDH-as	GTGGCAAAGTGGAGATTGTTG<br>CAGTCTTCTGGGTGGCAGTGAT	RT-PCR for murine GAPDH detection

(c) Oligos used for quantitative RT-PCR (qRT-PCR).

Oligo name	Sequence (5'>3')	Description

Murine GAPDH-s<br>Murine GAPDH-as	TCAACGGCACAGTCAAGG<br>TACTCAGCACCGGCCTCA	qRT-PCR for murine GAPDH detection
Murine β-actin-s<br>Murine β-actin-as	ATCATTGCTCCTCCTGAGCG<br>GCTGATCCACATCTGCTGGAA	qRT-PCR for murine β-actin detection
Murine LRRC19-s<br>Murine LRRC19-as	CCCAGATGAGCTAAAGCACGA<br>GAAAGCCCAGCTTTTCCCAAG	qRT-PCR for murine LRRC19 detection
Murine TNFα-s<br>Murine TNFα-as	GGTCTGGGCCATAGAACTGA<br>CAGCCTCTTCTCATTCCTGC	qRT-PCR for murine TNF-alpha detection
Murine IL-6-s<br>Murine IL-6-as	TCTGAAGGACTCTGGCTTTG<br>GATGGATGCTACCAAACTGGA	qRT-PCR for murine IL-6 detection
Murine IL-1β-s<br>Murine IL-1β-as	GTGTCTTTCCCGTGGACCTT<br>AATGGGAACGTCACACACCA	qRT-PCR for murine IL-1β detection
Murine IFNγ-s<br>Murine IFNγ-as	AACGCTTACACACTGCATCTTGG<br>GACTTCAAAGAGTCTGAGG	qRT-PCR for murine IFNγ detection
Murine IL-17-s<br>Murine IL-17-as	ACTACCTCAACCGTTCCACG<br>ATGTGGTGGTCCAGCTTTCC	qRT-PCR for murine IL-17 detection
Murine IL-12-s<br>Murine IL-12-as	TGGTTTGCCATCGTTTTGCTG<br>ACAGGTGAGGTTCACTGTTTCT	qRT-PCR for murine IL-12 detection
Murine CCL6 s<br>Murine CCL6 as	TATCCTTGTGGCTGTCCTTGG<br>TTACATGGGATCTGTGTGGCA	qRT-PCR for murine CCL6 detection
Murine CCL9-s<br>Murine CCL9-as	GCCCAGATCACACATGCAAC<br>AGGACAGGCAGCAATCTGAA	qRT-PCR for murine CCL9 detection
Murine CXCL9-s<br>Murine CXCL9-as	CCGAGGCACGATCCACTAC<br>AGGCAGGTTTGATCTCCGTT	qRT-PCR for murine CXCL9 detection
Murine CXCL10-s<br>Murine CXCL10-as	CAAGTGCTGCCGTCATTTTCT<br>ATAGGCTCGCAGGGATGATT	qRT-PCR for murine CXCL10 detection
Murine Reg3α-s<br>Murine Reg3α-as	CAAGGCTTATCGCTCCCACT<br>ACGAGATGTCCTGAGGGTCT	qRT-PCR for murine Reg3α detection
Murine Reg3β-s<br>Murine Reg3β-as	ATACCCTCCGCACGCATTAG<br>GGCCAGTTCTGCATCAAACC	qRT-PCR for murine Reg3β detection
Murine Reg3γ-s<br>Murine Reg3γ-as	TTCCTGTCCTCCATGATCAAA<br>CATCCACCTCTGTTGGGTTC	qRT-PCR for murine Reg3γ detection
Murine Reg4-s<br>Murine Reg4-as	CTGTGTGGATTGGCCTGCAT<br>GCAATGCCTGGCTTCACTCT	qRT-PCR for murine Reg4 detection

(d) Probes used in *in situ* hybridization.

Oligo name	Sequence (5’-3’)	Targeted sequence:

Murine LRRC19 probe 1	AGCTGTGGTGCATCGAAGAGGCGTATC	Murine LRRC19 in Gene-bank NM_175305
Murine LRRC19 probe 2	TGATAGCAATGAAGATAAGCAGTGAAGT T	Murine LRRC19 in Gene-bank NM_175305
Murine LRRC19 probe 3	CAGGCGATGGTGGTTGTAACTCAGCAG	Murine LRRC19 in Gene-bank NM_175305
Mockprobe	Offered by company	

(e) Oligoes used in identification of gut microbiota.

Oligo name	Sequence (5'-3')	Description
Bacteroides Phylum	F: 5-GAGAGGAAGGTCCCCCAC-3<br>R: 5-CGCTACTTGGCTGGTTCAG-3	rRNA-16S
Firmicutes Phylum	F: 5-GCTGCTAATACCGCATGATATGTC-3<br>R: 5-CAGACGCGAGTCCATCTCAGA-3	rRNA-16S

3) Supplemental References:

Chen, K., Liu, M., Liu, Y., Yoshimura, T., Shen, W., Le, Y., Durum, S., Gong, W., Wang, C., Gao, J.L., *et al.* (2013). Formylpeptide receptor-2 contributes to colonic epithelial homeostasis, inflammation, and tumorigenesis. The Journal of clinical investigation *123*, 1694-1704.

Greten, F.R., Eckmann, L., Greten, T.F., Park, J.M., Li, Z.W., Egan, L.J., Kagnoff, M.F., and Karin, M. (2004). IKKbeta links inflammation and tumorigenesis in a mouse model of colitis-associated cancer. Cell *118*, 285-296.

Jeraldo, P., Chia, N., and Goldenfeld, N. (2011). On the suitability of short reads of 16S rRNA for phylogeny-based analyses in environmental surveys. Environmental microbiology *13*, 3000-3009.

Lee, J., Mo, J.H., Katakura, K., Alkalay, I., Rucker, A.N., Liu, Y.T., Lee, H.K., Shen, C., Cojocaru, G., Shenouda, S., *et al.* (2006). Maintenance of colonic homeostasis by distinctive apical TLR9 signalling in intestinal epithelial cells. Nature cell biology *8*, 1327-1336.

Su, X., Min, S., Cao, S., Yan, H., Zhao, Y., Li, H., Chai, L., Mei, S., Yang, J., Zhang, Y., *et al.* (2014). LRRC19 expressed in the kidney induces TRAF2/6-mediated signals to prevent infection by uropathogenic bacteria. Nature communications *5*, 4434.
