# Supplementary material for: The Gut Epithelial Receptor LRRC19 Promotes the Recruitment of Immune Cells and Gut Inflammation
Source: Cell Rep. 2016 Jan 14;14(4):695–707. doi: 10.1016/j.celrep.2015.12.070 (PMC4742566; doi:10.1016/j.celrep.2015.12.070)
Supplement: Document S2. Article plus Supplemental Information [file mmc2.pdf]

Cell Reports

Article

# The Gut Epithelial Receptor LRRC19 Promotes the Recruitment of Immune Cells and Gut Inflammation

## Graphical Abstract

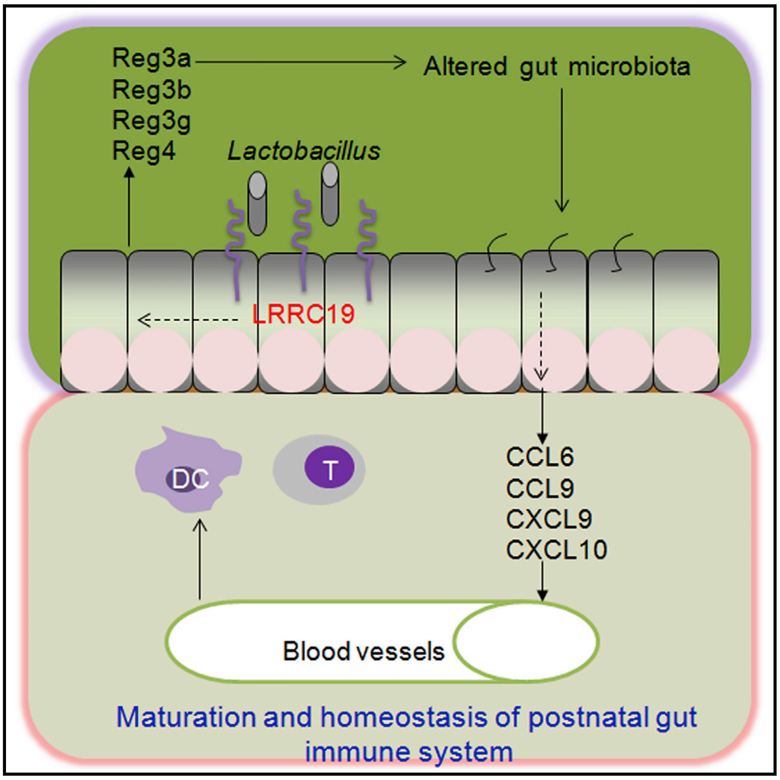


## Highlights

- The gut immune receptor LRRC19 is involved in host-microbiota interactions
- LRRC19-associated chemokines control immune cell recruitment and gut inflammation
- Chemokines are regulated by REG protein-mediated gut microbiotas
- *Lactobacillus* may modulate the expression of REG proteins through LRRC19

## Authors

Shuisong Cao, Xiaomin Su, Benhua Zeng, ..., Hong Wei, Yongzhe Che, Rongcun Yang

## Correspondence

ryang@nankai.edu.cn

## In Brief

Cao et al. found that the gut epithelial receptor LRRC19 is involved in gut host-microbiota interactions and that it plays a critical role in promoting the recruitment of immune cells and intestinal inflammation.

## Accession Numbers

GSE62487

Cao et al., 2016, Cell Reports *14*, 695–707
February 2, 2016 ©2016 The Authors
http://dx.doi.org/10.1016/j.celrep.2015.12.070

OPEN ACCESS
CellPress

# The Gut Epithelial Receptor LRRC19 Promotes the Recruitment of Immune Cells and Gut Inflammation

Shuisong Cao,[1,5] Xiaomin Su,[1,5] Benhua Zeng,[4,5] Hui Yan,[1,5] Yugang Huang,[1] Enlin Wang,[1] Huan Yun,[1] Yuan Zhang,[1] Feifei Liu,[1] Wenxia Li,[4] Hong Wei,[4] Yongzhe Che,[1] and Rongcun Yang[1,2,3,*]
[1]Department of Immunology, Nankai University School of Medicine, Nankai University, Tianjin 300071, China
[2]State Key Laboratory of Medicinal Chemical Biology, Nankai University, Tianjin 300071, China
[3]Key Laboratory of Bioactive Materials, Ministry of Education, Nankai University, Tianjin 300071, China
[4]Department of Laboratory Animal Science, College of Basic Medicine Science, Third Military Medical University, Chongqing 404100, China
[5]Co-first author
*Correspondence: ryang@nankai.edu.cn
http://dx.doi.org/10.1016/j.celrep.2015.12.070
This is an open access article under the CC BY license (http://creativecommons.org/licenses/by/4.0/).

## SUMMARY

**Commensal microbes are necessary for a healthy gut immune system. However, the mechanism involving these microbes that establish and maintain gut immune responses is largely unknown. Here, we have found that the gut immune receptor leucine-rich repeat (LRR) C19 is involved in host-microbiota interactions. LRRC19 deficiency not only impairs the gut immune system but also reduces inflammatory responses in gut tissues. We demonstrate that the LRRC19-associated chemokines CCL6, CCL9, CXCL9, and CXCL10 play a critical role in immune cell recruitment and intestinal inflammation. The expression of these chemokines is associated with regenerating islet-derived (REG) protein-mediated microbiotas. We also found that the expression of REGs may be regulated by gut *Lactobacillus* through LRRC19-mediated activation of NF-κB. Therefore, our study establishes a regulatory axis of LRRC19, REGs, altered microbiotas, and chemokines for the recruitment of immune cells and the regulation of intestinal inflammation.**

## INTRODUCTION

The gut lymphoid system constitutes the largest part of the whole immune system and includes organized tissues such as Peyer patches (PPs) and the mesenteric lymph nodes (MLNs) as well as lymphoid tissues scattered throughout the lamina propria (LP) and the epithelium of the mucosa (Mowat and Viney, 1997). This gut immune system contains both innate immune cells, such as dendritic cells (DCs), macrophages, and immature myeloid cells, and adaptive immune cells, such as CD4 and CD8 lymphocytes and their subsets, including Th1, Th17, regulatory T (Treg), Th17$^{+}$Foxp3$^{+}$, and innate T cells. These cells perform crucial roles in protecting the body from foreign pathogens and establishing the immunological tolerance in the gut tissues. However, the exact mechanisms underlying recruitment, retention, and differentiation of these immune cells in gut tissues are unclear. Studies have suggested that microbe-recognizing molecules expressed on intestinal epithelial cells can mediate gut microbe-host immune cross-talk, integrating and transmitting signals from bacteria to the mucosal innate and adaptive immune cells (Goto and Ivanov, 2013). When the host lacks pathogen-recognizing molecules, such as myeloid differentiation primary response gene 88 (MyD88, an adaptor molecule in the Toll-like receptor [TLR] signaling pathway), nucleotide-binding oligomerization domain-containing protein 2 (NOD2), or NOD1, it is unable to clear invading pathogens from the gut tissue (Jiang et al., 2013; Luddy et al., 2014). However, under normal physiological conditions, intestinal epithelial cells show only low-level expression of these pathogen-recognizing receptors, which are generally unresponsive to TLR stimuli (Abreu et al., 2001; Melmed et al., 2003), suggesting that these receptors are not essential for the establishment and maintenance of the gut immune system (de Kivit et al., 2014).

Commensal bacteria are necessary for gut immune responses and inflammation under normal physiological conditions. These commensal microbes develop together with the gut immune system from birth and play a crucial role in the maturation of the intestinal mucosal immune system of their host (Clemente et al., 2012). Indeed, germ-free (GF) mice differ from normal mice in the number of DCs and innate lymphoid cells, although all major innate immune subsets are present in these mice (Niess and Adler, 2010; Sawa et al., 2010). Additionally, the absence of intestinal bacteria in GF mice may dramatically reduce the frequency of oncogenic mutations and tumor formation (Dove et al., 1997; Li et al., 2012). Fecal and bacteria transplantations have demonstrated that the gut microbiota can restore the number of immune cell populations in the gut immune system (Round and Mazmanian, 2009) and the gut's sensitivity to tumor-inducing factors (Zhan et al., 2013). Recent studies have also shown that commensal bacterial composition can influence the type and robustness of the host's immune responses (Ivanov and Honda, 2012). The immunomodulatory roles of several

CrossMark

commensal bacterial species have been demonstrated (Ivanov and Honda, 2012). However, how these gut commensal microbes exert their effects on the gut immune response and inflammation is largely unknown.

LRRC19 belongs to the leucine-rich repeat (LRR) family and differs from other pathogen-recognizing receptors because it contains no cytoplasmic Toll/interleukin 1 (IL-1) receptor (TIR) domain. LRRC19 is highly expressed in gut epithelial cells under normal physiological conditions and is activated by multiple TLR ligands and *E. coli* (Chai et al., 2009; Su et al., 2014). Here we found that LRRC19 is involved in gut host-microbiota interactions and that it plays a critical role in promoting the recruitment of immune cells and intestinal inflammation.

## RESULTS

### LRRC19 Deficiency Reduces Inflammatory Responses in Gut Tissues

Our previous studies have suggested that gut epithelial cells express LRRC19 (Chai et al., 2009; Su et al., 2014). Here we used RT-PCR, in situ hybridization, and immunostaining to confirm these findings (Figures S1A–S1C). LRR family members such as TLRs, which are expressed in gut epithelial cells, play a significant role in enteritis, colitis, colon cancer, and metabolism-associated diseases (Rakoff-Nahoum et al., 2006; Vijay-Kumar et al., 2010). Because LRRC19 belongs to the LRR family, we first undertook a long-term observational study of *Lrrc19* knockout (KO) mice to determine the effects of LRRC19 on mouse health. *Lrrc19* KO mice displayed increased longevity compared with the cohoused wild-type (WT) littermates. Almost all *Lrrc19* KO (male and female) mice remained alive, whereas most of the WT mice (>80%) had died after 2 years on standard chow (Figure 1A). *Lrrc19* KO mouse body weights were lower than those of WT mice (Figure 1A). Interestingly, when the gut tissues of these mice were examined, almost all WT mice showed slight inflammation (low-grade enteritis and colitis) whereas *Lrrc19* KO mouse gut tissues did not. The gut tissues of *Lrrc19* KO mice were more yellow (Figure 1B) and the colon tissues were thinner in *Lrrc19* KO mice than in WT mice, and the ceca of *Lrrc19* KO mice were enlarged significantly (Figure 1C), as in GF mice (Aluwihare, 1971). These features of *Lrrc19* KO mice suggest a lack of immunological inflammatory responses in their gut tissues. Expression of cytokines such as tumor necrosis factor $\alpha$ (TNF-$\alpha$), IL-1$\beta$, IL-6, interferon $\gamma$ (IFN$\gamma$), IL-17, and IL-12 was lower in the gut tissues of *Lrrc19* KO mice than in those of WT mice over the long term (Figure 1D). Colitis-associated phospho-nuclear factor $\kappa$B (NF-$\kappa$B) p65, and -STAT3, which were detected readily in WT mice, were barely detectable in *Lrrc19* KO mice (Figure 1D). A histological examination of the LP showed a sparse stroma in *Lrrc19* KO mice, and the colonic sections from these mice showed no overt inflammatory infiltrate in the LP, which was observed in most WT mice (Figure 1E). Proliferating cell nuclear antigen (PCNA), a proliferative cell marker, was immunoreactive in numerous WT colon epithelial cells but not in *Lrrc19* KO mouse epithelial cells. $\beta$-Catenin and cyclooxygenase 2 (COX2), colon cancer-associated markers, were also detected more readily in WT mice compared with *Lrrc19* KO mice (Figure 1F). All of these data imply that LRRC19 is associated with enteritis, colitis, and colitis-associated tumorigenesis.

We next used dextran sodium sulfate (DSS)-induced colitis and an azoxymethane (AOM)-DSS-induced colon cancer model to examine the effects of LRRC19 on the occurrence and development of colitis and colon cancer. The results showed that LRRC19 deficiency conferred a marked resistance to DSS-mediated colitis. Although WT mice showed clear symptoms of colitis after exposure to 2.0% DSS in their drinking water for 7 days, the colitis symptoms, including survival rate, weight loss, colon shortening, and histology scores, were suppressed significantly in the cohoused *Lrrc19* KO mice (Figures 2A–2C). In the AOM-DSS-induced colon cancer model, WT mice developed a high incidence of colon tumors in the distal to middle colon, whereas no tumors were found in cohoused *Lrrc19* KO mice treated with the same protocol (Figures 2D–2F). The colon cancer-associated markers proliferating cell nuclear antigen (PCNA) and COX2 were also difficult to detect in *Lrrc19* KO mice (Figure 2G). 5-Bromo-2-deoxyuridine (BrdU) experiments showed strong absorbance by colon epithelial cells of WT mice but not by those of *Lrrc19* KO mice (Figure 2G). These results indicated that LRRC19 functions in gut-related tumorigenesis. LRRC19 is difficult to detect in hematopoietic cells, but its expression is induced in inflammatory environments (unpublished data). Therefore, we assessed intrinsic hematopoietic cell functions in colitis generation and development. WT/WT chimeras (WT bone marrow [BM] donor cells transplanted into lethally irradiated WT recipients), but not cohoused KO/ko chimeras (*Lrrc19* KO BM donor cells transplanted into lethally irradiated *Lrrc19* KO recipients), responded effectively to DSS-induced colitis, further confirming the role of LRRC19 in colitis (data not shown). Notably, in *Lrrc19* KO mice that received WT bone marrow, the incidence and severity of colitis were significantly lower than those of WT mice transplanted with *Lrrc19* KO BM (Figures S1D–S1F), suggesting that epithelial but not hematopoietic expression of LRCC19 is necessary for the proinflammatory role of LRRC19 in DSS colitis. Together, these data indicate that LRRC19 deficiency impairs the inflammatory responses in gut tissues.

### LRRC19 Deficiency Reduces the Recruitment of Immune Cells

We next investigated the factor(s) that cause(s) the decreased inflammation in *Lrrc19* KO gut tissues. *Lrrc19* KO gut tissues and gut-associated lymphoid tissues from mice previously subjected to long-term observation were examined again. We found that *Lrrc19* KO mice had fewer and smaller gut tissue-associated lymph node PPs compared with cohoused WT mice (Figures 3A and 3B), indicating that fewer immune cells had accumulated in their gut immune systems. Because immune cells such as DCs, adaptive lymphocytes (including Th1 and Th17 cells), and immunoregulatory cells such as regulatory T (Treg) cells play critical roles in colitis and colitis-associated cancer (Nguyen et al., 2015), we examined the composition and absolute numbers of immune cells in the LPs, PPs, and MLNs of WT and *Lrrc19* KO mice. We found that the absolute numbers of $CD4^+$ T cells, $CD8^+$ T cells, DCs ($CD11c^+MHCII^+$ cells), macrophages (F4/$80^+MHCII^+$ cells), and immature myeloid cells ($CD11b^+Gr1^+$

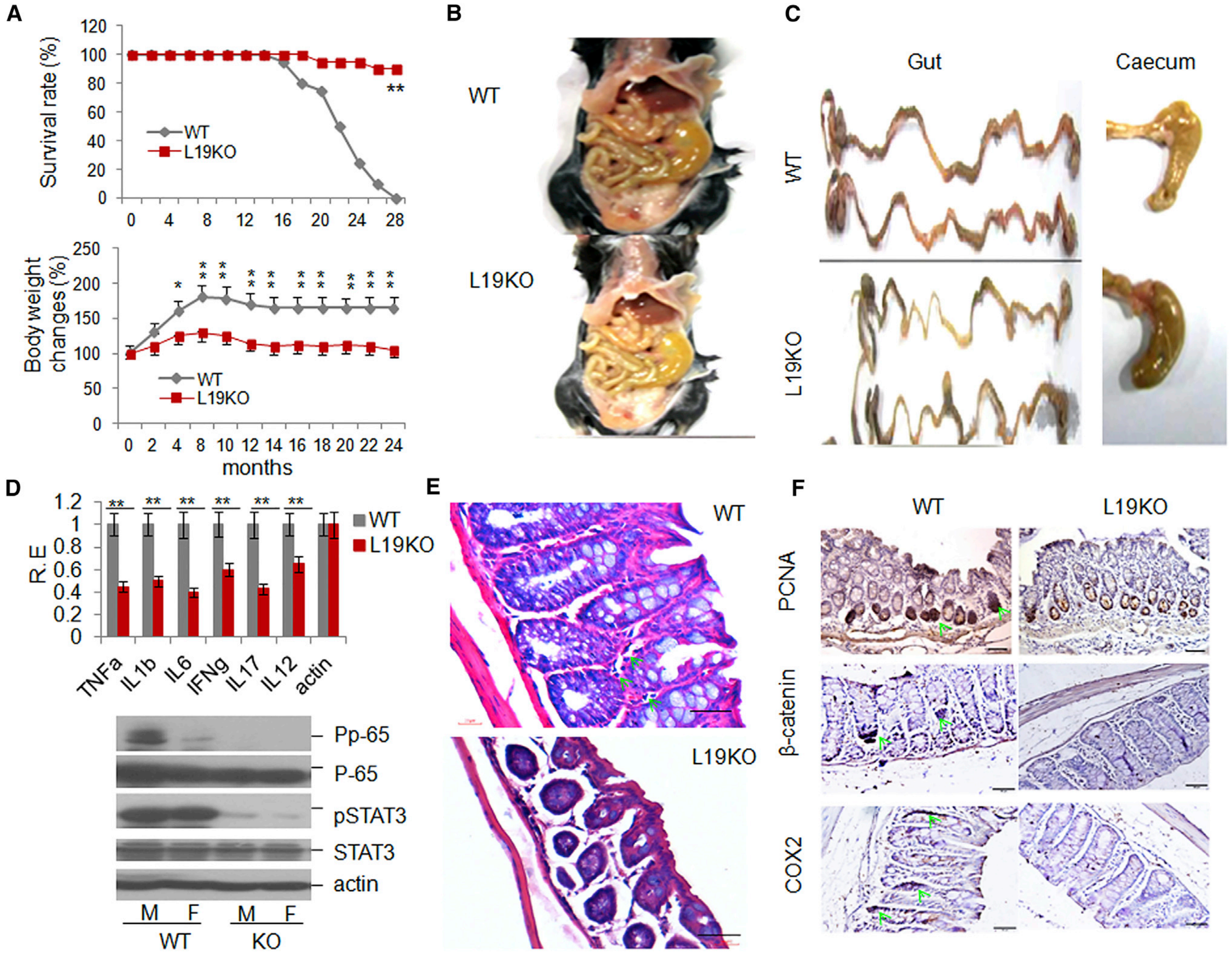


**Figure 1. LRRC19 Deficiency Inhibits the Occurrence and Development of Gut Inflammation**

(A) Survival rates (top) and changes in body weight (bottom) of WT (WT, n = 24 [female, 12; male, 12]) and *Lrrc19* KO (L19KO, n = 24 [female, 12; male, 12]) mice on standard chow.

(B and C) Morphology of gut and cecum from representative WT (B) and *Lrrc19* KO (C) mice after eating standard chow for 2 years.

(D) qRT-PCR analyses (top) of TNF-α, IL-1β (IL1b), IL-6, IFNγ (IFNg), IL-17, and IL-12 and immunoblot (bottom) of pp-65 and pSTAT3 in colon tissues of WT (n = 6) and *Lrrc19* KO (n = 6) mice after standard chow for 2 years. RE, relative expression; M, male; F, female.

(E) H&E staining of representative WT and *Lrrc19* KO mouse colons after standard chow for 2 years. The green arrow indicates inflammatory response cells. Scale bars, 40 μm.

(F) Immunostaining of PCNA, β-catenin, and COX2 in colon tissues of representative WT and *Lrrc19* KO mice. The green arrows indicate PCNA, β-catenin, and COX2. Scale bars, 40 μm.

*$p < 0.05$, **$p < 0.01$ (Wilcoxon's test in A [top], ANOVA in A [bottom], t test in D; mean ± SD). See also Figure S1.

cells) were much lower in the LPs of *Lrrc19* KO mice than in those of WT mice (Figure 3C). The smaller numbers of innate and adaptive immune cells in *Lrrc19* KO mice were confirmed with immunostaining (Figure S2). The absolute numbers of CD103$^+$CD11b$^-$ (CD103$^+$ DCs), CD103$^+$CD11b$^+$ (double-positive [DP] DCs), and CD103$^-$CD11b$^+$ (CD11b$^+$ DCs) subsets were also lower in the PPs of *Lrrc19* KO mice compared with those of cohoused WT mice (Figure 3D). However, the proportion of the CD11c$^+$CD103$^+$ DC subpopulation, which plays a critical role in maintaining gut tolerance (Benson et al., 2007; Coombes et al., 2007), did not decrease but, rather, increased in the PPs and MLNs of *Lrrc19* KO mice (Figure S3B). The proportion of CD11b$^+$ DCs (CD11C$^+$CD103$^+$CD11b$^+$ and CD11c$^+$CD11b$^+$ cells) was lower in *Lrrc19* KO mice than in WT mice (Figure S3B), indicating that there were fewer hematopoietic CD11b$^+$ DCs in *Lrrc19* KO mice than in WT mice. Importantly, the absolute numbers of adaptive lymphocytic subsets (Th1 and Th17 cells) in the PPs and MLNs of *Lrrc19* KO mice, which may promote inflammation responses, were less compared with those of WT mice (Figures 3E and 3F). Th17 cell frequency in the PPs and MLNs was also reduced markedly in *Lrrc19* KO mice, whereas the Treg (Foxp3$^+$) cell proportion, which may suppress inflammation

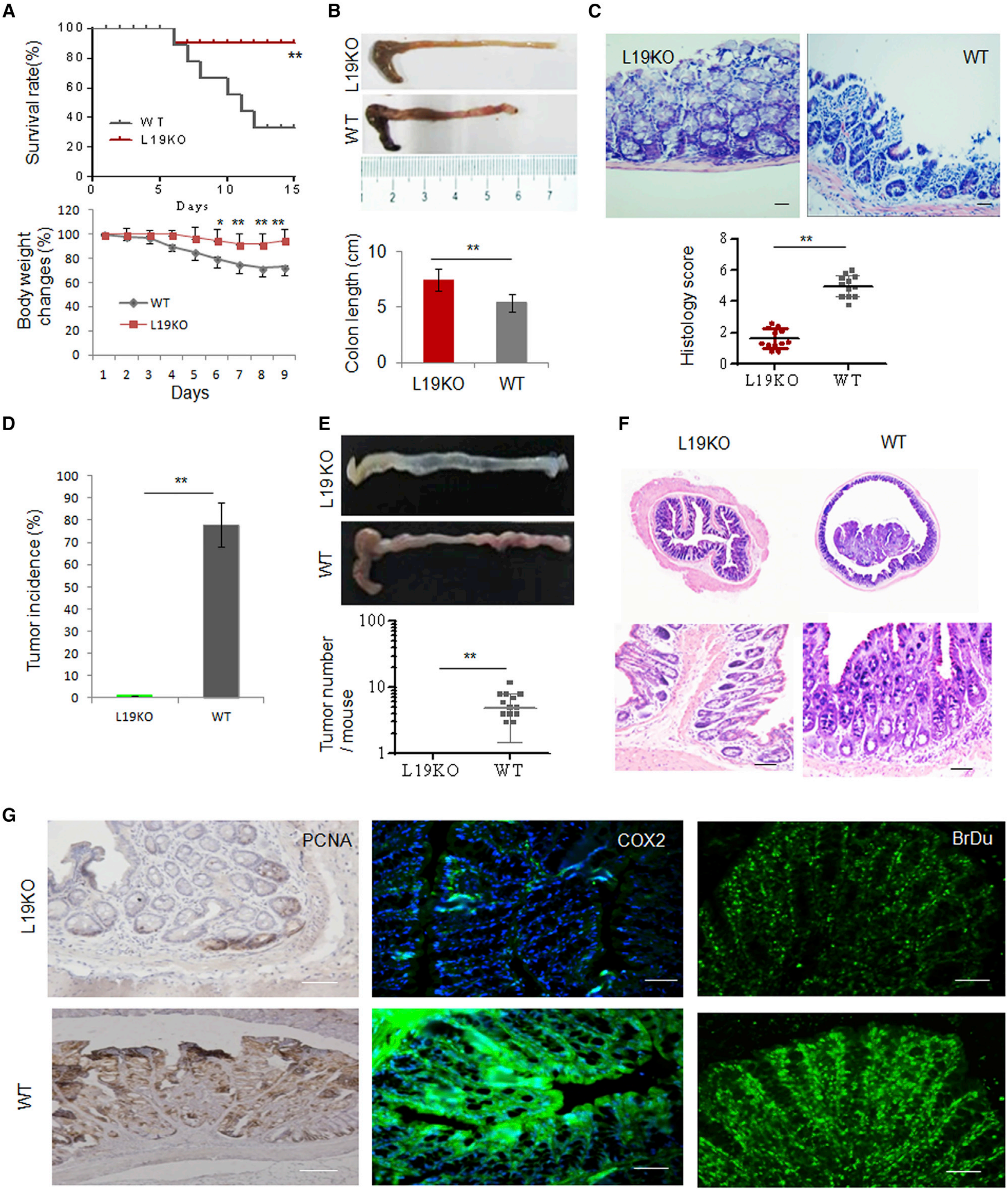


**Figure 2.** ***Lrrc19*** **KO Mice Are Highly Resistant to DSS-Induced Colitis**

(A) Survival (top) and body weight (bottom) were monitored after the start of DSS. WT (n = 18, male) and *Lrrc19* KO (n = 18, male) mice were fed a 2% DSS solution in drinking water for 7 days and then switched to regular drinking water.

(B) Length of WT and *Lrrc19* KO colon tissues. Mice were sacrificed on day 7 after the start of DSS, and colon length was measured.

(legend continued on next page)

responses, was increased (Figure S3D). Notably, the reduced number of T cells and the reduction in the Th17 subpopulation in *Lrrc19* KO mice were limited to the gut tissue. *Lrrc19* KO and WT mice did not differ in the numbers of DCs or T cells in their spleens. No similar phenomena were found in *Tlr4* KO, *Tlr2* KO, or *Myd88* KO mice (data not shown). These data suggest that the decreased number of immune cells and their subsets may be responsible for the reduced gut inflammation in *Lrrc19* KO mice.

## Reduced Chemokine Levels in *Lrrc19* KO Gut Tissues Are Responsible for Fewer Gut Immune Cells

We next investigated the cause of the decreased number of immune cells in *Lrrc19* KO gut tissues. Previous studies have shown that chemokines play a critical role in the recruitment and retention of immune cells. However, for chemokines such as MCP-1/CCL2, which plays roles in recruiting immune cells to gut tissues (Popivanova et al., 2009), there was no remarkable difference in CCL2 expression between cohoused WT and *Lrrc19* KO mice, especially in the levels of transcription (Figure 4B). To identify chemokines involved in recruiting immune cells in gut tissues, we used a microarray to analyze the differential expression of genes in the gut epithelial cells of cohoused WT and *Lrrc19* KO mice. The expression of several chemokines, including CCL6, CCL9, CXCL9, and CXCL10, was reduced significantly in *Lrrc19* KO mice (Figure 4A; http://www.ncbi.nlm.nih.gov/geo/query/acc.cgi?acc=GSE62487). The reduced levels of chemokine expression in LRRC19-deficient epithelial cells were confirmed further with qRT-PCR, immunoblotting, and immunostaining (Figure 4B; data not shown). These chemokines play a critical role in recruiting innate and adaptive immune cells, including DCs, macrophages, and $CD4^+$ and $CD8^+$ T cells (Asensio et al., 1999; Coelho et al., 2007; Harris et al., 2012; Núñez et al., 2010; Zhao et al., 2003). Indeed, administration of CCL6-, CCL9-, CXCL9-, and CXCL10-expressing adenoviral complexes could not only rescue the innate and adaptive immune system in *Lrrc19* KO mice (Figures 4C–4E) but also promoted the accumulation of innate and adaptive immune cells, including $CD4^+$ and $CD8^+$ T cells, DCs, macrophages, and immature myeloid cells in the LP (Figure S4B) in WT mice. The MLN sizes were also significantly larger in mice injected with chemokine-expressing adenoviral complexes (Figure S4C). These extra chemokines also increased the sensitivity to DSS-mediated colitis in *Lrrc19* KO mice (Figure 4E) and in WT mice (Figures S4D and S4E). These data, together, suggest that LRRC19-associated CCL6, CCL9, CXCL9, and CXCL10 in gut epithelial cells play a critical role in the recruitment of gut immune cells.

## Altered Gut Microbiota in *Lrrc19* KO Mice Affects Chemokine Expression

We next addressed factor(s) that potentially affect chemokine expression in gut tissues of *Lrrc19* KO mice. Previous studies have suggested that gut commensal microbiota composition is important for establishment of the intestinal immune system. The fewer immune cells in the *Lrrc19* KO mice may, therefore, be attributable to their altered gut microbiota. We first analyzed fecal microbiotas by pyrosequencing 16S rRNAs in WT and *Lrrc19* KO mice. The animal husbandry, parental genotypes, and environmental influences were controlled carefully. The *Lrrc19* KO and cohoused WT mice shared the same bacterial phyla, but, among their microbiotas, the proportions of Firmicutes and Bacteroidetes were significantly different between *Lrrc19* KO (29% Firmicutes and 55% Bacteroidetes) and WT mice (59% Firmicutes and 34% Bacteroidetes). However, there was a marked increase in Bacteroidetes and a marked reduction in Firmicutes in *Lrrc19* KO mice compared with WT mice (Figures 5A and 5B; Table S1). These data were confirmed with 16S rRNA qPCR (Figure 5C). The relative abundances of bacterial phyla in WT mice (Figures 5A and 5B; Table S1) were consistent with the data of other studies (Ley et al., 2006; Turnbaugh et al., 2009). Further analyses showed far fewer *Clostridium* bacteria in the colons or *Lactobacillus* bacteria in the intestines of *Lrrc19* KO compared with WT mice (Table S1). Therefore, we next used a GF mouse model to demonstrate that the altered gut microbiota in *Lrrc19* KO mice is a factor in their reduced levels of chemokines. We first compared the chemokine levels in the intestinal tissues of *WT* and GF mice. The expression of CCL6, CCL9, CXCL9, and CXCL10 was much lower, or even barely detectable, in the intestinal tissues of GF mice compared with WT mice (Figure 5D). However, the expression of these chemokines was remarkably higher in GF mice transplanted with WT mouse feces compared with that in GF mice transplanted with *Lrrc19* KO mouse feces (Figures 5E and 5F), suggesting that the altered gut microbiota in *Lrrc19* KO mice affects chemokine expression in their gut epithelial cells.

## REGs Are Involved in the Alteration of the Gut Microbiota in *Lrrc19* KO Mice

We next addressed the causes of the altered gut microbiota in *Lrrc19* KO mice. To investigate this, we again analyzed the microarray data from gut epithelial cells of WT and *Lrrc19* KO mice. The expression of REGs, including REG3$\alpha$, REG3$\beta$, REG3$\gamma$, and REG4, was significantly lower in the gut epithelial cells of *Lrrc19* KO mice compared with the gut epithelial cells of WT mice (Figure 6A; http://www.ncbi.nlm.nih.gov/geo/query/acc.cgi?acc=GSE62487). These results were confirmed with qRT-PCR, immunoblotting, and immunostaining (Figure 6B;

(C) H&E staining and histological scores of representative distal colon samples from WT and *Lrrc19* KO mice on day 7 after the start of DSS. Histological scores were assessed according to the methods described in the Supplemental Experimental Procedures.

(D) Incidence of colon carcinoma in WT (n = 18, male) and *Lrrc19* KO (n = 18, male) mice after AOM-2% DSS treatment for 3 months.

(E) Morphology and tumor numbers of colon carcinoma in WT and *Lrrc19* KO mice after AOM-2% DSS treatment for 3 months.

(F) Histopathological changes in representative distal colon samples from *Lrrc19* KO and WT mice after staining with H&E.

(G) Immunostaining of PCNA, COX2, and BrdU in colon tissues of representative WT and *Lrrc19* KO mice. Colon samples from *Lrrc19* KO and WT mice were stained by anti-PCNA or anti-COX2 antibodies. For the BrdU assay, mice were injected intraperitoneally with BrdU, and the colon sections were stained by anti-BrdU antibodies after 4 hr. Brown, PCNA; green, COX2 or BrdU.

Scale bars, 40 $\mu$m. *p < 0.05, **p < 0.01 (Wilcoxon's test in A [top], ANOVA in A [bottom], t test in B and D, mean ± SD; Mann-Whitney *U* test in C and E).

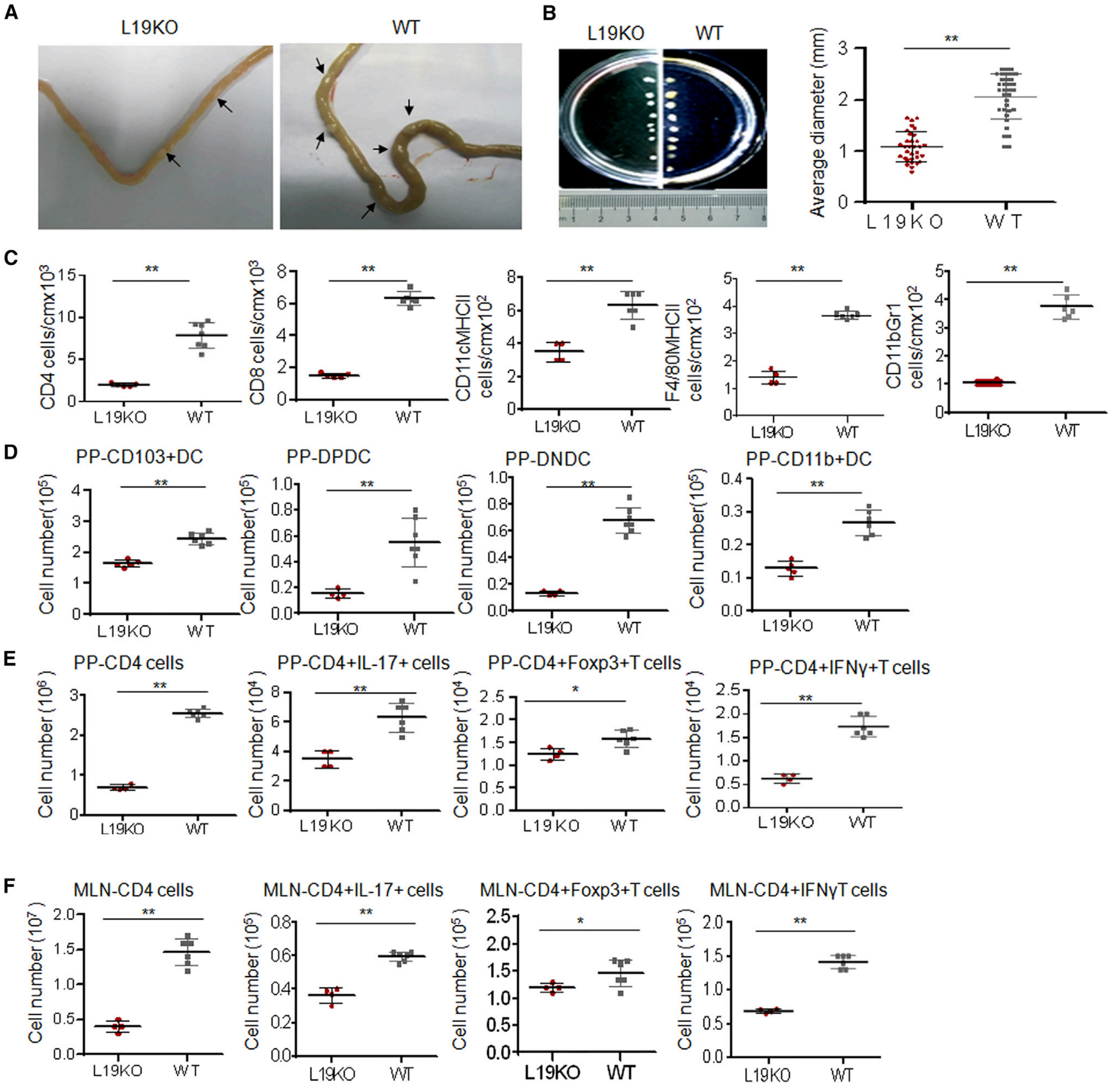


**Figure 3. LRRC19 Deficiency Affects Maturation of Gut-Associated Lymphoid Tissues**

(A and B) Visible PP number (A) and sizes (B) in the guts of WT and *Lrrc19* KO mice.

(C) Absolute numbers of CD4$^+$T cells, CD8$^+$ T cells, CD11c$^+$MHCII$^+$ cells, F4/80$^+$MHCII$^+$ cells, and CD11b$^+$Gr1$^+$ cells in colon tissues. The absolute numbers were standardized by calculating the numbers per l cm of colon.

(D) Numbers of CD11c$^+$CD103$^+$CD11b$^-$DCs (pp-CD103$^+$DC), CD11C$^+$CD103$^+$CD11b$^+$ DCs (PP-DP-DC), CD11C$^+$CD11b$^-$CD103$^-$DCs (PP-DNDC), and CD11C$^+$CD11b$^+$ (PP-CD11b$^+$DC) in PPs as assessed by flow cytometry analysis.

(E and F) Number of CD4$^+$, CD8$^+$, CD4$^+$Foxp3$^+$, CD4$^+$IL-17$^+$, and CD4$^+$IFN$\gamma^+$ T cells in PPs (E) and MLNs (F). Cell numbers in age- and sex-matched WT (n = 6) and *Lrrc19* KO (n = 6) mice were compared.

*p < 0.05, **p < 0.01 (Mann-Whitney *U* test). The data are representative of three independent experiments. See also Figures S2 and S3.

data not shown). Because the members of the REG family have the capacity to specifically kill Gram-positive bacteria (Brandl et al., 2007; Mukherjee et al., 2014; Vaishnava et al., 2011) and alter the composition of the gut microbiota (Figure 6C), the reduced expression of REGs in LRRC19 KO mice may explain their altered gut microbiota.

A critical question is whether the reduced expression of CCL6, CCL9, CXCL9, and CXCL10 is associated with REG-mediated

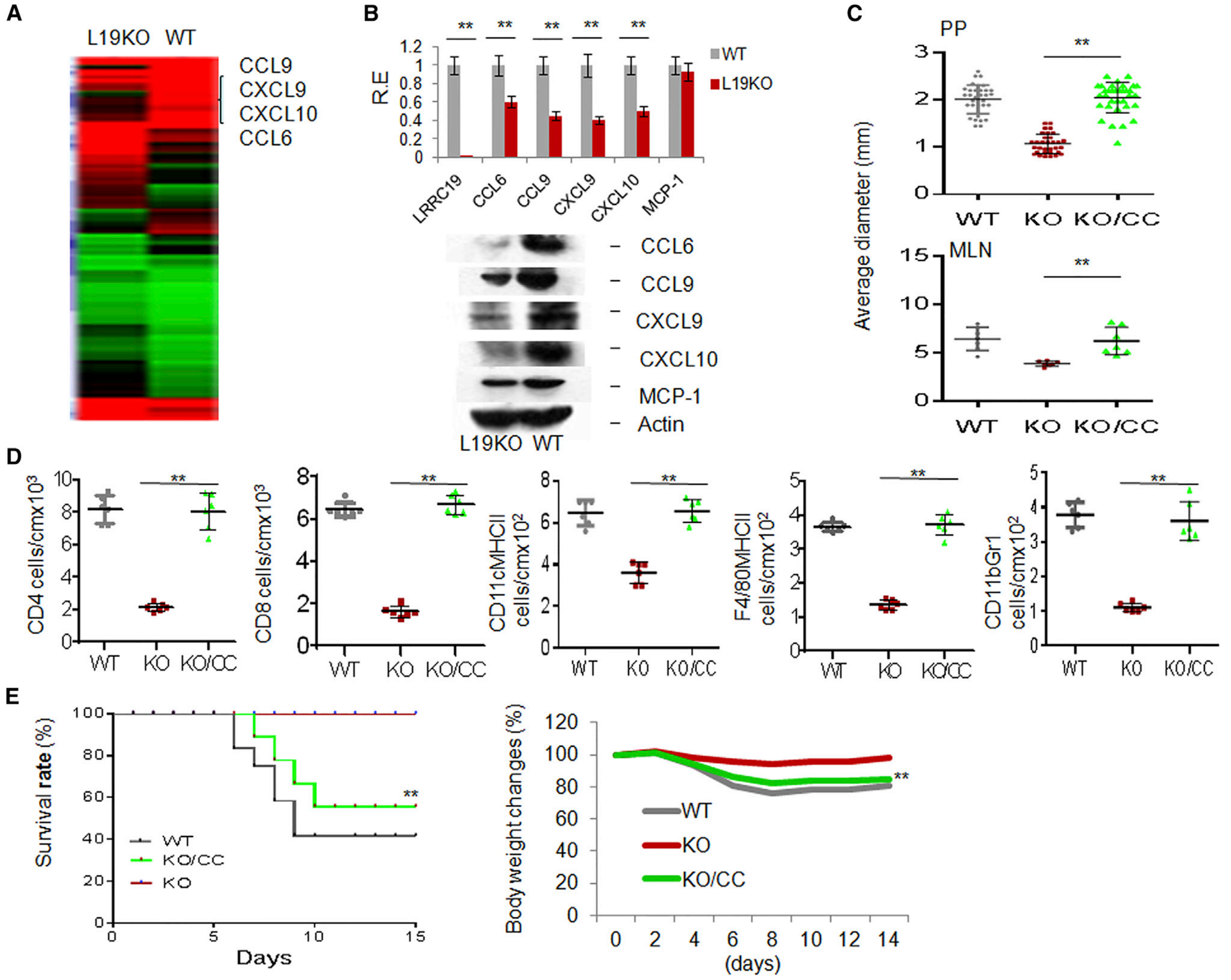


**Figure 4. LRRC19 Deficiency Reduces the Expression of Chemokines CCL6, CCL9, CXCL9, and CXCL10**

(A) Microarray analyses of gene expression in WT and *Lrrc19* KO mice given standard chow.

(B) qRT-PCR (top) and immunoblot (bottom) of CCL6, CCL9, CXCL9, and CXCL10 in WT and *Lrrc19* KO colon epithelial cells.

(C) Size of visible PP and MLN in *L19* KO mice with (KO/CC) or without (KO) CCL6-, CCL9-, CXCL9-, and CXCL10-expressing adenovirus injection.

(D) Absolute number of CD4$^+$ and CD8$^+$ T cells, CD11C$^+$MHCII$^+$ DCs, F4/80MHCII, and CD11b$^+$Gr1$^+$ cells in colon tissue of *L19* KO mice with or without (control adenovirus only) chemokine adenovirus injection as assessed by flow cytometric analysis. CC, CCL6-, CCL9-, CXCL9-, and CXCL10-expressing adenovirus complexes; WT, cell numbers from the colon tissues of WT mice.

(E) Survival (right) and body weight (left) were monitored until day 14 after the start of DSS. *L19 KO* mice with (n = 18) or without (n = 18) CCL6, CCL9, CXCL9, and CXCL10 adenovirus injection were fed a 2% DSS solution in drinking water for 7 days and then switched to regular drinking water.

*p < 0.05, **p < 0.01 (t test in B, mean ± SD; Mann-Whitney *U* test in C and D; Wilcoxon's test in E (right); ANOVA in E (left). See also Figure S4.

gut microbiotas in *Lrrc19* KO mice. To demonstrate this, we first administrated REG adenoviruses into *Lrrc19* KO mice. We found that the expression of chemokines was much higher in the intestinal tissues of *Lrrc19* KO mice with REG adenovirus administration compared with uninjected mice (Figure 6D), indicating that the expression of chemokines is related to REG levels in gut tissues. We next addressed whether REG-mediated chemokines were associated with the gut microbiota of mice administrated REG adenoviruses. When gut microbiotas of *Lrrc19* KO mice with REG adenoviruses were transplanted into *Lrrc19* KO mice, the expression of chemokines was much higher than in *Lrrc19* KO mice transplanted with control feces (Figure 6E), indicating that the altered gut microbiota after administration of REG adenoviruses affects chemokine expression in their gut epithelial cells. Taken together, these results suggest that the REG-mediated gut commensal microbiota modulates the expression of chemokines.

## Gut *Lactobacillus* Directly Regulates the Expression of REGs through LRRC19-Mediated Activation of NF-κB

Because reduced REGs are responsible for the altered microbiota, which caused the decreased chemokine levels in *Lrrc19*

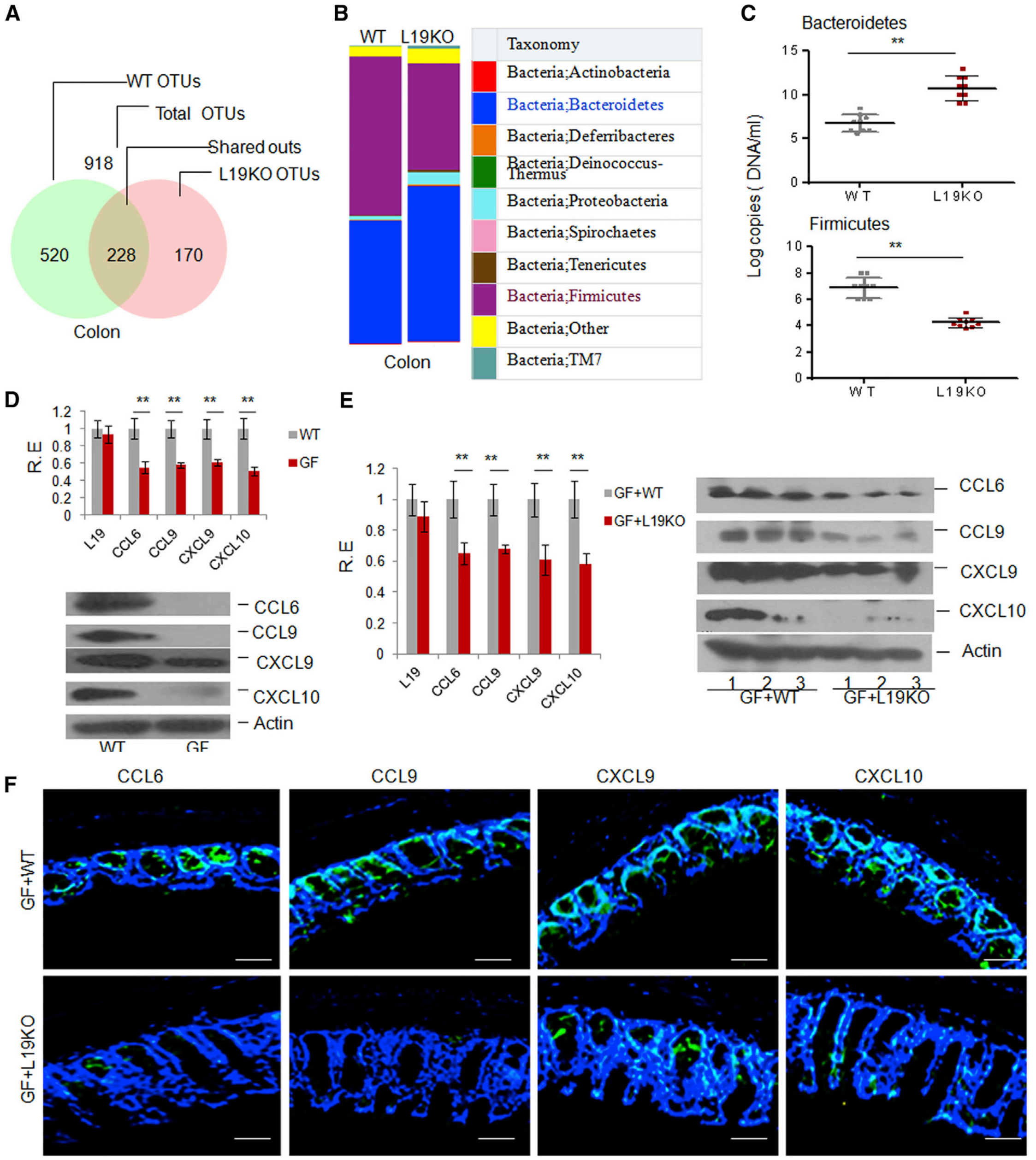


**Figure 5. The LRRC19-Associated Gut Microbiota Modulates the Expression of Chemokines**

(A and B) 16S rRNA analyses of gut microbiota of WT and *Lrrc19* KO mice. The samples were clustered at the operational taxonomic unit (OTU) (A) and phylum (B) levels using the sample OTU and sample phylum count matrices, respectively.

(C) qRT-PCR of gut microbiota. The abundance of bacteria in WT and *Lrrc19* KO mice was measured as bacterium-specific 16S rRNA copy numbers by qPCR analysis of fecal pellets. Standard curves were prepared from serial dilution of *E. coli* genomic 16S rRNA extracted in the same manner as above.

(D) qRT-PCR and immunoblot of CCL6, CCL9, CXCL9, and CXCL10 in WT and GF mice.

(E) qRT-PCR and immunoblot of CCL6, CCL9, CXCL9, and CXCL10 in GF+WT and GF+L19KO mice. GF+WT, GF mice transplanted with the microbiota from the feces of WT mice; GF+L19KO, GF mice transplanted with microbiota from the feces of *Lrrc19* KO mice.

(F) Immunostaining of CCL6, CCL9, CXCL9, and CXCL10 in GF+WT and GF+L19KO mice. Scale bars, 40 μm.

*$p < 0.05$, **$p < 0.01$ (Mann-Whitney *U* test in C; t test in D and E, mean ± SD). See also Table S1.

KO mice, we next explored how LRRC19 affects the expression of REGs. LRRC19, as a potential bacterium recognition receptor, may be activated by gut bacteria to regulate the expression of REGs. The genus *Lactobacillus* was decreased remarkably in Lrrc19 KO mice (Table S1). Therefore, the reduced expression of the REGs in *Lrrc19* KO mice may be caused by the decrease in *Lactobacillus*. After screening the isolated gut commensal bacteria, we identified the strain of *Lactobacillus* with the highest

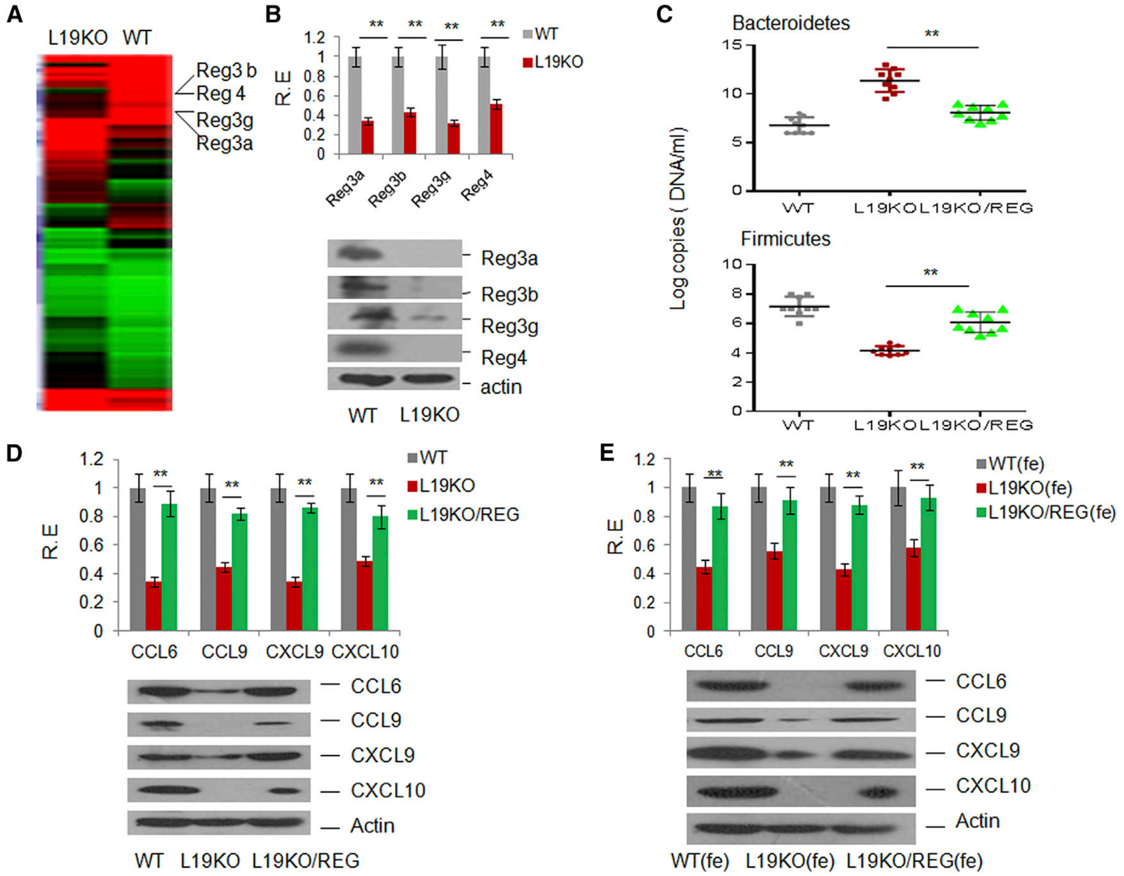


**Figure 6. Expression of Chemokines Is Associated with the REG-Mediated Microbiota**

(A) Microarray analysis of gene expression in gut tissues of WT and *Lrrc19* KO mice.

(B) qRT-PCR (top) and immunoblot (bottom) of REG3α, REG3β, REG3γ, and REG4 in WT and *Lrrc19* KO colon tissues.

(C) qRT-PCR of gut microbiotas. The abundance of bacteria in *L19* KO mice with (KO/REGs) or without (KO, control adenoviruses only) REG3α-, REG3β-, REG3γ-, and REG4-expressing adenovirus injection was measured as 16S rRNA copy numbers by qPCR analysis of fecal pellets. Standard curves were prepared from serial dilution of *E. coli* genomic 16S rRNA extracted in the same manner as above.

(D) qRT-PCR and immunoblot of CCL6, CCL9, CXCL9, and CXCL10 in *L19* KO mice with or without (control adenoviruses only) REG adenovirus injection. Adenovirus-Reg3α, -Reg3β, -Reg3γ, and -Reg4 complexes (REG) or control adenovirus were injected intraperitoneally once per week, three times.

(E) qRT-PCR and immunoblot of CCL6, CCL9, CXCL9, and CXCL10 in feces-transplanted mice. Mice were first treated using pan-antibiotics (1 g/l ampicillin, Sigma), 0.5 g/l vancomycin, 1 g/l neomycin sulfate, and 1 g/l metronidazole) in drinking water for 4 weeks and were then transplanted with microbiotas from feces. WT(fe), *Lrrc19* KO mice transplanted with the microbiota from the feces of WT mice; L19KO(fe), *Lrrc19* KO mice transplanted with the microbiota from the feces of *Lrrc19* KO mice with control adenovirus injection; L19KO/REG(fe), *Lrrc19* KO mice transplanted with the microbiota from the feces of REG adenovirus-administered *Lrrc19* KO mice. Expression of CCL6, CCL9, CXCL9, and CXCL10 was analyzed 3 days after transplantation.

*p < 0.05, **p < 0.01 (Mann-Whitney *U* test in C; t test in D and E, mean ± SD). See also Figures S5, S6, and S7.

similarity to *Lactobacillus taiwanensis* strain BCRC 17755, *Lactobacillus* NK6 (colony 6) (Figure S5). This strain could induce the expression of REGs in gut epithelial cells (Figures S6A and S6B). Notably, lactobacilli could activate the intracellular NF-κB signaling pathway in LRRC19- but not ΔLRRC19-transfected (absence of an extracellular region) HEK293T cells (Figure S6C). All of these data suggest that LRRC19 may directly regulate the expression of REGs.

We next dissected the molecular pathways by which LRRC19 signaling regulates the expression of REG family proteins. LRRC19 deficiency affected the phosphorylation of p65, p38, and c-Jun N-terminal kinase (JNK) in response to *Lactobacillus* (Figure S7A), indicating that LRRC19 activates multiple signaling pathways. Consistent with previous findings (Su et al., 2014), there was more K63-linked ubiquitin on the TRAF6 from WT gut epithelial cells than on TRAF6 from *Lrrc19* KO cells (Figure S7B). The ubiquitination of TRAF2 was also inhibited markedly in WT gut epithelial cells, whereas the ubiquitination of TRAF2 in *Lrrc19* KO cells was increased greatly in response to *Lactobacillus* NK6 (Figure S7C). Because TRAF2 and TRAF6 are critical adaptor molecules of NF-κB signaling pathways, we also examined the effect of NF-κB deficiency on the expression of REG family members. The expression of REG family members such as REG3α, REG3β, REG3γ, and REG4 was lower in *NF-κB* KO mice than in WT mice (Figure S7D). *NF-κB* KO mouse gut epithelial cells had a highly reduced response to *Lactobacillus*

NK6 (Figure S7E), implying that LRRC19 mediates the expression of REG3 family members through NF-κB signaling pathways. This is consistent with previous findings (Vaishnava et al., 2011). These results suggest that LRRC19 mediates the expression of REG proteins through the TRAF2- and TRAF6-mediated NF-κB signaling pathways.

## Exogenous LRRC19 Promotes the Occurrence and Development of Colitis

Finally, we examined the importance of LRRC19 in promoting the recruitment of immune cells and intestinal inflammation. LRRC19-expressing adenovirus was injected intraperitoneally three times per week, with demonstrable infection (Figure 7A). This treatment increased the sensitivity of mice to DSS-mediated colitis (Figure 7B) and AOM-DSS-induced colon cancer (Figure 7C). The administration of LRRC19-expressing adenovirus also increased the infiltration of different immune cells into the gut tissues (Figure 7D) and altered the immune cell proportions in that the $CD103^{+}CD11b^{+}$ cell population in the LP increased dramatically in mice administered LRRC19-expressing adenovirus and the $Th1^{+}$ ($IFN\gamma^{+}$) and $Th17^{+}$ cell subsets in the PPs and MLNs also increased (data not shown). qRT-PCR and immunoblot analyses showed that CCL6, CCL9, CXCL9, and CXCL10 expression in mice administered LRRC19-expressing adenovirus was also higher than in control mice (Figures 7E and 7G). Expression of the REG family proteins REG3α, REG3β, REG3γ, and REG4 was higher in adenovirus-treated mice than in mice injected with the control vector (Figures 7F and 7G). All of these data confirm the critical role of LRRC19 in the recruitment of immune cells and intestinal inflammation.

## DISCUSSION

Gut epithelial cells express multiple pattern recognition receptors, such as TLRs and Nod-like receptors, that play important roles in eliminating pathogenic microorganisms. However, the expression levels of these TLRs are too low under normal physiological conditions to produce a responsive to TLR stimuli (Melmed et al., 2003). Therefore, it is not really clear what type of receptors plays a critical role in the establishment and maturation of the gut immune system. We found that LRRC19, which does not contain a cytoplasmic Toll/interleukin 1 receptor domain, determines the recruitment of immune cells and intestinal inflammation under normal physiological conditions. Unlike the pathogen-recognizing receptors, LRRC19 is highly expressed on gut epithelial cells and directly mediates the TRAF2- and TRAF6 NF-κB signaling pathways in gut epithelial cells.

Mining the microbiota for bacterial strains that are responsible for shaping the gut immune system is a formidable combinatorial problem. Although some progress has been made in identifying gut microbe species that preferentially stimulate a specific program of immune maturation, the gut-specific commensal microbiota for maintaining the maturation of the whole gut immune system is largely unknown. We found that gut commensal microbes of the genus *Lactobacillus* are involved in this process. Although the particular bacterial products are unknown, we demonstrated that *Lactobacillus* may promote the expression of REG3α, REG3β, and REG3γ through LRRC19-mediated TRAF2- and TRAF6 NF-κB signaling pathways in gut epithelial cells. This may affect the recruitment of immune cells and intestinal inflammation by gut microbiota-mediated chemokines. *Lactobacillus* are Gram-positive bacteria with the cell wall component peptidoglycan. Recognition of peptidoglycan is important in initiating and shaping the immune response under both homeostatic and infection conditions (Sorbara and Philpott, 2011). Indeed, four other secreted peptidoglycan recognition proteins, PGLYRP 1-4, as well as two intracellular sensors of peptidoglycan, Nod1 and Nod2, also have important roles in shaping mammalian immune responses (Sorbara and Philpott, 2011).

Our study establishes an LRRC19-based regulatory axis that may promote the recruitment of immune cells and intestinal inflammation. Chemokines CCL6, CCL9, CXCL9, and CXCL10 may directly recruit immune cells into the gut tissues, whereas REG family proteins, including REG3α, REG3β, REG3γ, and REG4, play critical roles by affecting the composition of the gut microbiota, which may modulate chemokine expression. Commensal microbes of the genus *Lactobacillus* are essential for the establishment of the gut immune system by activating the LRRC19-mediated signaling pathway, which may induce the expression of REG family proteins such as REG3α, REG3β, and REG3γ. These results represent an important advance in understanding how gut commensal microbes exert their effects to promote the recruitment of immune cells and intestinal inflammation through the microbe receptors expressed on gut epithelial cells. This will be invaluable when designing therapeutic strategies for colitis and colitis-associated diseases such as colon carcinoma.

## EXPERIMENTAL PROCEDURES

### Mice

Four-to six-week-old male or female C57BL/6 and GFP transgenic C57BL/6 mice were obtained from the Beijing Animal Center. *Lrrc19* KO mice were generated by us (Su et al., 2014). *Tlr2* KO mice were obtained from the Nanjing Animal Center. *Nf-κb* KO ($p50^{-/-}$) mice were provided by Prof. Zhexiong Lian (University of Science and Technology of China). Germ-free mice were generated by the Third Military Medical University and were bred and maintained in sterile Trexler-type isolators. All procedures were conducted according to the Institutional Animal Care and Use Committee of the Model Animal Research Center. Animal experiments were approved by the Animal Ethics Committee of Nankai University.

### Preparation of Specific Experimental Mice

Chimeric mice were generated according to our previously published method (Su et al., 2014). Briefly, 6-week-old *Lrrc19* KO and WT GFP transgenic C57BL/6 mice were lethally irradiated with 9 Gy of total body irradiation. BM cells were obtained from the femora of donor mice and collected in RPMI 1640 medium containing 100 U/ml penicillin/streptomycin. Irradiated recipient mice were injected with 200 μl of the appropriate cell suspension via the tail vein. The recipient mice were maintained in a sterile facility for 8 weeks to allow for complete engraftment with donor bone marrow. To assay bone marrow reconstitution, spleens were harvested from chimeric mice, and single-cell suspensions of splenocytes were prepared in PBS and then analyzed by flow cytometry to detect GFP+ spleen cells and to determine the donor/recipient chimerism of the hematopoietic compartment.

For microbiota transplantation mice, cecal contents were pooled from five mice. Cecal contents (150 mg) were collected in an anaerobic chamber, suspended in 1 ml PBS, and immediately administered intragastrically

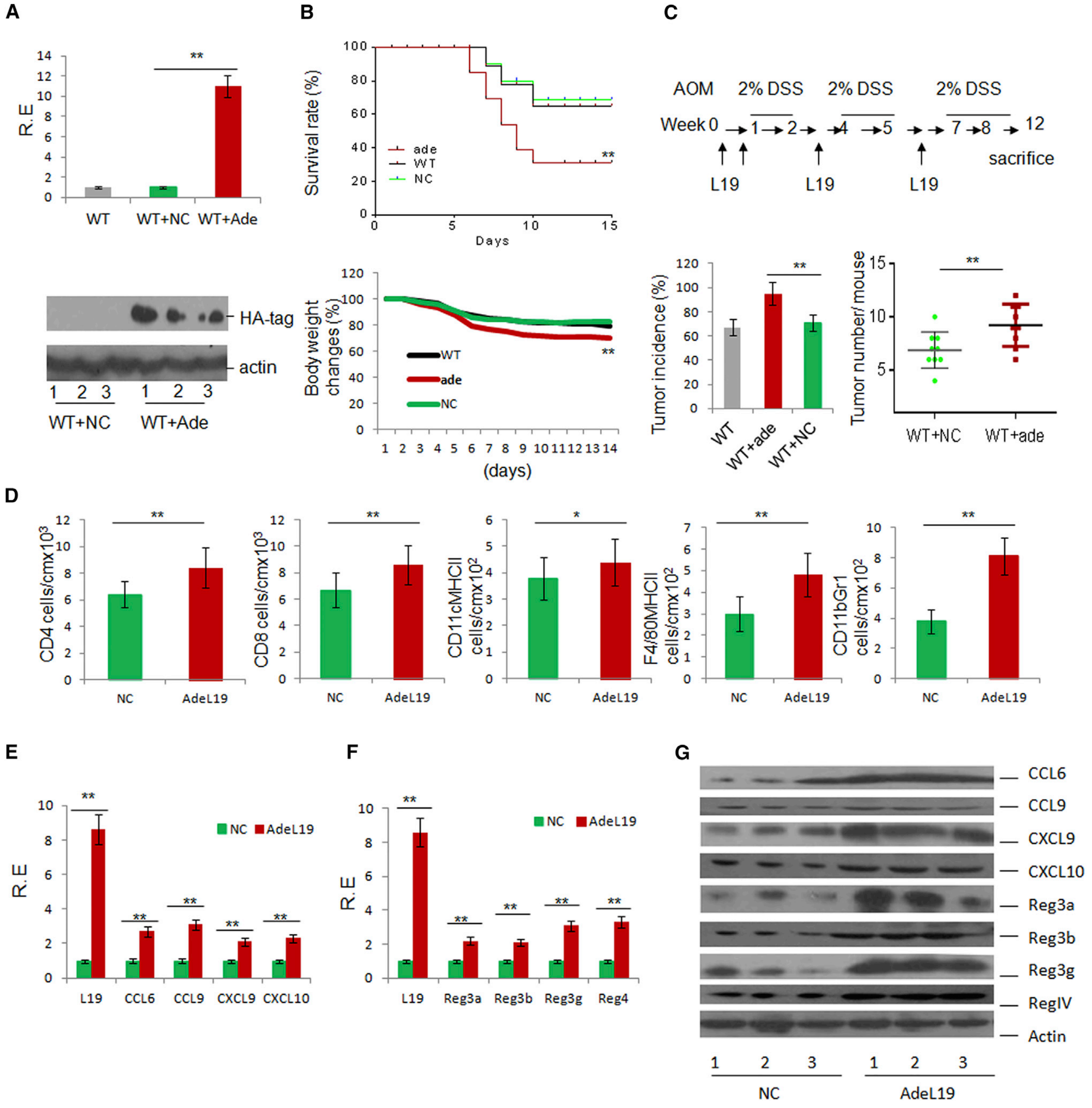


**Figure 7. Exogenous LRRC19 Promotes the Occurrence and Development of Gut Inflammation**

(A) qRT-PCR (top) and immunoblot (bottom) of LRRC19 in LRRC19 adenovirus- (WT+ade) or control adenovirus (WT+NC)-injected mice. Colon tissues were lysed and analyzed for LRRC19 expression by anti-hemagglutinin (HA), with which LRRC19 was tagged in adenoviruses.

(B) Survival (top) and body weight (bottom) were monitored until day 14 after the start of DSS. Mice with (Ade) and without LRRC19 (NC) adenovirus injection (n = 16, male) were fed a 2% DSS solution in drinking water for 7 days and then switched to regular drinking water.

(C) Experimental design (top) and incidence and tumor numbers (bottom) of colon carcinoma in LRRC19 adenovirus- (WT+ade) (n = 16) or control adenovirus-administered (WT+NC) mice (n = 16) after AOM-2% DSS treatment for 3 months.

(D) Absolute number of $CD4^+$T cells, $CD8^+$ T cells, $CD11C^+MHCII^+$ DCs, $F4/80^+MHCII^+$, and $CD11b^+Gr1^+$ cells in colon tissues of LRRC19 adenovirus- (AdeL19) or control adenovirus (NC)-injected mice (n = 6) as assessed by flow cytometry. The absolute numbers were standardized by calculating the numbers per l cm of colon.

(E, F, and G) qRT-PCR (E and F) and immunoblot (G) of CCL6, CCL9, CXCL9, and CXCL10 and REG3α, REG3β, REG3γ, and REG4 in LRRC19 adenovirus (AdeL19)- or control adenovirus (NC)-injected mice (n = 6). Lanes 1–3 in (G) are representatives of six mice.

*$p < 0.05$, **$p < 0.01$ (Wilcoxon's test in B [top]; ANOVA in B [bottom]; t test in A, C, D, E, and F, mean ± SD; Mann-Whitney *U* test in C for tumor number).

(0.1 ml/mouse) to sterilely packed, 6- to 7-week-old germ-free mice or pan-antibiotic-treated mice. The *Lactobacillus* NK colony was selected and cultured in *Lactobacillus*-selective medium, suspended in PBS, and immediately administered intragastrically (1 × $10^9$ colony-forming units [CFUs]/mouse) to sterilely packed, 6- to7-week-old mice three times per week for 4 weeks.

For mice expressing exogenous LRRC19; chemokine or REG; adenovirus-LRRC19, -CCL6, -CCL9, -CXCL9, and -CXCL10 complexes (CC); or adenovirus-Reg3α, -Reg3β, -Reg3γ, and –Reg4 complexes (REG) were injected intraperitoneally at the indicated time. The expression of LRRC19, CCL6, CCL9, CXCL9, and CXCL10 or Reg3α, Reg3β, Reg3γ, and Reg4 in gut epithelial cells was determined using qRT-PCR and immunoblotting.

### Isolation of the Gut *Lactobacillus* Strain *Lactobacillus* NK6

For isolation of the gut microbiota, the cecal contents from *WT* mice were serially diluted with PBS and seeded onto *Lactobacillus*-selective culture plates. After culture under aerobic conditions or strictly anaerobic conditions at 37°C for 24–48 hr, individual colonies were picked up and cultured for an additional 1–2 days at 37°C in *Lactobacillus*-selective medium (*Lactobacillus* MRS medium) (per liter: protease peptone, 10.0 g; beef extract, 10.0 g; yeast extract, 5.0 g; Tween 80, 1.0 ml; ammonium citrate, 2.0 g; sodium acetate, 5.0 g; magnesium sulfate, 0.1 g; manganese sulfate, 0.05 g; di-potassium phosphate, 2.0 g; and glucose at 2% [w/v]). The isolated colonies were collected into stock medium (10% glycerol) and stored at −80°C. The sequences of the 16S rRNAs of the isolated colonies were obtained by cycle sequencing and were then aligned with the 16S rRNA database of GenBank using BLAST. Each inquiry gave the 100 most similar sequence results, including different bacterial genera. For each genus, one bacterial strain with the highest Max Score was selected, and its sequence was downloaded. Next, all obtained sequences were aligned by MUSLE, and then the neighbor-joining method was used to construct a phylogenetic tree.

### Flow Cytometry

Single-cell suspensions of MLNs, PPs, and spleen were prepared by mashing them in a cell strainer (70 mm), stained, and analyzed by flow cytometry. For the staining of lamina propria lymphocytes, gut epithelial cells were first removed using 1 mM EDTA and then digested in RPMI medium with 5% fetal bovine serum (FBS) and 0.15% collagenase II (275 U/mg)/0.05% dispase (1.1 U/mg) (Invitrogen) for 1 hr at 37°C. LP cells were filtered to minimize mucus contamination, stained, and analyzed by flow cytometry. Dead cells were eliminated through PI staining.

For intracellular staining, cells were cultured and stimulated for 16 hr with 50 ng/ml phorbol 12-myristate 13-acetate (PMA, Sigma) and 1 μg/ml ionomycin (Sigma) in the presence of GolgiStop (10 ng/ml, BD Biosciences). After incubation for 16 hr, cells were washed in PBS, and surface CD4 was stained with a fluorescein isothiocyanate (FITC)- or phycoerythrin (PE)-labeled anti-CD4 antibody. Cells were then washed, fixed in Cytofix/Cytoperm, permeabilized with perm/wash buffer (BD Biosciences), and stained with PE- or FITC-labeled anti-IFNγ, anti-Th17, or anti-Foxp3. Meanwhile, dead cells were eliminated through PI staining.

### In Vitro Stimulation

For in vitro stimulation, gut epithelial crypts were stimulated for 12 hr using bacteria (crypt cells: bacteria = 1:100) and then lysed for immunoblot analyses. To isolate crypts, samples were transferred to 5 mM EDTA in PBS (pH 8), shaken by hand for 1 min, incubated at 4°C for 15 min, and passaged through 70-μm filters (BD Falcon) to collect the flowthrough. The fraction containing intact and isolated crypts was collected by centrifugation at 75 × *g* for 5 min at 4°C and washed once with PBS. In some cases, bacteria (1 × $10^7$) were injected directly into colon segments to stimulate colon epithelial cells, and then colon epithelial cells were isolated using 5 mM EDTA.

### RT-PCR and qRT-PCR

RT-PCR and qRT-PCR were performed according to methods published previously (Su et al., 2014). Total RNA was extracted from the cells, tissues, and organs using TRIzol reagent (Invitrogen). First-strand cDNA was generated from total RNA using oligo-dT primers and reverse transcriptase (Invitrogen). The PCR products were visualized on 1.0% (WT/v) agarose gels. qRT-PCR was conducted using QuantiTect SYBR Green PCR Master Mix (QIAGEN) and specific primers in an ABI Prism 7000 analyzer (Applied Biosystems). GAPDH mRNA expression was detected in each experimental sample as an endogenous control. All reactions were run in triplicate.

### Immunoprecipitation and Immunoblot Analysis

Immunoprecipitation and immunoblot analysis were performed according to methods published previously (Su et al., 2014). The cells were lysed with cell lysis buffer (Cell Signaling Technology) supplemented with a protease inhibitor “cocktail” (Calbiochem). Immunoprecipitation (IP) was performed essentially as described by Thermo Scientific. For the immunoblot, hybridizations with primary antibodies were conducted for 1 hr at room temperature in blocking buffer. The protein-antibody complexes were detected using peroxidase-conjugated secondary antibodies (Boehringer Mannheim) and enhanced chemiluminescence (Amersham).

### Statistical Analysis

Student’s t test, one-way ANOVA, Bonferroni’s multiple comparison test, Wilcoxon’s test, and Mann-Whitney *U* test were used to determine significance. A 95% confidence interval was considered significant and was defined as $p < 0.05$ (*$p < 0.05$, **$p < 0.01$, ***$p < 0.001$).

## ACCESSION NUMBERS

The accession number for the gene array data reported in this paper is GEO: GSE62487.

## SUPPLEMENTAL INFORMATION

Supplemental Information includes Supplemental Experimental Procedures, seven figures, and one table and can be found with this article online at http://dx.doi.org/10.1016/j.celrep.2015.12.070.

## AUTHOR CONTRIBUTIONS

R.Y. designed the research and wrote the paper. S.C., X.S., B.Z., H. Yan, Y.H., A.W., H. Yun, and Y.Z. conducted the in vivo experiments and immunoassays. X.S, S.C., Y.H., H. Yan, E.W., and H. Yun carried out the in vitro assays. Y.C., W.L., and H.W. provided assistance for the animal experiments. Y.L. and F.L. reviewed the manuscript.

## ACKNOWLEDGMENTS

This research was supported by NSFC Grants 31470876, 91029736, 91442111, and 81370906 and ISF-NSFC Program 31461143010; Ministry of Science and Technology grants (863 Program, 2008AA02Z129, and 2014AA022209); the National Key Scientific Program (2011CB964902 and 2013CB531406); the Program for Changjiang Scholars and the Innovative Research Team in University (IRT13023); and the State Key Laboratory of Medicinal Chemical Biology.

Received: July 1, 2015
Revised: October 2, 2015
Accepted: December 14, 2015
Published: January 14, 2016

## REFERENCES

Abreu, M.T., Vora, P., Faure, E., Thomas, L.S., Arnold, E.T., and Arditi, M. (2001). Decreased expression of Toll-like receptor-4 and MD-2 correlates with intestinal epithelial cell protection against dysregulated proinflammatory gene expression in response to bacterial lipopolysaccharide. J. Immunol. *167*, 1609–1616.

Aluwihare, A.P. (1971). An ultrastructural study of the effect of neomycin on the colon in the human subject and in the conventional and the germ-free mouse. Gut *12*, 341–349.

Asensio, V.C., Lassmann, S., Pagenstecher, A., Steffensen, S.C., Henriksen, S.J., and Campbell, I.L. (1999). C10 is a novel chemokine expressed in experimental inflammatory demyelinating disorders that promotes recruitment of macrophages to the central nervous system. Am. J. Pathol. *154*, 1181–1191.

Benson, M.J., Pino-Lagos, K., Rosemblatt, M., and Noelle, R.J. (2007). All-trans retinoic acid mediates enhanced T reg cell groWTh, differentiation, and gut homing in the face of high levels of co-stimulation. J. Exp. Med. *204*, 1765–1774.

Brandl, K., Plitas, G., Schnabl, B., DeMatteo, R.P., and Pamer, E.G. (2007). MyD88-mediated signals induce the bactericidal lectin RegIII gamma and protect mice against intestinal Listeria monocytogenes infection. J. Exp. Med. *204*, 1891–1900.

Chai, L., Dai, L., Che, Y., Xu, J., Liu, G., Zhang, Z., and Yang, R. (2009). LRRC19, a novel member of the leucine-rich repeat protein family, activates NF-kappaB and induces expression of proinflammatory cytokines. Biochem. Biophys. Res. Commun. *388*, 543–548.

Clemente, J.C., Ursell, L.K., Parfrey, L.W., and Knight, R. (2012). The impact of the gut microbiota on human health: an integrative view. Cell *148*, 1258–1270.

Coelho, A.L., Schaller, M.A., Benjamim, C.F., Orlofsky, A.Z., Hogaboam, C.M., and Kunkel, S.L. (2007). The chemokine CCL6 promotes innate immunity via immune cell activation and recruitment. J. Immunol. *179*, 5474–5482.

Coombes, J.L., Siddiqui, K.R., Arancibia-Cárcamo, C.V., Hall, J., Sun, C.M., Belkaid, Y., and Powrie, F. (2007). A functionally specialized population of mucosal CD103+ DCs induces Foxp3+ regulatory T cells via a TGF-beta and retinoic acid-dependent mechanism. J. Exp. Med. *204*, 1757–1764.

de Kivit, S., Tobin, M.C., Forsyth, C.B., Keshavarzian, A., and Landay, A.L. (2014). Regulation of Intestinal Immune Responses through TLR Activation: Implications for Pro- and Prebiotics. Front. Immunol. *5*, 60.

Dove, W.F., Clipson, L., Gould, K.A., Luongo, C., Marshall, D.J., Moser, A.R., NeWTon, M.A., and Jacoby, R.F. (1997). Intestinal neoplasia in the ApcMin mouse: independence from the microbial and natural killer (beige locus) status. Cancer Res. *57*, 812–814.

Goto, Y., and Ivanov, I.I. (2013). Intestinal epithelial cells as mediators of the commensal-host immune crosstalk. Immunol. Cell Biol. *91*, 204–214.

Harris, T.H., Banigan, E.J., Christian, D.A., Konradt, C., Tait Wojno, E.D., Norose, K., Wilson, E.H., John, B., Weninger, W., Luster, A.D., et al. (2012). Generalized Lévy walks and the role of chemokines in migration of effector CD8+ T cells. Nature *486*, 545–548.

Ivanov, I.I., and Honda, K. (2012). Intestinal commensal microbes as immune modulators. Cell Host Microbe *12*, 496–508.

Jiang, W., Wang, X., Zeng, B., Liu, L., Tardivel, A., Wei, H., Han, J., MacDonald, H.R., Tschopp, J., Tian, Z., and Zhou, R. (2013). Recognition of gut microbiota by NOD2 is essential for the homeostasis of intestinal intraepithelial lymphocytes. J. Exp. Med. *210*, 2465–2476.

Ley, R.E., Turnbaugh, P.J., Klein, S., and Gordon, J.I. (2006). Microbial ecology: human gut microbes associated with obesity. Nature *444*, 1022–1023.

Li, Y., Kundu, P., Seow, S.W., de Matos, C.T., Aronsson, L., Chin, K.C., Kärre, K., Pettersson, S., and Greicius, G. (2012). Gut microbiota accelerate tumor groWTh via c-jun and STAT3 phosphorylation in APCMin/+ mice. Carcinogenesis *33*, 1231–1238.

Luddy, K.A., Robertson-Tessi, M., Tafreshi, N.K., Soliman, H., and Morse, D.L. (2014). The role of toll-like receptors in colorectal cancer progression: evidence for epithelial to leucocytic transition. Front. Immunol. *5*, 429.

Melmed, G., Thomas, L.S., Lee, N., Tesfay, S.Y., Lukasek, K., Michelsen, K.S., Zhou, Y., Hu, B., Arditi, M., and Abreu, M.T. (2003). Human intestinal epithelial cells are broadly unresponsive to Toll-like receptor 2-dependent bacterial ligands: implications for host-microbial interactions in the gut. J. Immunol. *170*, 1406–1415.

Mowat, A.M., and Viney, J.L. (1997). The anatomical basis of intestinal immunity. Immunol. Rev. *156*, 145–166.

Mukherjee, S., Zheng, H., Derebe, M.G., Callenberg, K.M., Partch, C.L., Rollins, D., Propheter, D.C., Rizo, J., Grabe, M., Jiang, Q.X., and Hooper, L.V. (2014). Antibacterial membrane attack by a pore-forming intestinal C-type lectin. Nature *505*, 103–107.

Nguyen, L.P., Pan, J., Dinh, T.T., Hadeiba, H., O'Hara, E., 3rd, Ebtikar, A., Hertweck, A., Gökmen, M.R., Lord, G.M., Jenner, R.G., et al. (2015). Role and species-specific expression of colon T cell homing receptor GPR15 in colitis. Nat. Immunol. *16*, 207–213.

Niess, J.H., and Adler, G. (2010). Enteric flora expands gut lamina propria CX3CR1+ dendritic cells supporting inflammatory immune responses under normal and inflammatory conditions. J. Immunol. *184*, 2026–2037.

Núñez, V., Alameda, D., Rico, D., Mota, R., Gonzalo, P., Cedenilla, M., Fischer, T., Boscá, L., Glass, C.K., Arroyo, A.G., and Ricote, M. (2010). Retinoid X receptor alpha controls innate inflammatory responses through the up-regulation of chemokine expression. Proc. Natl. Acad. Sci. USA *107*, 10626–10631.

Popivanova, B.K., Kostadinova, F.I., Furuichi, K., Shamekh, M.M., Kondo, T., Wada, T., Egashira, K., and Mukaida, N. (2009). Blockade of a chemokine, CCL2, reduces chronic colitis-associated carcinogenesis in mice. Cancer Res. *69*, 7884–7892.

Rakoff-Nahoum, S., Hao, L., and Medzhitov, R. (2006). Role of toll-like receptors in spontaneous commensal-dependent colitis. Immunity *25*, 319–329.

Round, J.L., and Mazmanian, S.K. (2009). The gut microbiota shapes intestinal immune responses during health and disease. Nat. Rev. Immunol. *9*, 313–323.

Sawa, S., Cherrier, M., Lochner, M., Satoh-Takayama, N., Fehling, H.J., Langa, F., Di Santo, J.P., and Eberl, G. (2010). Lineage relationship analysis of RORgammat+ innate lymphoid cells. Science *330*, 665–669.

Sorbara, M.T., and Philpott, D.J. (2011). Peptidoglycan: a critical activator of the mammalian immune system during infection and homeostasis. Immunol. Rev. *243*, 40–60.

Su, X., Min, S., Cao, S., Yan, H., Zhao, Y., Li, H., Chai, L., Mei, S., Yang, J., Zhang, Y., et al. (2014). LRRC19 expressed in the kidney induces TRAF2/6-mediated signals to prevent infection by uropathogenic bacteria. Nat. Commun. *5*, 4434.

Turnbaugh, P.J., Hamady, M., Yatsunenko, T., Cantarel, B.L., Duncan, A., Ley, R.E., Sogin, M.L., Jones, W.J., Roe, B.A., Affourtit, J.P., et al. (2009). A core gut microbiome in obese and lean twins. Nature *457*, 480–484.

Vaishnava, S., Yamamoto, M., Severson, K.M., Ruhn, K.A., Yu, X., Koren, O., Ley, R., Wakeland, E.K., and Hooper, L.V. (2011). The antibacterial lectin RegIIIgamma promotes the spatial segregation of microbiota and host in the intestine. Science *334*, 255–258.

Vijay-Kumar, M., Aitken, J.D., Carvalho, F.A., Cullender, T.C., Mwangi, S., Srinivasan, S., Sitaraman, S.V., Knight, R., Ley, R.E., and Gewirtz, A.T. (2010). Metabolic syndrome and altered gut microbiota in mice lacking Toll-like receptor 5. Science *328*, 228–231.

Zhan, Y., Chen, P.J., Sadler, W.D., Wang, F., Poe, S., Núñez, G., Eaton, K.A., and Chen, G.Y. (2013). Gut microbiota protects against gastrointestinal tumorigenesis caused by epithelial injury. Cancer Res. *73*, 7199–7210.

Zhao, X., Sato, A., Dela Cruz, C.S., Linehan, M., Luegering, A., Kucharzik, T., Shirakawa, A.K., Marquez, G., Farber, J.M., Williams, I., and Iwasaki, A. (2003). CCL9 is secreted by the follicle-associated epithelium and recruits dome region Peyer's patch CD11b+ dendritic cells. J. Immunol. *171*, 2797–2803.

Cell Reports
Supplemental Information

# The Gut Epithelial Receptor LRRC19 Promotes the Recruitment of Immune Cells and Gut Inflammation

Shuisong Cao, Xiaomin Su, Benhua Zeng, Hui Yan, Yugang Huang, Enlin Wang, Huan Yun, Yuan Zhang, Feifei Liu, Wenxia Li, Hong Wei, Yongzhe Che, and Rongcun Yang

1) **Supplemental data items**

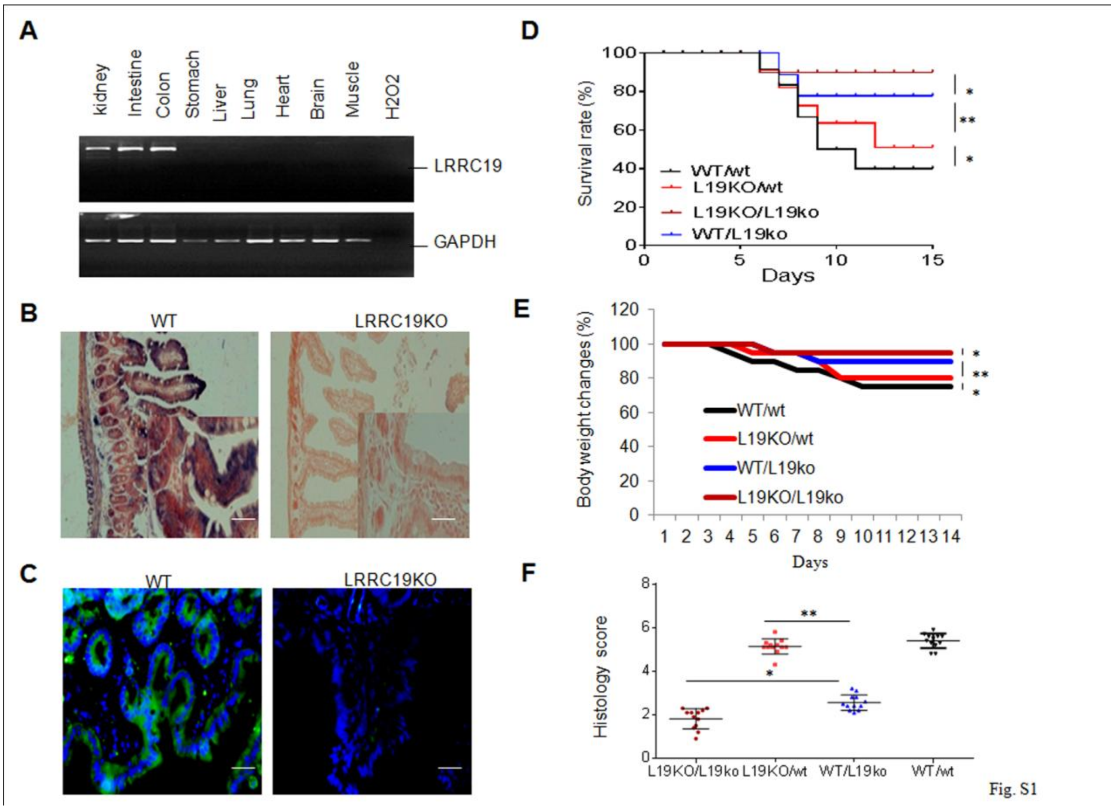

Fig. S1

**Figure S1. Related to Figure 1 and 2; DSS-mediated colitis in chimeric mice.**
(A, B and C) RT-PCR, *in situ* hybridization and immune-staining of LRRC19 in gut tissues. Mouse kidney, intestine, colon, stomach, liver, lung, heart, brain and muscle tissues were analyzed using RT-PCR for LRRC19 expression (A). Mouse colon tissues from *wt* (WT) and *Lrrc19* KO (Lrrc19KO) mice were detected by incubation with the anti-sense probe of LRRC19 (B) or anti-LRRC19 antibody (C); blue in B and green in C indicated LRRC19. (D) Survival rates of different chimeric mice (n=16) after feeding 2% DSS-solution. Different chimeric mice were fed by a 2% DSS-solution in drinking water for 7 days, then switched to regular drinking water. (E) Changes of body weight among the different chimeric mice after feeding 2% DSS-solution in drinking water for 7 days. (F) Histology score of different chimeric mice after feeding 2% DSS-solution. Histological scores were assessed according to the methods described in supplementary EXPERIMENTAL PROCEDURES. WT/wt indicates that bone marrow (BM) cells of WT mice were transplanted into lethally irradiated WT mice; WT/L19ko indicates BM cells of WT mice into lethally irradiated *Lrrc19* KO mice; L19KO/wt indicates BM cells of *Lrrc19* KO mice into lethally irradiated WT mice; L19KO/L19ko indicates BM cells of *Lrrc19* KO mice into lethally irradiated *Lrrc19* KO mice. The data are representative of three independent experiments. *$P$ < 0.05 and **$P$ < 0.01 (Wilcoxon's test in A; ANOVA test in B; Mann-Whitney U test in C). Scale bars, 40 µm.

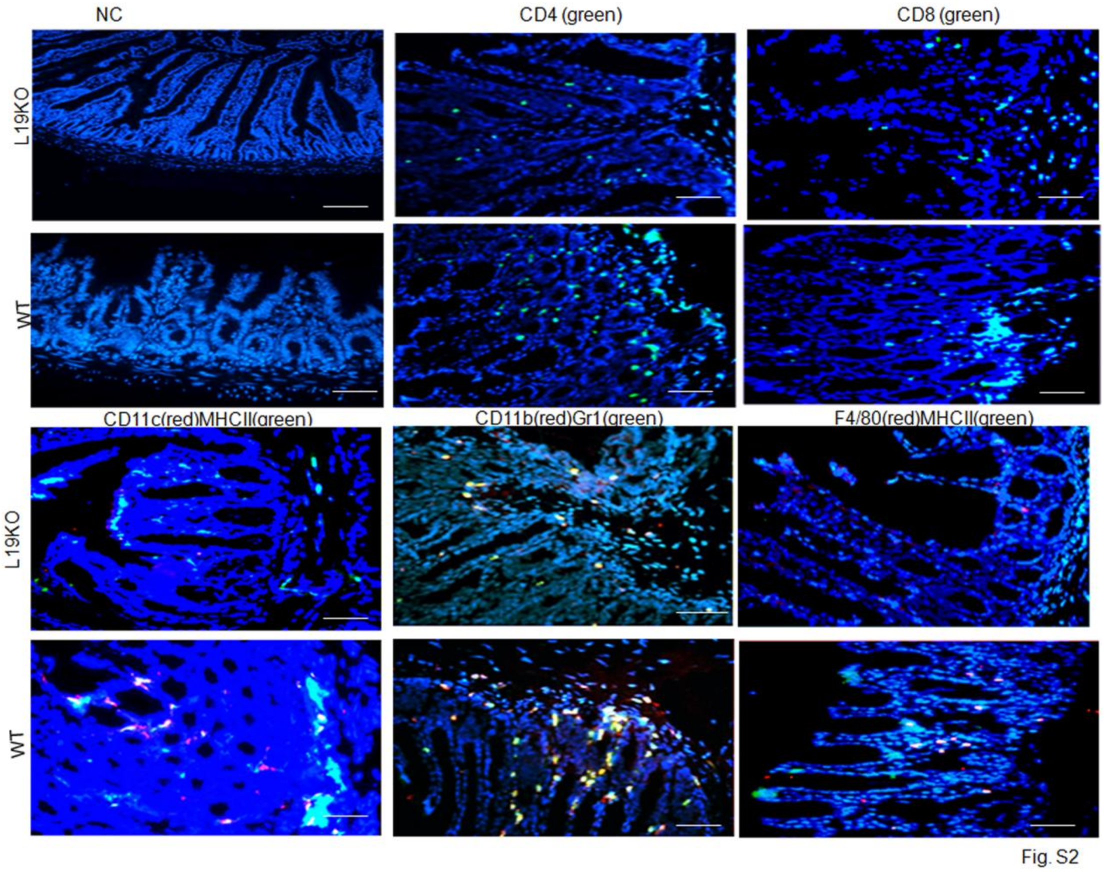


**Figure S2. Related to Figure 3; Immuno-staining of CD4$^+$T cells, CD8$^+$ T cells, CD11c$^+$MHCII$^+$, CD11b$^+$Gr1$^+$ and F4/80$^+$MHCII$^+$ cells in *wt* (WT) and *Lrrc19* KO (L19KO) mice.** The colon tissues were frozen, sliced, and stained by FITC-labeled anti-CD4 or anti-CD8, FITC-labeled anti- MHCII or anti-Gr-1, PE-labeled anti-CD11c, anti-CD11b or anti-F4/80 respectively. NC, isotypic control; Blue, DAPI staining. Scale bars, 40μm.

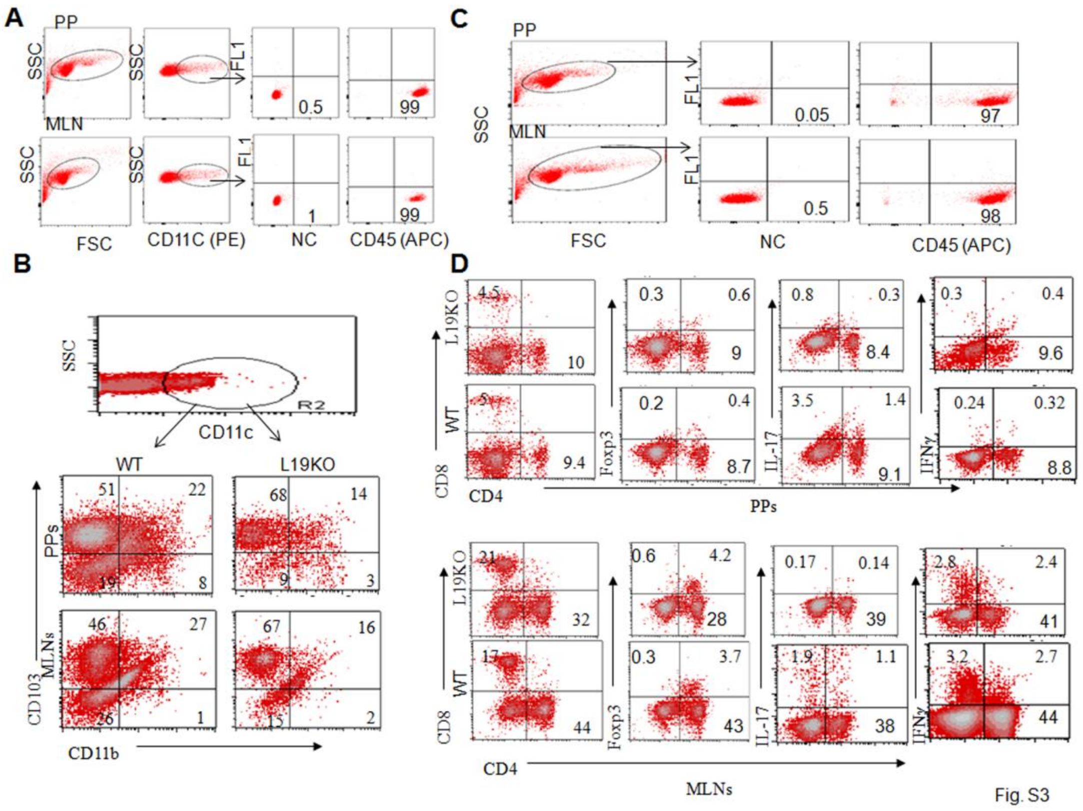


**Figure S3. Related to Figure 3;** (A) The percentages of CD45+ cells in the gated CD11c$^{+}$cells of PPs and MLNs by flow cytometry analyses; Numbers, percentages in the gated CD11c$^{+}$cells. NC, isotypic control. (B) The percentages of CD11c$^{+}$CD103$^{+}$CD11b$^{-}$ DCs (CD103$^{+}$DC), CD11C$^{+}$CD103$^{+}$CD11b$^{+}$DCs (DP-DC), CD11C$^{+}$CD11b$^{-}$ CD103$^{-}$ DCs (DNDC) and CD11C$^{+}$CD11b$^{+}$ (CD11b$^{+}$DC) in PPs and MLNs by flow cytometry analyses; Numbers, percentages in the gated CD11c$^{+}$cells. (C) The percentages of CD45+ cells in PPs and MLNs by flow cytometry analyses. NC, isotypic control. (D) The percentages of CD4$^{+}$, CD8$^{+}$, CD4$^{+}$Foxp3$^{+}$, CD4$^{+}$IL-17$^{+}$ and CD4$^{+}$IFNγ$^{+}$ cells in PPs and MLNs of *wt* (WT) and *Lrrc19* KO (L19KO) mice. PPs and MLNs were mashed, stained using the indicated antibodies, and subjected to flow cytometry. Cell percentages in age and gender matched *wt* (WT, n=6) and *Lrrc19* KO (L19KO, n=6) mice were compared.

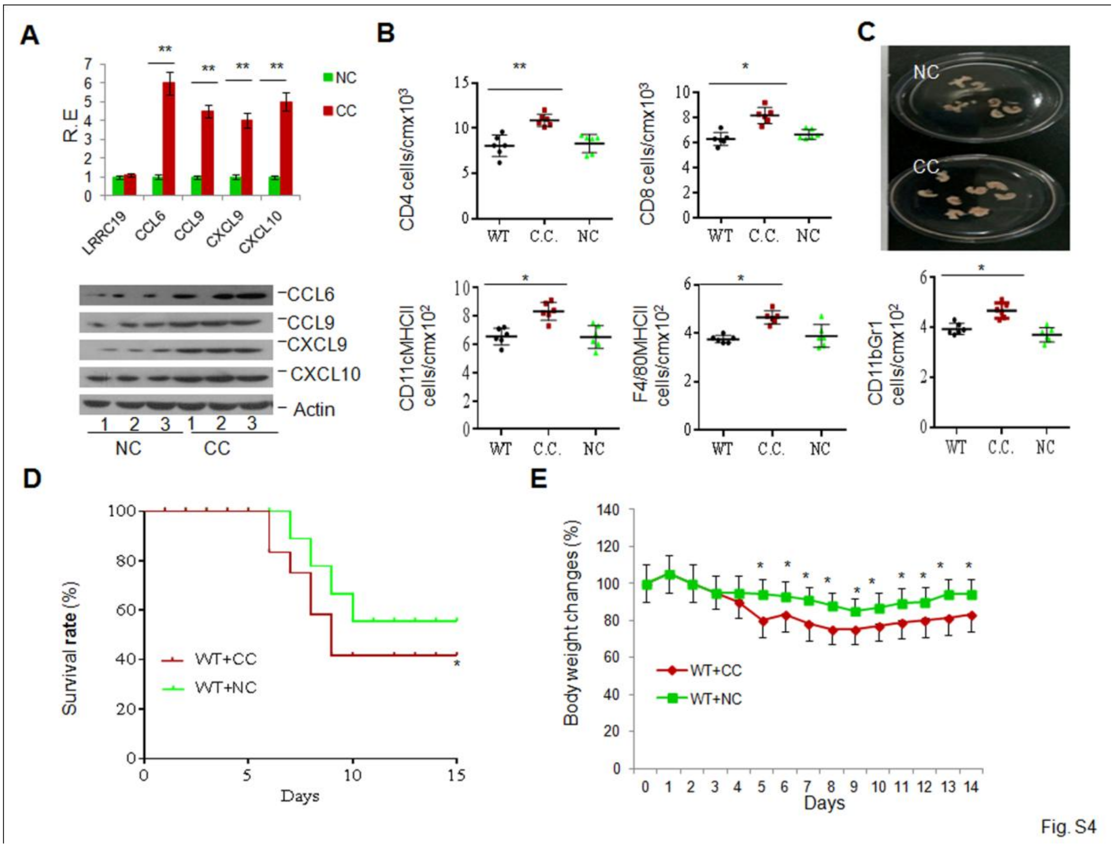


**Figure S4. Related to Figure 4; Chemokines CCL6, CCL9, CXCL9 and CXCL10 promoted sensitivity to DSS-mediated colitis.** (A) qRT-PCR and immunoblot of chemokines CCL6, CCL9, CXCL9 and CXCL10 in mice with (CC, chemokine complexes) or without (NC, negative control) CCL6, CCL9, CXCL9 and CXCL10 adenovirus injection. Actin, a loading control. (B) Absolute number of CD4$^+$ and CD8$^+$ T cells, CD11C$^+$MHCII$^+$ DCs, F4/80MHCII and CD11b$^+$Gr1$^+$ cells in the colon tissue of mice with (CC) or without (NC) chemokine adenovirus injection by flow cytometric analyses. CC: CCL6, CCL9, CXCL9 and CXCL10 complexes. WT: cell numbers from *wt* mice. (C) Size of MLNs from mice with (CC) or without (NC) chemokines expressing adenovirus injection. (D and E) Survival (D) and body weight (E) were monitored until day 14 after the start of DSS. Mice with (CC, n=18) or without (NC, n=18） CCL6, CCL9, CXCL9 and CXCL10 adenovirus injection were fed by a 2% DSS-solution in drinking water for 7 days, then switched to regular drinking water. *P<0.05, **P<0.01 (*t*-test in A, mean ± SD; Mann-Whitney U test in B; Wilcoxon's test in D; ANOVA test in E).

>A10253_18#_1407035775Y.seq.Contig1TGGATGGACCTGCGGTGCATTAG
CTAGTTGGTAAGGTAACGGCTTACCAAGGCAATGATGCATAGCCGAGTTG
AGAGACTGATCGGCCACATTGGGACTGAGACACGGCCCAAACTCCTACG
GGAGGCAGCAGTAGGGAATCTTCCACAATGGACGCAAGTCTGATGGAGC
AACGCCGCGTGAGTGAAGAAGGGTTTCGGCTCGTAAAGCTCTGTTGGTAG
TGAAGAAAGATAGAGGTAGTAACTGGCCTTTATTTGACGGTAATTACCTAG
AAAGTCACGGCTAACTACGTGCCAGCAGCCGCGGTAATACGTAGGTGGC
AAGCGTTGTCCGGATTTATTGGGCGTAAAGCGAGTGCAGGCGGTTCAATA
AGTCTGATGTGAAAGCCTTCGGCTCAACCGGAGAATTGCATCAGAAACTG
TTGAACTTGAGTGCAGAAGAGGAGAGTGGAACTCCATGTGTAGCGGTGGA
ATGCGTAGATATATGGAAGAACACCAGTGGCGAAGGCGGCTCTCTGGTCT
GCAACTGACGCTGAGGCTCGAAAGCATGGGTAGCGAACAGGATTAGATA
CCCTGGTAGTCCATGCCGTAAACGATGAGTGCTAAGTGTTGGGAGGTTTC
CGCCTCTCAGTGCTGCAGCTAACGCATTAAGCACTCCGCCTGGGGAGTAC
GACCGCAAGGTTGAAACTCAAAGGAATTGACGGGGGCCCGCACAAGCGG
TGGAGCATGTGGTTTAATTCGAAGCAACGCGAAGAACCTTACCAGGTCTT
GACATCCAGTGCAAACCTAAGAGATTAGGTGTTCCCTTCGGGGACGCTGA
GACAGGTGGTGCATGGCTGTCGTCAGCTCGTGTCGTGAGATGTTGGGTTA
AGTCCCGCAACGAGCGCAACCCTTGTCATTAGTTGCCATCATTAAGTTGG
GCACTCTAATGAGACTGCCGGTGACAAACCGGAGGAAGGTGGGGATGAC
GTCAAGTCATCATGCCCCTTATGACCTGGGCTACACACGTGCTACAATGG
ACGGTACAACGAGAAGCGAACCTGCGAAGGCAAGCGGATCTCTTAAAGC
CGTTCTCAGTTCGGACTGTAGGCTGCAACTCGCCTACACGAAGCTGGAAT
CGCTAGTAATCGCGGATCAGCACGCCGCGGTGAATACGTTCCCGGGCCT
TGTACACACCGCCCGTCACACCATGAGAGTCTGTAACACCCAAAGCCGGT
GGGATAACCTTTATAGGAGTCAG

Fig. S5

**Figure S5. Related to Figure S6;** Sequence of *Lacbacillus* NK6 16S rRNA by primers (Forward, 5’-AGAGTTTGATCMTGGCTCAG; Reverse, 5’-ACGGCTACCTTGTTACGACTT). This sequence has also been shown in another paper.

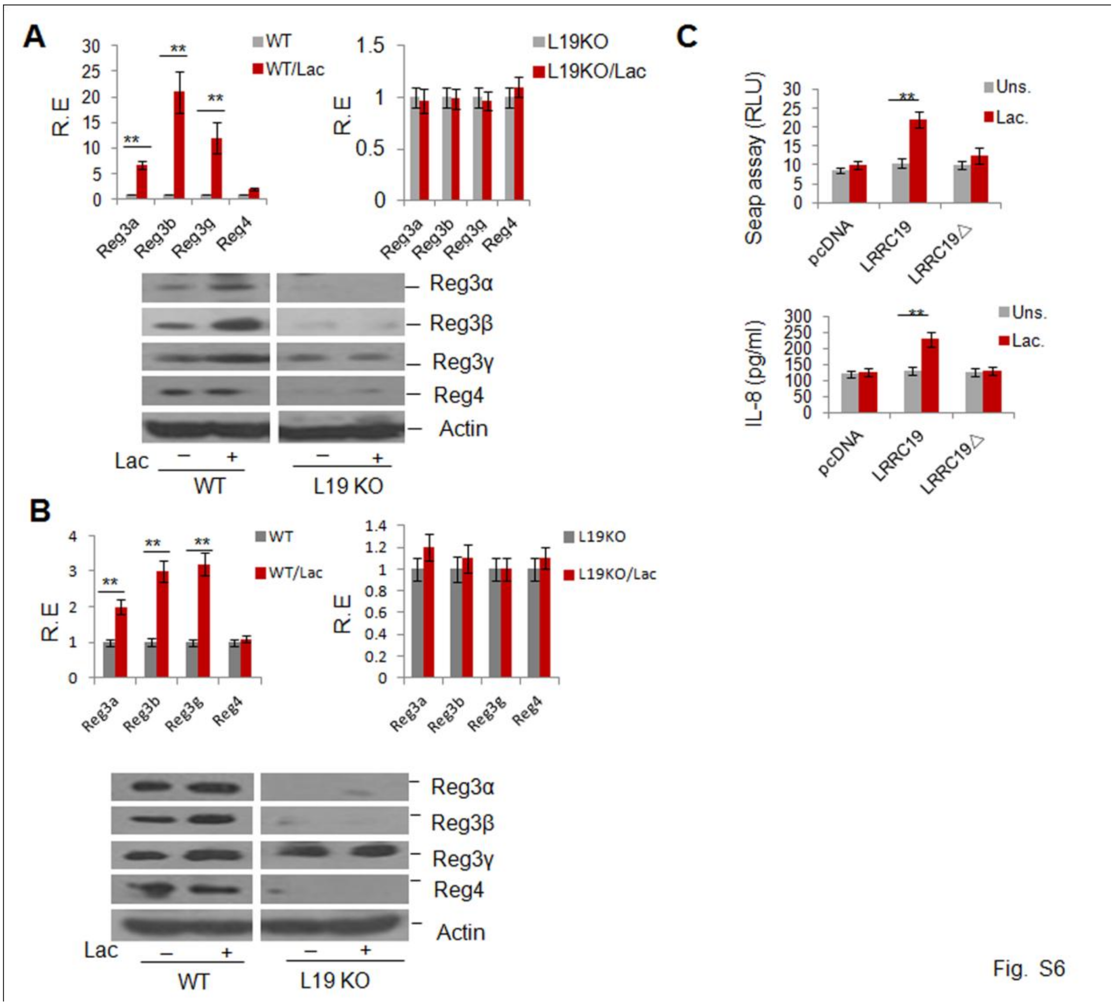


**Figure S6. Related to Figure 6; *Lactobacillus* modulates the expression of REGs through LRRC19.** (A) qRT-PCR and immunoblot of REG3α, REG3β, REG3γ and REG4 in the gut tissues of *wt* and *L19* KO mice in response to *Lactobacillus* NK6. A 4-cm gut segment was stimulated by infusion with *lactobacillus* NK6 and analyzed after 16 hrs. (B) qRT-PCR and immunoblot of REG3α, REG3β, REG3γ and REG4 in the gut epithelial cells of pan-antibiotics-treated *wt* and *L19KO* mice after transplanting *Lactobacillus* NK6 for 3 days. Antibiotics-treated mice were intragastrically given *Lactobacillus* ($1 \times 10^8$) (n=3) and the expression of REG3α, REG3β, REG3γ and REG4 in gut epithelial cells was analyzed after 3 days. For antibiotics-treated mice, 6- to 8-week-old mice were treated with ampicillin (A, 1 g/L, Sigma), vancomycine (V, 0.5g/L), neomycin sulfate (N, 1 g/L), and metronidazole (M, 1g/L) in drinking water for 4 weeks via the drinking water. Water containing antibiotic was exchanged every three days. To confirm the elimination of bacteria, stool was collected from antibiotic-treated and -untreated mice and cultured in anaerobic and aerobic condition. The bacteria were counted under microscope. (C) NF-κB (upper) activity and endogenous cytokine production (lower) in LRRC19-transfected 293T cells after exposed to *Lactobacillus*. 293T cells were co-transfected with pNF-κB-SEAP (secreted alkaline phosphatase) and the expression plasmids of LRRC19 or LRRC19Δ (absence of extracellular region) by Lipofectamine 2000 (Invitrogen). Alkaline phosphatase activity in the supernatants was measured following treatment with *Lactobacillus*. Uns, untreated cells. IL-8 secretion was detected by ELISA kit according to the manufacturer's protocol (Jingmei Corp.). The empty expression vector (pcDNA3.1) was used as control. RLU, relative light

units. *P<0.05, **P<0.01 (*t*-test; mean ± SD). The data are a representative of three independent experiments.

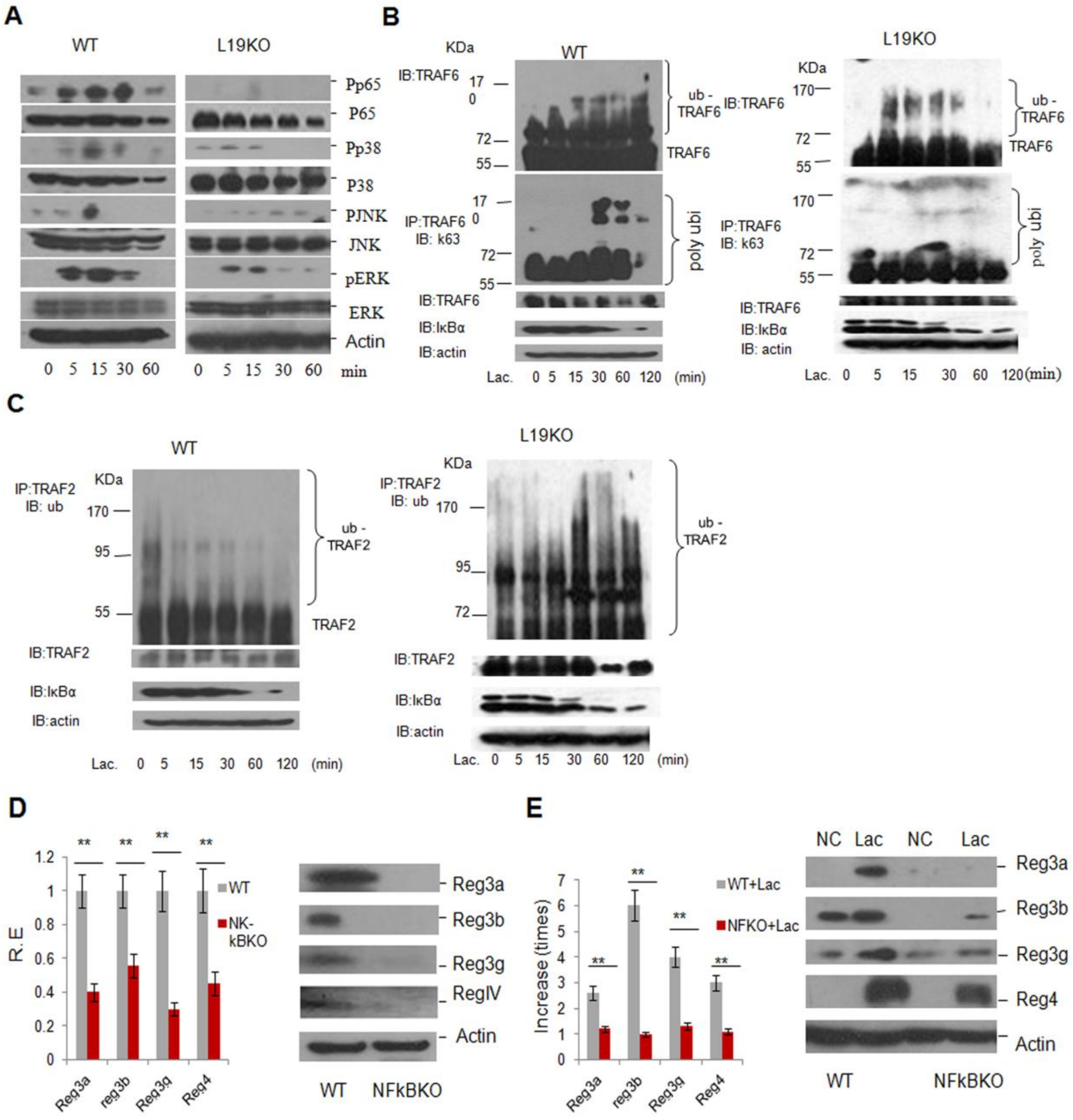


Fig. S7

**Figure S7. Related to Figure 6; LRRC19 induces expression of REGs through TRAF2 and TRAF6 ubiquitination mediated signal pathway.** (A) Phosphorylation analyses of p38, JNK, and ERK. The cells were lysed at the indicated time. A 4-cm gut segment was stimulated by infusion with *lactobacillus* NK6 and analyzed at the indicted time points. The gut mucosa was scraped and lysed, and phosphor-p38, -ERK and -JNK were detected using anti-phosphor-p38, -ERK or-JNK antibody. Actin, a loading control. (B) Immunoblot of K63-linked ubiquitination (K63-Ub) of endogenouse TRAF6

immunoprecipitated from *wt* and *Lrrc19* KO (L19KO)gut epithelial cells stimulated with *lactobacillus* NK6 (middle blot), and immunoblot analysis of TRAF6 (top blot), IkBα and actin (below blots) in the same cells without immunoprecipitation. IP, immunoprecipitation; IB, immunoblot assay. (C) Immunoblot analysis of ubiquitination of endogenouse TRAF2 immunoprecipitated from *wt* and *Lrrc19* KO gut epithelial cells stimulated with *lactobacillus* NK6 (upper blots), and immunoblot analysis of TRAF2, IκBα and actin (below blots) in the same cells without immunoprecipitation. (D) qRT-PCR (right) and immunoblot (left) of REG3α, REG3β, REG3γ and REG4 in *wt* and *Nf-κb* KO (L19KO) gut tissues. (E) qRT-PCR (right) and immunoblot (left) of REG3α, REG3β, REG3γ and REG4 in gut epithelial cells of *wt* and *Nf-κb* KO mice after exposed to *Lactobacillus* NK6. *P<0.05, **P<0.01 (*t*-test in D and E; mean ± SD)

**Table S1. Related to Figure 6; The profiling of bacteria genus in ileum (proximal), ileum (distal), caecum and colon.**

| Genus | Ileu（proximal） | | Ileum（distal） | | Caecum | | Colon | |
|---|---|---|---|---|---|---|---|---|
| | WT | KO | WT | KO | WT | KO | WT | KO |
| Enterorhabdus | 12 | 12 | 5 | 37 | 3 | 1 | 1 | 2 |
| Coriobacteriaceae;Other | 61 | 36 | 47 | 132 | 16 | 5 | 4 | 7 |
| Actinobacteria;Other;Other;Other | 6 | 12 | 0 | 11 | 5 | 0 | 0 | 0 |
| Porphyromonadaceae; Other | 19 | 21 | 20 | 25 | 13 | 4 | 8 | 23 |
| Parabacteroides | 0 | 1 | 4 | 15 | 17 | 28 | 15 | 26 |
| Prevotellaceae;Other | 30 | 49 | 28 | 723 | 799 | 897 | 960 | 1424 |
| Paraprevotella | 7 | 22 | 0 | 20 | 214 | 272 | 13 | 25 |
| Alistipes | 4 | 0 | 5 | 5 | 36 | 14 | 18 | 28 |
| Rikenella | 0 | 0 | 75 | 20 | 0 | 8 | 9 | 35 |
| Bacteroides | 2 | 11 | 6 | 95 | 154 | 273 | 74 | 160 |
| Bacteroidales;Other;Other | 19 | 0 | 75 | 341 | 17 | 12 | 110 | 245 |
| Bacteroidetes;Other;Other;Other;Other | 830 | 1153 | 2634 | 1982 | 607 | 731 | 534 | 781 |
| Achromobacter | 26 | 37 | 56 | 216 | 2 | 2 | 0 | 0 |
| Parasutterella | 207 | 258 | 118 | 87 | 36 | 48 | 18 | 44 |
| Helicobacter | 281 | 329 | 11 | 191 | 53 | 62 | 25 | 163 |
| Serratia | 13 | 7 | 3 | 22 | 0 | 0 | 0 | 1 |
| Stenotrophomonas | 2 | 0 | 0 | 6 | 0 | 0 | 0 | 0 |
| Proteobacteria;Other;Other;Other;Other | 6 | 0 | 0 | 0 | 4 | 2 | 8 | 11 |
| Mycoplasma | 18 | 107 | 1 | 4 | 1 | 9 | 1 | 14 |
| Ureaplasma | 21 | 27 | 99 | 2039 | 1 | 3 | 1 | 13 |
| Jeotgalicoccus | 4 | 0 | 2 | 0 | 0 | 0 | 0 | 0 |
| Aerococcus | 1 | 0 | 2 | 0 | 0 | 0 | 0 | 0 |
| Enterococcus | 1 | 0 | 2 | 0 | 0 | 0 | 0 | 0 |
| Lactobacillus | 546 | 120 | 833 | 281 | 57 | 20 | 13 | 31 |
| Streptococcaceae;Other | 1 | 0 | - | - | 0 | 0 | - | - |
| Streptococcus | 3 | 0 | 9 | 21 | 0 | 0 | 0 | 0 |

| | | | | | | | | |
|---|---|---|---|---|---|---|---|---|
| Clostridium_XlVb | 2 | 2 | 1 | 14 | 30 | 13 | 32 | 28 |
| Dorea | - | - | 4 | 1 | 2 | 0 | 2 | 0 |
| Marvinbryantia | - | - | 2 | 1 | 4 | 7 | 3 | 5 |
| Lachnospiraceae;Other | 111 | 15 6 | 307 | 230 | 1717 | 1260 | 1089 | 1257 |
| Clostridiales;Other;Other | 44 | 53 | 129 | 95 | 1368 | 548 | 912 | 306 |
| Clostridium_XI | 32 | 57 | 2 | 0 | 10 | 0 | 3 | 1 |
| Anaerotruncus | 1 | 5 | 1 | 3 | 30 | 24 | 8 | 9 |
| Butyricicoccus | 0 | 2 | 3 | 4 | 55 | 12 | 10 | 18 |
| Oscillibacter | 2 | 3 | 0 | 8 | 79 | 44 | 42 | 38 |
| Ruminococcaceae;Other | 12 | 11 | 34 | 36 | 188 | 147 | 103 | 133 |
| Clostridia;Other;Other;Other | 2 | 29 | 2 | 3 | 229 | 40 | 15 | 8 |
| Clostridium_XVIII | 2 | 6 | 6 | 8 | 6 | 5 | 3 | 3 |
| Firmicutes;Other;Other;Other;Other | 39 | 18 | 152 | 22 | 372 | 107 | 28 | 26 |
| Bacteria;Other;Other;Other;Other;Other | 1767 | 26 04 | 528 | 840 | 806 | 936 | 15 | 26 |

Notes: Wt, wild type mice; KO, *Lrrc19* KO mice.

## 2) Supplemental Experimental Procedures

### Reagents

Anti-mouse p38 (D13E1, Cell Signaling), JNK (sc-137020, Santa), ERK (197G2, Cell Signaling), STAT3 (124H6, Cell Signaling), phosphorylated-STAT3 (EP2147Y, Abcam), phosphorylated-p38 (pp38) (D3F9, Cell Signaling), phosphorylated JNK (sc-81502, Santa), phosphorylated ERK (D13.14.4E, Cell Signaling), phosphorylated -IκBα (14D4, Cell Signaling), IκBα (L35A5, Cell Signaling), p-65 (sc8008, Santa), phosphorylated-p-65 (sc-52401, Santa), LRRC19 (PA5-20914, Thermo), and β-actin (sc-47778, Santa) were purchased; FITC-, PE- or APC-conjugated anti-mouse CD4 (RM4-5, Biolegend), CD8 (ZUT270.5, Biolegend), MHCII (I-A/I-E, M5/114.115.2, Biolegend), CD11c (MCA1441GA, Biolegend), CD103 (2E7, Biolegend), CD11b (M1/70, eBioscience), F4/80 (BM8, Biolegend), Gr-1 (RB6-8C5, eBioscience), IFNγ (XMG1.2, Biolegend), Foxp3 (MF23, eBioscience) and IL17A (eBio17B7, eBioscience) antibodies were purchased. Anti- ubiquitin (YT4793, Immunoway), K63-Ub (HWA4C4, Enzo), TRAF2 (EPR6048, Epitomics), TRAF6 (sc-8408, Santa) antibodies; anti-mouse CCL6 (EPR14614, Abcam), CCL9 (ab9913, Abcam), CXCL9 (orb13424, Biorbyt), CXCL10 (ab9938, Abcam), Reg3α (M-44, Santa), Reg3β (AF5110, Sigma), Reg3γ (PA517, Thermo) and Reg4 (H-40, Santa) were also purchased. HA- or flag- tagged LRRC19, CCL6, CCL9, CXCL9, CXCL10 Reg3α, Reg3β, Reg3γ and Reg4 adenoviruses were prepared by ABM, Canada and expanded by GeneChem, Shanghai.

### DSS induced colitis

Dextran sodium sulfate (DSS) induced colitis was performed according to the reported method (Chen et al., 2013) with modification. Briefly, mice received 2% (wt/vol) DSS (40,000 kDa; ICN Biochemicals) or indicated doses in their drinking water for 7 days, then switched to regular drinking water. The amount of DSS water drank per animal was recorded and no differences in intake between strains were observed. For survival studies, mice were followed for 14 days post start of DSS-treatment. Mice were weighed every other day for the determination of percent weight change. This was calculated as: % weight change = (weight at day X-day 0 / weight at day 0) × 100. Animals were monitored clinically for rectal bleeding, diarrhea, and general signs of morbidity, including hunched posture and failure to groom. Mice were then sacrificed at the indicated days for histological study. Representative colon tissues were embedded in paraffin for hematoxylin/eosin (H&E) staining or embedded in OCT compound (Tissue-Tek, Sakura, Torrance, CA) and frozen over liquid nitrogen for immuno-staining. For histological evaluation, colonic epithelial damage was scored blindly as follows: 0 = normal; 1 = hyper-proliferation, irregular crypts, and goblet cell loss; 2 = mild to moderate crypt loss (10–50%); 3 = severe crypt loss (50–90%); 4 = complete crypt loss, surface epithelium intact; 5 = small to medium sized ulcer (<10 crypt widths); 6 = large ulcer (>10 crypt widths) (Lee et al., 2006).

### Tumorigenesis Procedure

Tumorigenesis procedure was performed according to the reported method (Greten et al., 2004). Briefly, 7-8-week-old mice were injected with AOM (Sigma) dissolved in 0.9% NaCl intraperitoneally at a dose of 12.5 mg/kg body weight. 5 days after injection, mice were treated with 2 % DSS in drinking water, then followed by regular water for 16 days. This cycle was repeated twice (at the third cycle, mice were treated with 2.0% DSS for 4 days). 2 weeks after DSS treatment, mice were sacrificed and murine colon was removed and flushed carefully with PBS buffer. Colon was then cut longitudinally

and fixed flat in 10% neutral buffered formalin overnight. All of the colon tumors were counted and measured under a stereomicroscope. Representative colon cancer tissues were embedded in paraffin for hematoxylin/eosin (H&E) staining or embedded in OCT compound and frozen over liquid nitrogen for immuno-staining. Histology analysis was carried out on H&E or immuno-staining tumor sections.

**Histological and immune staining**

For hematoxylin/eosin (H&E) staining, previously reported methods were used in this experiment (Su et al., 2014)**.** Briefly, the entire colon was excised to measure the length of the colon and then were fixed in 4% (w/v) paraformaldehyde buffered saline and embedded in paraffin, 5 μm sections colon sections were cut and stained with H&E.

For immune staining, colon tissues were embedded in OCT compound (Tissue-Tek, Torrance, CA) and frozen over liquid nitrogen. 5-μm-thick sections were prepared from frozen tissue and fixed in acetone (−20°C) for 10 min. After rehydration in PBS for 5 min and further washing in PBS, tissue sections were blocked with 1% (w/v) BSA and 0.2% (w/v) milk powder in PBS (PBS-BB). The primary antibody was added in PBS-BB and incubated overnight at 4℃. After PBS washing (three times, 5 min each), tissue was detected with DAB kit or fluorescence labeled second antibody. Nuclei were stained by DAPI.

For BrdU assay, 1 mg/ml of BrdU in PBS was intraperitoneally injected to mice. Mice were sacrificed in 4 hrs after BrdU injection. The same segment of distal colon was fixed in 4% (w/v) paraformaldehyde and embedded by paraffin. Proliferating cells were detected with BrdU detection kit (BD Bioscience).

***In situ* hybridization**

For *in situ* hybridization, previously reported methods were used in this experiment (Su et al., 2014). Briefly, tissues of mice were fixed in 4% (w/v) paraformaldehyde-fixed and embedded in paraffin according to standard procedures. Briefly, 5 μm sections were deparaffinized, rehydrated through a graded series of ethanol baths, and washed with water treated with 0.1% DEPC three times for 5 min. In situ hybridization of LRRC19 was performed according to the manufacturer's protocol (TBD sci). Staining was conducted using a diaminobenzidine staining kit. Probes listed in Table S2d, were designed based on the published genomic sequences of mouse LRRC19 mRNA (GenBank ID: 224109). Probes were labeled with digoxin (DIG). The sense probes were used as negative controls.

**Gut microbiota analysis**

Gut microbiota was analyzed by Huada Biotechnology company (ShenZhen, China) using primers that target the V1-V3 regions of the 16S rRNA (Jeraldo et al., 2011). Once the PCR for each sample was completed, the amplicons were purified using the QIAquick PCR purification kit (Qiagen Valencia, CA), quantified, normalized, and then pooled in preparation for emulsion PCR followed by sequencing using Titanium chemistry (Roche, Basel Switzerland) according to the manufacturer's protocol. In the first step of data processing, the generated sequence data were de-convolved using the sample barcodes to identify sequences from each of the samples. Barcode, primer, and adaptor sequences were also trimmed as part of this step. PCR artifacts "chimeras" were identified using the ChimeraSlayer program (http://microbiomeutil.sourceforge.net; reference http://genome.cshlp.org/content/21/3/494.long) and removed prior to downstream analysis. The resulting de-convoluted and filtered sequence data were assigned taxonomy (to the genus level) using the Ribosomal Database Project (RDP)

classifier and the genera classifications were used to generate a sample-genus count matrix. Operational Taxonomic Unit (OTU) analysis of these sequences was performed as follows: sequences were processed (trimmed) using the Mothur software and subsequently clustered at 97% sequence identity using cd-hit to generate OTUs. The OTU memberships of the sequences were used to construct a sample-OTU count matrix. The samples were clustered at genus and OTU levels using the sample-genus and sample-OTU count matrices respectively. For each clustering, Morisita-Horn dissimilarity was used to compute a sample distance matrix from the initial count matrix, and the distance matrix was subsequently used to generate a hierarchical clustering using Ward's minimum variance method. The Wilcoxon Rank Sum test was used to identify OTUs that had differential abundance in the different sample groups.

Quantitave PCR (qPCR) was performed on genomic DNA extracted from the colon contents of WT or *LRRC19* KO mice as described above. qPCR was performed and analyzed using SYBR green I dye chemistry and 7300 Real-time PCR systems and software (Applied Biosystem).

**Preparation of adenovirus vectors**

All adenovirus vectors used in this study were produced by Applied Biological Materials (ABM) Inc..( Richmond, BC, Canada) and were amplified by Genechem Inc. Shanghai, China. ABM Inc.'s adenovirus CMV expression system provides an efficient method for constructing recombinant adenoviruses. This procedure uses *in vitro* ligation to subclone the gene of interest into a replication-incompetent (-E1/-E3) human adenoviral type 5 (Ad5) genome. First, the gene was cloned into pShuttle to producing recombinant adenoviral DNA. This process includes that PI-SceI/I-CeuI digestion of recombinant pShuttle, subclone the expression cassette of the pShuttle vector into the pAdeno genome, transform *E. coli* with ligation products and analyze the adenoviral DNA by PCR or restriction digestion. Then PI-SceI and I-CeuI restriction was analyzed and pAdeno DNA for the presence of pShuttle derived expression cassettes was screened by PCR with adeno forward and reverse PCR primers. Adenoviral DNA for transfection was prepared and adenoviruses were generated in 293 Packaging Cells. Finally, the recombinant adenoviral vectors were amplified and adenoviral titre was determined.

**Oligoes used in this study**

(a) Oligoes used in cloning LRRC19.

| Oligo name | Sequence (5'-3') | |
|---|---|---|
| MLRRC19F<br>MLRRC19R | ATGAAAGTCACACGCTTCATGTTTTGGC<br>CTTTTCTTCATGTACCTCATTGATATCT | For cloning murine LRRC19 |

(b) Oligoes used in RT-PCR (semi-quantitative RT-PCR).

| Oligo name | Sequence (5'>3') | Description |
|---|---|---|
| Murine LRRC19-s<br>Murine LRRC19-as | ATGAAAGTCACACGCTTCATG<br>AATTACTTTTCTTCATGTACCTCA | RT-PCR for murine full-length LRRC19 detection |
| GAPDH-s<br>GAPDH-as | GTGGCAAAGTGGAGATTGTTG<br>CAGTCTTCTGGGTGGCAGTGAT | RT-PCR for murine GAPDH detection |

(c) Oligos used for quantitative RT-PCR (qRT-PCR).

| Oligo name | Sequence (5'>3') | Description |
|---|---|---|

| | | |
|---|---|---|
| Murine GAPDH-s<br>Murine GAPDH-as | TCAACGGCACAGTCAAGG<br>TACTCAGCACCGGCCTCA | qRT-PCR for murine GAPDH detection |
| Murine β-actin-s<br>Murine β-actin-as | ATCATTGCTCCTCCTGAGCG<br>GCTGATCCACATCTGCTGGAA | qRT-PCR for murine β-actin detection |
| Murine LRRC19-s<br>Murine LRRC19-as | CCCAGATGAGCTAAAGCACGA<br>GAAAGCCCAGCTTTTCCCAAG | qRT-PCR for murine LRRC19 detection |
| Murine TNFα-s<br>Murine TNFα-as | GGTCTGGGCCATAGAACTGA<br>CAGCCTCTTCTCATTCCTGC | qRT-PCR for murine TNF-alpha detection |
| Murine IL-6-s<br>Murine IL-6-as | TCTGAAGGACTCTGGCTTTG<br>GATGGATGCTACCAAACTGGA | qRT-PCR for murine IL-6 detection |
| Murine IL-1β-s<br>Murine IL-1β-as | GTGTCTTTCCCGTGGACCTT<br>AATGGGAACGTCACACACCA | qRT-PCR for murine IL-1β detection |
| Murine IFNγ-s<br>Murine IFNγ-as | AACGCTTACACACTGCATCTTGG<br>GACTTCAAAGAGTCTGAGG | qRT-PCR for murine IFNγ detection |
| Murine IL-17-s<br>Murine IL-17-as | ACTACCTCAACCGTTCCACG<br>ATGTGGTGGTCCAGCTTTCC | qRT-PCR for murine IL-17 detection |
| Murine IL-12-s<br>Murine IL-12-as | TGGTTTGCCATCGTTTTGCTG<br>ACAGGTGAGGTTCACTGTTTCT | qRT-PCR for murine IL-12 detection |
| Murine CCL6 s<br>Murine CCL6 as | TATCCTTGTGGCTGTCCTTGG<br>TTACATGGGATCTGTGTGGCA | qRT-PCR for murine CCL6 detection |
| Murine CCL9-s<br>Murine CCL9-as | GCCCAGATCACACATGCAAC<br>AGGACAGGCAGCAATCTGAA | qRT-PCR for murine CCL9 detection |
| Murine CXCL9-s<br>Murine CXCL9-as | CCGAGGCACGATCCACTAC<br>AGGCAGGTTTGATCTCCGTT | qRT-PCR for murine CXCL9 detection |
| Murine CXCL10-s<br>Murine CXCL10-as | CAAGTGCTGCCGTCATTTTCT<br>ATAGGCTCGCAGGGATGATT | qRT-PCR for murine CXCL10 detection |
| Murine Reg3α-s<br>Murine Reg3α-as | CAAGGCTTATCGCTCCCACT<br>ACGAGATGTCCTGAGGGTCT | qRT-PCR for murine Reg3α detection |
| Murine Reg3β-s<br>Murine Reg3β-as | ATACCCTCCGCACGCATTAG<br>GGCCAGTTCTGCATCAAACC | qRT-PCR for murine Reg3β detection |
| Murine Reg3γ-s<br>Murine Reg3γ-as | TTCCTGTCCTCCATGATCAAA<br>CATCCACCTCTGTTGGGTTC | qRT-PCR for murine Reg3γ detection |
| Murine Reg4-s<br>Murine Reg4-as | CTGTGTGGATTGGCCTGCAT<br>GCAATGCCTGGCTTCACTCT | qRT-PCR for murine Reg4 detection |

(d) Probes used in *in situ* hybridization.

| Oligo name | Sequence (5’-3’) | Targeted sequence: |
|---|---|---|

| | | |
|---|---|---|
| Murine LRRC19 probe 1 | AGCTGTGGTGCATCGAAGAGGCGTATC | Murine LRRC19 in Gene-bank NM_175305 |
| Murine LRRC19 probe 2 | TGATAGCAATGAAGATAAGCAGTGAAGTT | Murine LRRC19 in Gene-bank NM_175305 |
| Murine LRRC19 probe 3 | CAGGCGATGGTGGTTGTAACTCAGCAG | Murine LRRC19 in Gene-bank NM_175305 |
| Mockprobe | Offered by company | |

(e) Oligoes used in identification of gut microbiota.

| Oligo name | Sequence (5'-3') | Description |
|---|---|---|
| Bacteroides Phylum | F: 5-GAGAGGAAGGTCCCCCAC-3<br>R: 5-CGCTACTTGGCTGGTTCAG-3 | rRNA-16S |
| Firmicutes Phylum | F: 5-GCTGCTAATACCGCATGATATGTC-3<br>R: 5-CAGACGCGAGTCCATCTCAGA-3 | rRNA-16S |

**3) Supplemental References:**

Chen, K., Liu, M., Liu, Y., Yoshimura, T., Shen, W., Le, Y., Durum, S., Gong, W., Wang, C., Gao, J.L., *et al.* (2013). Formylpeptide receptor-2 contributes to colonic epithelial homeostasis, inflammation, and tumorigenesis. The Journal of clinical investigation *123*, 1694-1704.

Greten, F.R., Eckmann, L., Greten, T.F., Park, J.M., Li, Z.W., Egan, L.J., Kagnoff, M.F., and Karin, M. (2004). IKKbeta links inflammation and tumorigenesis in a mouse model of colitis-associated cancer. Cell *118*, 285-296.

Jeraldo, P., Chia, N., and Goldenfeld, N. (2011). On the suitability of short reads of 16S rRNA for phylogeny-based analyses in environmental surveys. Environmental microbiology *13*, 3000-3009.

Lee, J., Mo, J.H., Katakura, K., Alkalay, I., Rucker, A.N., Liu, Y.T., Lee, H.K., Shen, C., Cojocaru, G., Shenouda, S., *et al.* (2006). Maintenance of colonic homeostasis by distinctive apical TLR9 signalling in intestinal epithelial cells. Nature cell biology *8*, 1327-1336.

Su, X., Min, S., Cao, S., Yan, H., Zhao, Y., Li, H., Chai, L., Mei, S., Yang, J., Zhang, Y., *et al.* (2014). LRRC19 expressed in the kidney induces TRAF2/6-mediated signals to prevent infection by uropathogenic bacteria. Nature communications *5*, 4434.
